# Selection in Reported Epidemiological Risks: An Empirical Assessment

**DOI:** 10.1371/journal.pmed.0040079

**Published:** 2007-03-06

**Authors:** Fotini K Kavvoura, George Liberopoulos, John P. A Ioannidis

**Affiliations:** 1 Clinical and Molecular Epidemiology Unit, Department of Hygiene and Epidemiology, University of Ioannina School of Medicine, Ioannina, Greece; 2 Department of Medicine, Tufts University School of Medicine, Boston, Massachusetts, United States of America; McGill University, Canada

## Abstract

**Background:**

Epidemiological studies may be subject to selective reporting, but empirical evidence thereof is limited. We empirically evaluated the extent of selection of significant results and large effect sizes in a large sample of recent articles.

**Methods and Findings:**

We evaluated 389 articles of epidemiological studies that reported, in their respective abstracts, at least one relative risk for a continuous risk factor in contrasts based on median, tertile, quartile, or quintile categorizations. We examined the proportion and correlates of reporting statistically significant and nonsignificant results in the abstract and whether the magnitude of the relative risks presented (coined to be consistently ≥1.00) differs depending on the type of contrast used for the risk factor. In 342 articles (87.9%), ≥1 statistically significant relative risk was reported in the abstract, while only 169 articles (43.4%) reported ≥1 statistically nonsignificant relative risk in the abstract. Reporting of statistically significant results was more common with structured abstracts, and was less common in US-based studies and in cancer outcomes. Among 50 randomly selected articles in which the full text was examined, a median of nine (interquartile range 5–16) statistically significant and six (interquartile range 3–16) statistically nonsignificant relative risks were presented (*p* = 0.25). Paradoxically, the smallest presented relative risks were based on the contrasts of extreme quintiles; on average, the relative risk magnitude was 1.41-, 1.42-, and 1.36-fold larger in contrasts of extreme quartiles, extreme tertiles, and above-versus-below median values, respectively (*p* < 0.001).

**Conclusions:**

Published epidemiological investigations almost universally highlight significant associations between risk factors and outcomes. For continuous risk factors, investigators selectively present contrasts between more extreme groups, when relative risks are inherently lower.

## Abstract 2

An evaluation of published articles reporting epidemiological studies found that they almost universally highlight significant associations between risk factors and outcomes.

## Editors' Summary

**Background.:**

Medical and scientific researchers use statistical tests to try to work out whether their observations—for example, seeing a difference in some characteristic between two groups of people—might have occurred as a result of chance alone. Statistical tests cannot determine this for sure, rather they can only give a probability that the observations would have arisen by chance. When researchers have many different hypotheses, and carry out many statistical tests on the same set of data, they run the risk of concluding that there are real differences where in fact there are none. At the same time, it has long been known that scientific and medical researchers tend to pick out the findings on which to report in their papers. Findings that are more interesting, impressive, or statistically significant are more likely to be published. This is termed “publication bias” or “selective reporting bias.” Therefore, some people are concerned that the published scientific literature might contain many false-positive findings, i.e., findings that are not true but are simply the result of chance variation in the data. This would have a serious impact on the accuracy of the published scientific literature and would tend to overestimate the strength and direction of relationships being studied.

**Why Was This Study Done?:**

Selective reporting bias has already been studied in detail in the area of randomized trials (studies where participants are randomly allocated to receive an intervention, e.g., a new drug, versus an alternative intervention or “comparator,” in order to understand the benefits or safety of the new intervention). These studies have shown that very many of the findings of trials are never published, and that statistically significant findings are more likely to be included in published papers than nonsignificant findings. However, much medical research is carried out that does not use randomized trial methods, either because that method is not useful to answer the question at hand or is unethical. Epidemiological research is often concerned with looking at links between risk factors and the development of disease, and this type of research would generally use observation rather than experiment to uncover connections. The researchers here were concerned that selective reporting bias might be just as much of a problem in epidemiological research as in randomized trials research, and wanted to study this specifically.

**What Did the Researchers Do and Find?:**

In this investigation, searches were carried out of PubMed, a database of biomedical research studies, to extract epidemiological studies that were published between January 2004 and October 2005. The researchers wanted to specifically look at studies reporting the effect of continuous risk factors and their effect on health or disease outcomes (a continuous risk factor is something like age or glucose concentration in the blood, is a number, and can have any value on a sliding scale). Three hundred and eighty-nine original research studies were found, and the researchers pulled out from the abstracts and full text of these papers the relative risks that were reported along with the results of statistical tests for them. (Relative risk is the chance of getting an outcome, say disease, in one group as compared to another group.) The researchers found that nearly 90% of these studies had one or more statistically significant risks reported in the abstract, but only 43% reported one or more risks that were not statistically significant. When looking at all of the findings reported anywhere in the full text for 50 of these studies, the researchers saw that papers overall reported more statistically significant risks than nonsignificant risks. Finally, it seemed that in the set of papers studied here, the way in which statistical analyses were done produced a bias towards more extreme findings: for datasets showing small relative risks, papers were more likely to report a comparison between extreme subsets of the data so as to report larger relative risks.

**What Do These Findings Mean?:**

These findings suggest that there is a tendency among epidemiology researchers to highlight statistically significant findings and to avoid highlighting nonsignificant findings in their research papers. This behavior may be a problem, because many of these significant findings could in future turn out to be “false positives.” At present, registers exist for researchers to describe ongoing clinical trials, and to set out the outcomes that they plan to analyze for those trials. These registers will go some way towards addressing some of the problems described here, but only for clinical trials research. Registers do not yet exist for epidemiological studies, and therefore it is important that researchers and readers are aware of and cautious about the problem of selective reporting in epidemiological research.

**Additional Information.:**

Please access these Web sites via the online version of this summary at http://dx.doi.org/10.1371/journal.pmed.0040079.

- Wikipedia entry on publication bias (note: Wikipedia is an internet encyclopedia that anyone can edit)
- The International Committee of Medical Journal Editors gives guidelines for submitting manuscripts to its member journals, and includes comments about registration of ongoing studies and the obligation to publish negative studies
- ClinicalTrials.gov and the ISRCTN register are two registries of ongoing clinical trials

## Introduction

Researchers sometimes selectively present their findings, focusing on the more impressive aspects of their work. Focusing on impressive aspects means that researchers may try to show statistically significant results and/or larger effect sizes. Testing for statistical significance is not necessarily a bad thing, even though the process has been criticized [1]. In theory, it can help keep chance findings out of the literature. However, problems ensue when significance testing is accompanied by selective reporting, and we do not know how many hypotheses have been examined and in how many different ways the data have been analyzed. Some studies with statistically nonsignificant (“negative”) results may remain unpublished (publication bias) [2–4] or may be published with delay compared with statistically significant (“positive”) studies (time-lag bias) [5,6]. Bias may also affect the reporting of results within studies: “positive” outcomes may be reported preferentially over potentially less appealing “negative” analyses, even if this distorts the original analysis plan [7,8]. Emphasis may be given to post-hoc subgroup analyses [9] or to dubious adjustments [10,11] that claim statistical significance [11].

While these biases have been studied predominantly in the randomized trials literature, selective reporting may be more prominent in epidemiological research [4]. Moreover, reporting of epidemiological results lacks standardization [12,13]. Thus, there is plenty of room for selective reporting. This is difficult to prove without access to the original protocols of these studies while, sometimes, protocols may not even exist. Refutations of epidemiological associations [14–17] pose the question as to whether biased findings are common. One may obtain some indirect evidence by examining the presented results of epidemiological studies. If most presented results are “positive” and few are “negative,” this may offer indirect evidence for selective reporting.

Moreover, one may examine how epidemiological estimates of risk are presented in the literature. Is there a preference to show larger magnitudes of effect? For risk factors that take continuous values, the presented magnitude of the relative risk may depend on the selected contrast. The risk may be presented per unit change, per standard deviation, or according to a contrast of specific percentile groups of the values of the postulated risk factor. The latter option is very popular. Typical contrasts involve splitting the data into quintiles, quartiles, tertiles, and above-versus-below the median value of the risk factor. Unless a risk relationship is J- or U-shaped, when the compared groups are further apart, the estimated relative risk would deviate further from the “null” value of 1.00. For example, if consumption of a nutrient is associated with the risk of prostate cancer, then the risk ratio may be 1.10 when values of above-versus-below the median are compared, but in the same data, the risk ratio may be 1.50 if extreme quintiles are compared. When relative risks are inherently more modest, do investigators select to compare groups that are further apart?

Here we examined empirically a sample of published articles on epidemiological studies that presented relative risk estimates for continuous risk factors with percentile-based contrasts. The analysis had two objectives. First, we aimed to estimate how many articles highlight “positive” or “negative” results and to identify correlates thereof. Second, it was our intention to identify whether the magnitude of the highlighted risk was related to the selected percentile contrast for the risk factor.

## Methods

### Eligible Studies and Search Strategy

We assembled a systematic sample of recent epidemiological articles that presented, in their respective abstracts, at least one relative risk for at least one continuous risk factor in contrasts based on median, tertile, quartile, or quintile categorizations. The following seven contrasts were considered eligible: above-versus-below median values; extreme tertiles; extreme tertile versus remaining subjects; extreme quartiles; extreme quartile versus remaining subjects; extreme quintiles; and extreme quintile versus remaining subjects. A pilot literature screen suggested that other percentile-based categorizations are uncommon. Contrasts between two extreme groups were considered eligible, regardless of whether estimates were also presented for contrasts between one extreme and one intermediate group. For example, the extreme quartiles category considers all studies in which a relative risk for the contrast of extreme quartiles is presented, regardless of whether data are presented for the contrast of the third versus first quartile and the second versus first quartile. Whenever the contrast was not clear from the abstract, we searched the full article. We did not consider slope estimates, i.e., a change in odds ratio for each unit change in exposure, since this does not require any choice for categorization of exposure levels.

We accepted all multiplicative-effect metrics as measures of relative risk, including hazard ratios, incidence rate ratios, risk ratios, and odds ratios. We did not consider studies where a continuous outcome, rather than the postulated risk factor, was categorized according to some percentile grouping. We considered epidemiological studies regardless of their design, but excluded meta-analyses, as we focused on primary studies. We used a search strategy that would favor the selection of cohort studies. Cohort studies are traditionally the most definitive type of epidemiological investigation. However, they are less common in the literature than case-control and cross-sectional designs. To enrich our sample with cohort designs, we searched PubMed (http://www.ncbi.nlm.nih.gov/entrez/query.fcgi?DB=pubmed), combining the term cohort* with quintile*, quartile*, tertile*, or (median AND [above OR below]) as words in the abstract. Only English-language articles were considered. We included all eligible retrieved articles published in a period starting 1 January 2004 and indexed until the last search update (28 October 2005).

### Data Extraction

For each article that reported, in the abstract, at least one numerical relative risk estimate for any of the seven eligible percentile-based contrasts, we recorded the first author, journal of publication, impact factor of the journal (according to Journal Citation Reports [18]), first country listed in the PubMed record, cohort of origin, presence of any structure (section headings) in the abstract, and design. Design was broadly categorized as cohort design, reporting any metric other than odds ratio (e.g., hazard ratio or incidence rate ratio); case-control design, reporting odds ratio; and other cohorts of subjects with reported odds ratios (including cross-sectional studies and studies with logistic regression analyses, but without clearly defined cases and controls). We also recorded whether any statistically significant relative risk (for any continuous or discrete risk factor and for any type of contrast) was reported numerically in the abstract. Similarly, we recorded whether any statistically nonsignificant relative risk was reported numerically in the abstract. Statistical significance was inferred from *p*-values (0.05 threshold) or 95% confidence intervals (CIs).

We also extracted detailed data for one eligible relative risk per study. Epidemiological studies often test numerous hypotheses and estimate numerous relative risks. Investigators may mention in the abstract what they consider to be the key findings of their work. To avoid subjective selection on our part, we always chose the first eligible relative risk presented numerically in the abstract. The choice of the first presented relative risk has been adopted also in previous empirical research on epidemiological studies [12]. For this relative risk, we noted the type of contrast, point estimate, 95% CI, whether or not it was formally statistically significant, tested risk factor, and outcome/endpoint assessed.

Risk factors were categorized as dietary intake and dietary patterns; toxic exposures and markers thereof; biological markers in any biological fluid or tissue; psychological, behavioral, or social factors; physical activity or energy expenditure; body characteristics and composition; and other. Outcomes were classified as mortality versus non-mortality; the latter were further classified into malignancies, vascular (including cardiac, cerebrovascular, and other vascular), and other.

We did not record the sample size of each study, because the pertinent sample for each presented relative risk might differ from the overall sample. Instead, we calculated the standard error of the natural logarithm of the relative risk. The standard error is given by the absolute value of the difference between upper and lower 95% CI divided by 3.92. The inverse of the standard error is a more appropriate measure of precision than plain sample size.

Abstracts may be biased towards reporting the most significant results, but such selection may not affect the information available in the full text. Moreover, another issue concerns whether the predominance of significant results in the abstract might be less prominent if all presented relative risks were examined rather than the first one alone. Therefore, for sensitivity analyses, we also evaluated 50 randomly selected articles in more depth, where we recorded all the respective relative risks presented in both the abstract and in the full text.

All data were extracted in duplicate by two independent investigators. Discrepancies were resolved by consensus.

### Analyses

We evaluated the proportion of studies that had at least one statistically significant relative risk estimate presented numerically in the abstract. We examined whether this was related to any of the study characteristics mentioned above. We first performed univariate logistic regressions. In multivariate models, a variable was considered initially only if it had *p* < 0.25 in the univariate analysis; we then used backward elimination using likelihood ratio criteria with a *p* = 0.05 threshold. All analyses that we performed for all variables considered are shown in tabulated form for the odds ratios derived from the regression coefficients and their 95% CIs. No varying forms models or complex interaction terms were evaluated. Similarly, we examined whether there was any relationship between these specific variables and the reporting of at least one statistically nonsignificant relative risk.

In sensitivity analyses using full texts (50 articles), we used Wilcoxon rank sum tests to examine whether the number of significant relative risks presented in the abstract and in the full text of results exceeded the nonsignificant ones. We tested (using McNemar and Wilcoxon signed rank tests) whether the proportion of significant relative risks and the magnitude of the relative risks were different, and when the first eligible relative risk presented in the text was selected, rather than the time of selecting the first eligible relative risk presented in the abstract.

Analyses of the magnitude of the presented relative risks used only the first eligible relative risk per study presented in the abstract. We coined all relative risk estimates in such a way that they would be ≥1.00; i.e., relative risks <1.00 were inverted so as to focus consistently on the extent of deviation from the “null.” Values were log-transformed. Analysis of variance (ANOVA) compared first the four contrasts of two extreme groups (extreme quintiles, extreme quartiles, extreme tertiles, and above-versus-below median values). Then, we considered both types of contrasts (comparison of the two extreme groups or comparison of one extreme group versus all the remaining subjects) and type of percentile; the above-versus-below median contrast was not relevant in this analysis.

Finally, we examined with ANOVA whether the choice of contrast was related to the degree to which a study was well powered, i.e., whether studies chose contrasts comparing more extreme groups, and when they had more sufficient power (lower standard errors of the relative risk estimates).

Analyses were conducted in SPSS 13.0 (http://www.spss.com). *p*-Values are two-tailed.

## Results

### Eligible Studies

**Figure 1:**
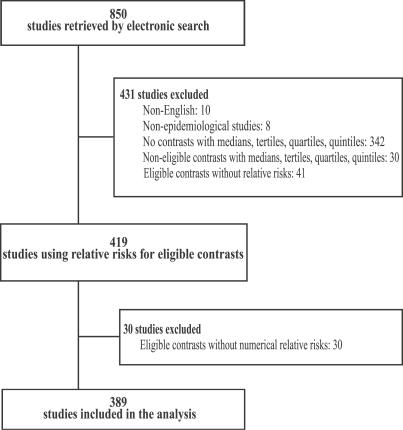
Flow Diagram for Studies Included or Excluded from the Analyses According to Eligibility Criteria

The electronic search yielded 850 articles; 461 were rejected upon screening (Figure 1) and 389 articles were eligible (Tables 1 and S1). Epidemiology and nutrition journals were common venues of publication for these studies, but many specialty clinical journals also published such studies, and 28 articles appeared in major general journals *(Annals of Internal Medicine* [*n* = 5], *Archives of Internal Medicine* [*n* = 12], *CMAJ: Canadian Medical Association Journal (Journal de l'Association Medicale Canadienne)* [*n* = 1], *JAMA: The Journal of the American Medical Association* [*n* = 7], and *New England Journal of Medicine* [*n* = 3], although none appeared in *Lancet* or *BMJ).* Almost a quarter of the 389 studies appeared in journals that have an impact factor of above seven. Half of the 389 studies originated from the United States. Twenty-four cohorts contributed more than one article (totaling 152 articles, 39.1%) to the eligible 389 articles. In particular, 54 articles (13.9%) originated from the large cohorts established at Harvard University (i.e., Nurses' Health Study/Physicians' Health Study/Health Professionals' Follow-up Study), followed by ARIC (Atherosclerosis Risk in Community, *n* = 11) and EPIC (European Prospective Investigation into Cancer and Nutrition, *n* = 10). Typical cohorts were overrepresented, as intended. Structured abstracts were very common. The comparison of extreme quartiles was the most frequent type of contrast among the first presented relative risks. Contrasts of extreme tertiles and extreme quintiles were also common. Contrasts of one extreme group against the remaining subjects were far less frequent.

**Table 1:**
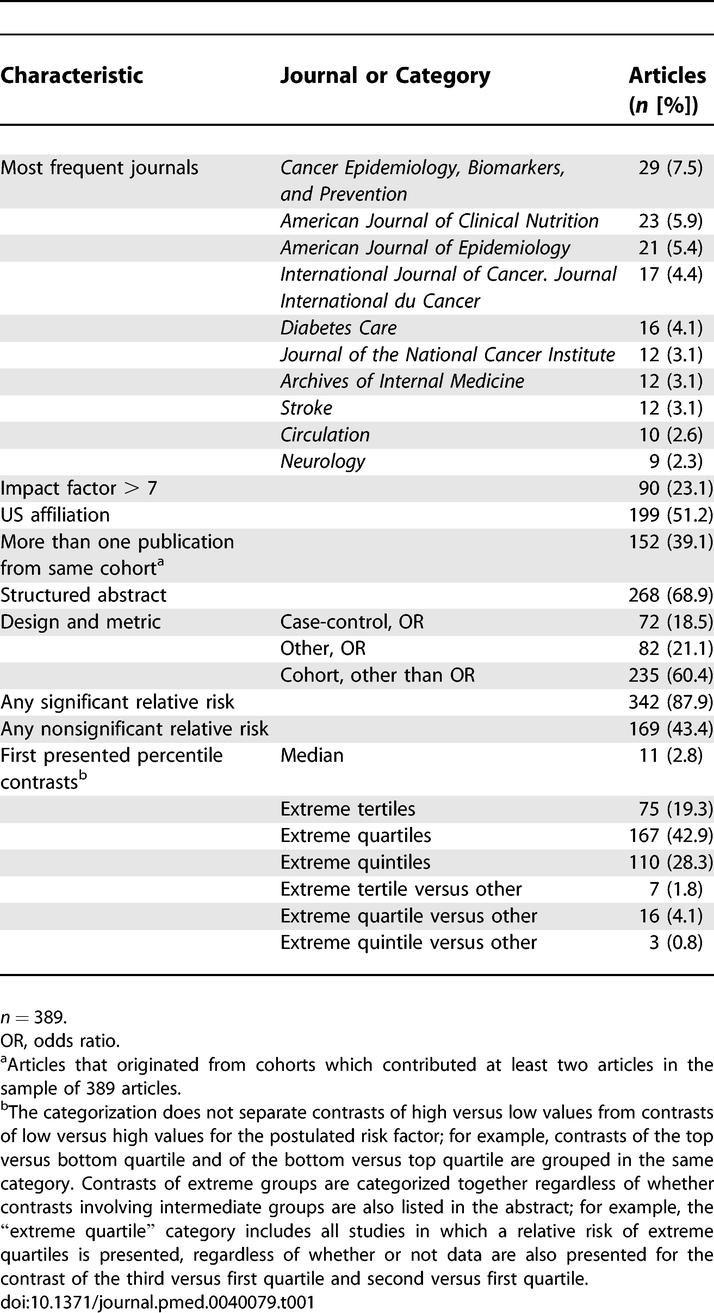
Characteristics of Analyzed Studies

### First Reported Relative Risks

The median was 1.73 (interquartile range [IQR] 1.39–2.48) when all relative risks were coined to be ≥1.00, and only 22 of these relative risks (5.7%) exceeded 5.0. Four-fifths of these relative risks were statistically significant (Table 2). Among risk factors, biological markers and dietary factors accounted together for more than two-thirds of the studies. Non-mortality outcomes were involved in the vast majority of studies (Table 2).

**Table 2:**
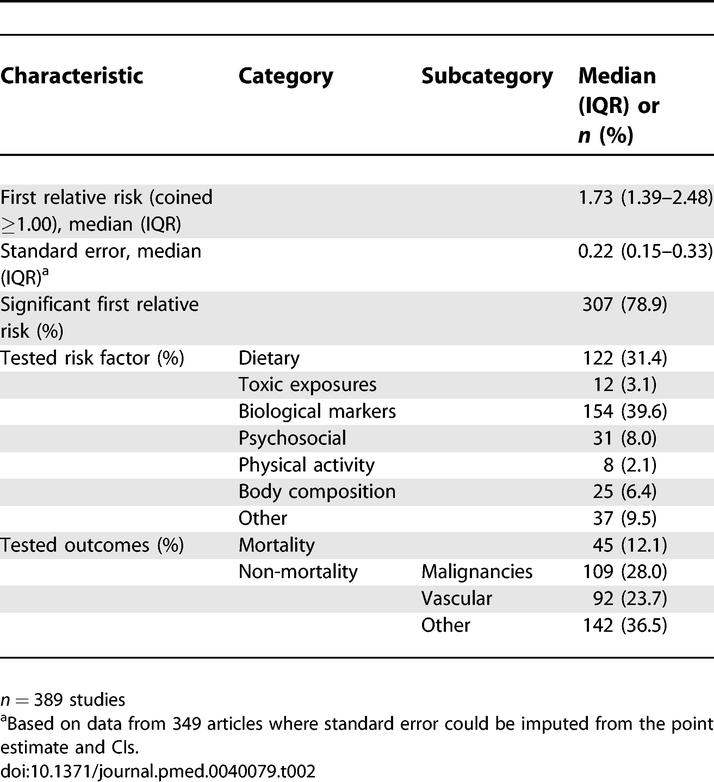
Analyzed Eligible Relative Risks

### Correlates of Significant and Nonsignificant Relative Risks

Almost nine out of ten articles (342/389; 87.9%) reported ≥1 statistically significant relative risk in the abstract. The proportion of abstracts with ≥1 statistically nonsignificant relative risk was only 169/389 (43.4%). Among 30 excluded articles where otherwise eligible relative risks appeared, but without exact numerical information, proportions were 93.3% and 56.7%, respectively.

Reporting at least one statistically significant risk in the abstract was positively related to the presence of a structured abstract, and it was less likely in studies originating from the United States and in studies of cancer outcomes in both univariate and multivariate analyses (Table 3). Conversely, reporting at least one nonsignificant relative risk in the abstract was more likely in studies originating from the United States and in studies of cancer outcomes. Moreover, the reporting of “negative” results was also related to the type of risk factor: studies on psychosocial risk factors or body composition and characteristics were even less likely to report any statistically nonsignificant risk (Table 3).

**Table 3:**
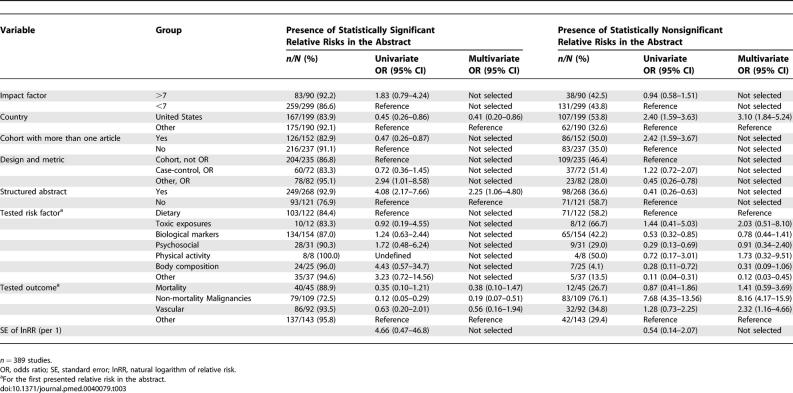
Logistic Regressions for Presence of Significant Relative Risks and Nonsignificant Relative Risks

### Evaluation of Full Texts

In the 50 randomly selected articles evaluated in depth, the abstracts had a median of two statistically significant relative risks (IQR 1–3) versus zero nonsignificant risks (IQR 0–1). A preponderance of statistically significant relative risks was seen in 33 articles (66%), a preponderance of nonsignificant risks was noted in five articles (10%), and equal numbers were seen in 12 articles (24%) (Wilcoxon, *p* < 0.001).

In the full text, the median number of articles reporting any statistically significant relative risk was nine (IQR 5–16) versus six articles reporting nonsignificant risks (IQR 3–16). In 28 articles (56%), more statistically significant relative risks were reported than statistically nonsignificant relative risks. In 20 articles (40%), there were more nonsignificant relative risks than significant ones, and in two articles (4%) there was an equal number of significant and nonsignificant relative risks (Wilcoxon, *p* = 0.25). None of the 50 articles reported only a single relative risk. Only four out of the 50 articles claimed to be the first study assessing a specific association, while a further five articles claimed to be the first to address a previously tested association in a new setting (based on population characteristics or type of study design).

The first reported relative risk with eligible contrast in the full text was formally statistically significant in 35 articles (70%). The median relative risk was 1.54 (IQR 1.30–2.43), when relative risks were consistently coined to be ≥1. These values are similar to the respective values for the first reported relative risk with eligible contrast in the abstract (McNemar, *p* = 0.15 for significant results; Wilcoxon, *p* = 0.12 for median coined relative risk).

In three articles, the first reported relative risk was not formally significant, but the authors nevertheless interpreted this as an association. The inverse interpretation (i.e., formally significant risk interpreted as nonsignificant by the authors) was not seen in any of these 50 articles.

### Magnitude of Risk for Different Contrasts

**Figure 2:**
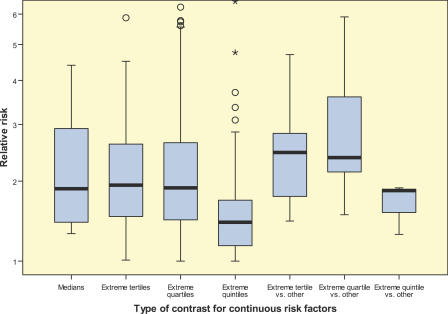
Box Plots for Relative Risks for Different Contrasts of the Values of the Postulated Risk Factor All relative risks have been coined to be ≥1.00 for consistency.

Figure 2 shows the distribution of relative risks (coined to be ≥1.00) according to the type of contrast used to present the postulated risk. Contrary to what would be expected, the presented effects were smaller, on average, when the compared groups of the postulated risk factor were further apart. The smallest effects were described with the contrast of extreme quintiles. Compared with the contrasts of extreme quintiles, the relative risks were significantly larger in contrasts of extreme quartiles (1.41-fold larger), extreme tertiles (1.42-fold larger), and above-versus-below median (1.36-fold larger) (ANOVA, *p* < 0.001).

Moreover, the presented effects were smaller, on average, when extreme groups were compared than when one extreme group was compared against the remaining subjects (Figure 2). The relative risks were 0.81-fold lower (*p* = 0.044) for comparisons of the two extreme groups versus comparisons of one extreme group against the remaining subjects after adjusting for type of percentile involved in the contrast (1.37-fold, and 1.36-fold larger relative risks with tertile contrasts and quartile contrasts, respectively, compared with quintile contrasts, *p* < 0.001). Only six studies referred explicitly to a J- or U-shaped or nonlinear effect. Exclusion of these studies did not change our results (unpublished data).

### Study Precision for Different Contrasts

Compared with contrasts of above-versus-below the median, the standard error of the relative risks was similar, on average, in studies with contrasts of extreme tertiles (on average 1% larger) or extreme quartiles (on average 8% smaller). The standard error was 49% lower on average when extreme quintiles were contrasted (ANOVA, *p* < 0.001). Thus, precision was maximal when the compared extreme groups pertained to the smallest possible portion (extreme quintiles, 40%) of the study population.

## Discussion

Epidemiological investigations almost universally highlight significant associations between risk factors and outcomes. The vast majority of the 389 articles that we analyzed reported some significant results. Less than half of these articles presented at least one nonsignificant relative risk in their respective abstracts. This pattern suggests that there is a strong predilection for highlighting “positive” results and avoiding “negative” results. The preponderance of significant findings was less prominent in the full texts of these articles. However, even in the full texts, an article reported, on average, at least as many significant relative risks as nonsignificant ones, and sometimes reported a greater number. Despite some variability depending on country, tested risk factor, and outcome, most important fields of epidemiological investigation seem to have little room for “negative” findings.

Given the largely exploratory nature of most epidemiological analyses, one would expect that most hypotheses analyzed should be “negative.” A counter argument, however, is that epidemiologists do not select null hypotheses at random, but rather because there is some reason to believe they are false. For most of the reported relative risks considered in the present paper, there were already pertinent results available, perhaps needing replication and refinement. However, even when studies were not the first to report on a tested hypothesis, there is no guarantee that previous studies had found “positive” results, and the fact that a hypothesis is tested repeatedly does not guarantee its credibility [19,20]. The exact expected proportion of statistically significant associations in the entire field of epidemiological research is, by default, unknown. However, we can gain some insights into epidemiological research by examining the rate of replication of epidemiological findings, each time similar hypotheses are tested, using very large, well-conducted studies, preferably with the most robust designs, such as randomized trials.

Empirical evidence shows than even among the most cited, confirmatory epidemiological studies, five out of six studies have been refuted or were found to be exaggerated within a few years of their publication in major journals [14]. In modern epidemiology, we also have evidence that most proposed associations are rejected when large-scale evidence accumulates. For example, of 32 candidate gene associations that proposed that common gene variants were associated with breast cancer, large-scale evidence eventually indicated that none remained formally significant after correcting for 32 comparisons, and only a few associations maintained an uncorrected *p*-value of less than 0.05 [21,22]. Another argument comes from the sheer number of available epidemiological factors under study. For example, we can currently test millions of genetic variants and a vast number of exposures. Even considering only independent variants and independent exposures, the claimed associations already could explain several-fold more than 100% of the attributable fraction for each outcome. This was already an issue almost three decades ago when Doll and Peto tried to estimate attributable fractions for cancer risk factors [23], and the scale of the problem has escalated in modern epidemiology.

Our results extend the observation of a previous survey in which 63 of 73 epidemiological studies in leading journals had statistically significant results [12]. Three quarters of the analyzed risk relationships reflected effect sizes where the compared groups differ less than 2.5-fold in their risk for the outcome of interest. Relative risks exceeding five were very rare. On the whole, the current literature presents modest associations, and half of them cluster in the relatively narrow relative risk range of 1.4–2.5. For some fields, the typical relative risks may be even lower. The strength of an observed epidemiological association is one of the classic criteria for causality.

We observed a lower frequency of significant results and a higher frequency of nonsignificant results in US studies. It has been previously reported [24] that studies from non-English speaking countries may report significant results more frequently in the English literature. Nonsignificant results may be reserved for the local non-English literature that is typically not indexed in PubMed. However, the direction of “language bias” may vary across different fields [25,26]. In our sample, relatively few articles were from English-speaking countries other than the United States. The association of presented significant results with structured abstracts may reflect the possibility that structured abstracts may encourage the use of exact numbers. Finally, the association of fewer significant results with cancer outcomes may reflect a larger prevalence of “negative” findings in cancer epidemiology compared with other fields, such as cardiovascular disease. Alternatively, it could be speculated whether there are more journals specializing in cancer epidemiology.

Furthermore, we noted that when the compared groups were further apart in the distribution of the values of the risk factor, the presented relative risks were lower. The contrast of extreme quintiles, the most extreme contrast of those evaluated here, was used to present what were, on average, the smallest relative risks. Investigators presented more extreme contrasts when the risks were inherently lower. In fact, studies with extreme contrasts apparently had been designed upfront to have more power to detect small effects than studies that reported more proximal contrasts. It could be argued that there is nothing wrong with epidemiologists designing contrasts that are more likely to reveal the relationship that is being sought, provided the contrasts are transparently reported. However, most non-methodologist readers may still be misled. For example, by comparing the extreme quintiles, a relative risk of 1.5 may be calculated, while a contrast of people with above-versus-below median values might have given a relative risk of 1.2, or even 1.1, for the same dataset. The non-methodologist reader or the general public would then be informed about a 50% relative increase in the disease risk, rather than 20% or 10%—a more impressive result that nevertheless pertains to the fewer people of the extreme groups. Most readers and even physicians may not understand that, with extreme groups being compared, the presented risk pertains to only a minority of people in the population. The use of relative risk metrics, rather than measures of absolute risk, may cause further misinterpretations and has been characterized as a main source of confusion in understanding medical statistics [27–29]. The problem is heightened when relative risks seem even larger, because many apparently sizeable relative risks eventually translate to negligible absolute risks [28,29].

We should also acknowledge that researchers and editors may try to select and present what they deem to be the most interesting and important work. The window on the world offered by the published scientific literature is not comprehensive, but instead is a particular view reflecting a host of complicated desires, abilities, and interests of the scientific community. Whether statistical significance should be one of the criteria used to select work for presentation has been a point of endless debate. However, at a minimum, if data are selected based on significance thresholds, it is important to know the underlying multiplicity of the conducted analyses. A significant risk (for example, *p* < 0.05) that arises out of a single hypothesis and a single analysis is very different than one that arose out of a massive screening of potential risk factors where it is not shown that many other risk factors have also been screened.

Some additional limitations should be discussed. First, we focused on articles that used specific percentile group contrasts; this was dictated by our aim to investigate specific selection biases based on these contrasts. We encourage assessments of other designs (e.g., binary risk factors and non-percentile contrasts); preliminary evidence suggests that the pursuit of statistical significance exists across all epidemiological studies [12]. Second, no data existed with which to compare presented relative risks with different types of percentile contrasts in the same study because, with very rare exceptions, each study used only one type of contrast.

Selection in primary studies could also affect the findings and inferences of secondary analyses, leading to spurious conclusions being drawn. A meta-analysis may ameliorate this defect by using the raw (individual level) data without taking into consideration the selected contrasts that had been presented in each paper. However, this would require full access to all data, and this is currently the exception. Selection of outcomes and contrasts in the primary studies may lead to similar selective choices in meta-analyses that have to depend on published data. This would further perpetuate these biases. Meta-analyses may try to detect and address these selective reporting problems using a variety of diagnostic tools such as asymmetry tests. However, having an unbiased body of evidence is certainly preferable to trying to detect and eliminate bias after the fact.

Our empirical findings may lead to some recommendations on how to improve the situation. Epidemiological research is very important [30], but reporting of epidemiological studies needs standardization [12,13,31–33], as has been proposed for clinical trials and other study designs [34–36]. The “STrengthening the Reporting of OBservational studies in Epidemiology” (STROBE) statement and similar efforts in genetic epidemiology are working in this direction. Investigators should avoid the selective presentation, and dissemination, of high-risk estimates and significant results. They should give a clear explanation of the way in which quantitative exposures are analyzed e.g., which groupings are chosen, whether a continuous analysis is done, and the rationale behind the choice (continuous, trend test, or comparison to a reference group) [37]. In particular, they should avoid estimating results with various categorical contrasts and selecting what to report simply based on the seemingly largest magnitude of effect. Readers should also be advised to interpret cautiously apparently large effects that are based on extreme contrasts and should instead place them properly in the population context. Study reports should also convey the exact breadth of analyses that have been performed. While it may not be possible to provide all “negative” results in detail, the reader should be aware of the existence of these analyses that have led to “negative” results. This is fairly challenging because, in contrast with randomized trials [38], upfront registration of epidemiological protocols may be very difficult or unrealistic. Some epidemiological research will unavoidably remain exploratory and post hoc in nature. Even so, this exploratory nature would need to be clarified, and selective reporting minimized, so that epidemiological findings could be interpreted in the most appropriate perspective.

## Supporting Information

Table S1 References and Tabulated Key Information on the 389 Studies (162 KB PDF)

## Abbreviations

ANOVA: analysis of variance
CI: confidence interval
IQR: interquartile range
