# Supplementary material for: Selection in Reported Epidemiological Risks: An Empirical Assessment
Source: PLoS Med. 2007 Mar 6;4(3):e79. doi: 10.1371/journal.pmed.0040079 (PMC1808481; doi:10.1371/journal.pmed.0040079)
Supplement: Supplementary file 1 [file pmed.0040079.st001.pdf]

| ID | AUTHOR | YEAR | JOURNAL | COUNTRY | ANY SIGNIFICANT | ANY NON SIGNIFICANT | CONTRAST | RELATIVE RISK | SIGNIFICANCE | RISK FACTOR | OUTCOME |
|---|---|---|---|---|---|---|---|---|---|---|---|
| 4003 | Fried LF | 2005 | J Am Soc Nephrol | USA | 1 | 0 | qua 4 vs 1 | 1.69 | 1 | cystatin C | noncardiovascular mortality |
| 4004 | Messer LC | 2005 | Matern Child Health J | USA | 1 | 1 | qua 4 vs 1 | 3.4 | 1 | pregnancy intendness | levels of perceived stress during pregnancy |
| 4006 | Butler LM | 2005 | Am J Respir Crit Care Med | USA | 1 | 0 | qua 4 vs 1 | 1.43 | 1 | meat-dim sum pattern | new-onset cough with phlegm |
| 4008 | Cocco P | 2005 | Cancer Res | Italy | 0 | 1 | qua 4 vs 1 | 2.00 | 0 | dichlorodiphenyltrichloroethane | stomach cancer mortality |
| 4010 | Li G | 2005 | Neurology | USA | 0 | 1 | qua 4 vs 1 | 1.16 | 0 | total cholesterol | dementia |
| 4013 | Larsson SC | 2005 | Am J Clin Nutr | Sweden | 1 | 0 | qua 4 vs 1 | 0.59 | 1 | high-fat dairy foods/d | colorectal cancer |
| 4016 | Beddhu S | 2005 | J Ren Nutr | USA | 1 | 0 | qua 4 vs 1 | 1.89 | 1 | total protein intake | low serum albumin |
| 4019 | Thorand B | 2005 | Diabetes | Germany | 1 | 0 | qua 4 vs 1 | 1.73 | 1 | IL-18 | type 2 diabetes |
| 4020 | Luchtenborg M | 2005 | Cancer Causes Control | Netherlands | 1 | 1 | qua 4 vs 1 | 1.61 | 0 | Meat product consumption | colon tumours with truncating APC mutation |
| 4021 | Park HA | 2005 | Atherosclerosis | USA | 1 | 1 | qua 4 vs 1 | 1.99 | 1 | saturated fat intake | coronary calcification |
| 4022 | Koushik A | 2005 | Cancer Epidemiol Biomarkers Prev | USA | 0 | 1 | qua 4 vs 1 | 1.06 | 0 | fruit intake | ovarian cancer |
| 4023 | Rock CL | 2005 | J Clin Oncol | USA | 1 | 0 | qua 4 vs 1 | 0.57 | 1 | plasma total carotenoid concentration | new breast cancer event |
| 4025 | Sornay-Rendu E | 2005 | J Bone Miner Res | France | 1 | 0 | qua 4 vs 1 | 2.20 | 1 | bone alkaline phosphatase | fracture |
| 4030 | Wakai K | 2005 | Cancer Sci | Japan | 1 | 1 | qua 4 vs 1 | 0.80 | 0 | total fat intake | breast cancer |
| 4031 | Ito Y | 2005 | J Epidemiol | Japan | 1 | 0 | qua 4 vs 1 | 0.41 | 1 | serum alpha-carotenes | lung cancer death |
| 4034 | Merkin SS | 2005 | Am J Kidney Dis | USA | 1 | 0 | qua 4 vs 1 | 1.60 | 1 | Individual-level socioeconomic status area | progressive Chronic kidney disease |
| 4036 | Schulze MB | 2005 | Diabet Med | USA | 1 | 0 | qua 4 vs 1 | 5.73 | 1 | proinsulin | diabetes |
| 4037 | Bower M | 2005 | Ann Intern Med | UK | 1 | 0 | qua 4 vs 1 | 0.15 | 1 | International Prognostic Index (factors: age. tumor stage. lactate dehydrogenase level. Eastern Cooperative Oncology Group performance status. and number of extranodal sites) | death |
| 4038 | Whittemore AS | 2005 | J Urol | USA | 1 | 0 | qua 4 vs 1 | 4.40 | 1 | serum Prostate specific antigen | prostate cancer |
| 4040 | Rajpathak S | 2005 | J Am Coll Nutr | USA | 1 | 1 | qua 4 vs 1 | 0.43 | 1 | toenail selenium | prevalent diabetes |

| | | | | | | | | | | | | |
|---|---|---|---|---|---|---|---|---|---|---|---|---|
| 4041 | Bergelt C | 2005 | Cancer | Denmark | 1 | 0 | qua 4 vs 1 | 0.80 | 1 | vital exhaustion scores | cancer at all sites |
| 4043 | Schoen RE | 2005 | Gastroenterology | USA | 1 | 1 | qua 4 vs 1 | 1.70 | 1 | IGF-I | adenoma |
| 4047 | Lukanova A | 2005 | Int J Cancer | USA | 1 | 1 | qua 4 vs 1 | 1.29 | 1 | BMI | any malignancy |
| 4048 | Mannisto S | 2005 | Cancer Causes Control | Finland | 1 | 0 | qua 4 vs 1 | 0.69 | 1 | Pork, processed Meat, potatoes | breast cancer |
| 4050 | Selvin E | 2005 | Diabetes Care | USA | 1 | 0 | qua 4 vs 1 | 2.46 | 1 | HbA1C | carotid intima-media thickness |
| 4055 | Schernhammer ES | 2005 | J Natl Cancer Inst | USA | 1 | 1 | qua 4 vs 1 | 0.59 | 1 | 6-sulphatoxymelatonin | breast cancer |
| 4056 | Bergink AP | 2005 | Bone | Netherlands | 1 | 0 | qua 4 vs 1 | 2.80 | 1 | femoral neck bone mineral density | radiographic osteoarthritis of the knee |
| 4057 | Leosdottir M | 2005 | J Intern Med | Sweden | 1 | 0 | qua 4 vs 1 | 1.46 | 1 | total fat intake | cancer mortality |
| 4058 | Tsimikas S | 2005 | N Engl J Med | USA | 1 | 0 | qua 4 vs 1 | 3.12 | 1 | oxidized phospholipid:apo B-100 ratio | coronary artery disease |
| 4059 | Benner JS | 2005 | Am J Health Syst Pharm | USA | 1 | 0 | qua 4 vs 1 | 1.15 | 1 | LDL cholesterol reduction | adherence to lipid-lowering drug therapy. |
| 4060 | Wang J | 2005 | Psychol Med | Canada | 1 | 0 | qua 4 vs 1+2+3 | 2.35 | 1 | work stress | major depressive episodes |
| 4062 | Elkind MS. | 2005 | Neurology | USA | 1 | 0 | qua 4 vs 1 | 1.75 | 1 | leukocyte count | ischemic stroke |
| 4063 | Ness AR | 2005 | Heart | UK | 1 | 0 | qua 4 vs 1 | 0.40 | 1 | childhood intake of vegetables | stroke |
| 4064 | Seddon JM | 2005 | Arch Ophthalmol | USA | 1 | 1 | qua 4 vs 1 | 2.10 | 1 | CRP | progression of age-related macular degeneration |
| 4065 | Silvera SA | 2005 | Cancer Causes Control | USA | 0 | 1 | qua 4 vs 1 | 1.43 | 0 | glycemic index | pancreatic cancer |
| 4066 | Lundblad D | 2005 | J Intern Med | Sweden | 1 | 0 | qua 4 vs 1 | 19.1 | 1 | tPA activity | myocardial infarction |
| 4067 | Lim S | 2005 | Diabetes Res Clin Pract | Korea | 1 | 0 | qua 4 vs 1 | 2.36 | 1 | CRP | waist circumference |
| 4068 | Phipatanakul W | 2005 | Ann Allergy Asthma Immunol | USA | 1 | 0 | qua 4 vs 1 | 2.32 | 1 | levels of endotoxin | wheeze in the first year of life |
| 4071 | Van Guelpen B | 2005 | Stroke | Sweden | 1 | 1 | qua 4 vs 1 | 0.21 | 1 | Plasma folate | hemorrhagic stroke |
| 4073 | Berndt SI. | 2005 | Nutrition | USA | 0 | 1 | qua 4 vs 1 | 1.31 | 0 | plasma concentration of vitamin C | prostate cancer |
| 4075 | Yeh HC | 2005 | Diabetes Care | USA | 1 | 0 | qua 4 vs 1 | 1.60 | 1 | Forced vital capacity | type 2 diabetes |
| 4076 | Sato Y | 2005 | Public Health Nutr | Japan | 0 | 1 | qua 4 vs 1 | 1.13 | 0 | fruit and vegetable intake | colon cancer |
| 4077 | Loucks EB | 2005 | Psychosom Med | USA | 1 | 1 | qua 4 vs 1 | 2.29 | 1 | Social integration | plasma concentrations of fibrinogen |
| 4079 | Wattanakit K | 2005 | Atherosclerosis | USA | 1 | 0 | qua 4 vs 1 | 1.75 | 1 | triglycerides | peripheral arterial disease |
| 4081 | Kaaks R | 2005 | J Natl Cancer Inst | France | 1 | 1 | qua 4 vs 1 | 1.73 | 1 | serum concentrations of testosterone | breast cancer |

| | | | | | | | | | | | |
|---|---|---|---|---|---|---|---|---|---|---|---|
| 4082 | Sesso HD | 2005 | Cancer Epidemiol Biomarkers Prev | USA | 0 | 1 | qua 4 vs 1 | 0.93 | 0 | plasma lycopene | breast cancer |
| 4083 | Nair GV | 2005 | Chest | USA | 1 | 0 | qua 4 vs 1 | 1.47 | 1 | Pulse pressure | Myocardial infarction or Coronary heart disease death |
| 4085 | Wong TY | 2005 | Arch Intern Med | Australia | 1 | 0 | qua 4 vs 1 | 1.53 | 1 | arteriole-to-venule ratio | type 2 diabetes |
| 4088 | Garcia-Palmieri MR | 2005 | Nutr Metab Cardiovasc Dis | USA | 1 | 0 | qua 4 vs 1 | 1.38 | 1 | pulse pressure | cardiovascular mortality |
| 4089 | Wrensch M | 2005 | Am J Epidemiol | USA | 1 | 0 | qua 4 vs 1 | 0.41 | 1 | immunoglobulin G to varicella-zoster virus | adult glioma |
| 4091 | Visser M | 2005 | J Gerontol A Biol Sci Med Sci | Netherlands | 1 | 0 | qua 4 vs 1 | 1.90 | 1 | muscle area | Mobility limitations |
| 4092 | Tsubono Y | 2005 | Br J Cancer | Japan | 0 | 1 | qua 4 vs 1 | 0.92 | 0 | fruit intake | colorectal cancer |
| 4094 | Mielke MM | 2005 | Neurology | USA | 1 | 0 | qua 4 vs 1 | 0.31 | 1 | cholesterol levels | dementia |
| 4095 | Tworoger SS | 2005 | J Natl Cancer Inst | USA | 1 | 1 | qua 4 vs 1 | 1.70 | 0 | free estradiol | breast cancer |
| 4098 | Svensson AM | 2005 | Eur Heart J | Sweden | 1 | 0 | qua 4 vs 1 | 2.66 | 1 | blood glucose | all-cause mortality |
| 4099 | Lu M | 2005 | Am J Clin Nutr | USA | 1 | 0 | qua 4 vs 1 | 2.20 | 1 | linolenic acid intake | Nuclear opacity |
| 4101 | Tsushima M | 2005 | Dig Dis Sci | USA | 0 | 1 | qua 4 vs 1 | 1.33 | 0 | serum glucose | rectal cancer |
| 4102 | Tanne D | 2005 | Stroke | Israel | 1 | 0 | qua 4 vs 1 | 1.53 | 1 | skinfold thickness | stroke mortality |
| 4103 | Stattin P | 2004 | Cancer Causes Control | Sweden | 1 | 1 | qua 4 vs 1 | 1.22 | 0 | plasma enterolactone | prostate cancer |
| 4104 | De Cosmo S | 2005 | Diabetes Care | Italy | 1 | 0 | qua 4 vs 1 | 4.10 | 1 | homeostasis model assessment of insulin resistance | albumin-to-creatinine ratio |
| 4108 | Mehling WE | 2005 | J Psychosom Res | USA | 1 | 0 | qua 4 vs 1 | 2.00 | 1 | alexithymia summary scores | low back pain |
| 4109 | Margolis KL | 2005 | Arch Intern Med | USA | 1 | 0 | qua 4 vs 1 | 2.36 | 1 | WBC counts | CHD death |
| 4110 | Meier C | 2005 | J Bone Miner Res | Australia | 1 | 0 | qua 4 vs 1 | 2.8 | 1 | carboxyterminal cross-linked telopeptide of type I collagen | fracture |
| 4113 | Al Snih S | 2004 | Aging Clin Exp Res | USA | 1 | 0 | qua 4 vs 1 | 1.90 | 1 | hand grip strength | new ADL limitation |
| 4114 | Wang TJ | 2005 | Circulation | USA | 1 | 0 | qua 4 vs 1 | 1.93 | 1 | urine albumin-creatinine ratio | hypertension |
| 4115 | Rumbold AR | 2005 | Eur J Obstet Gynecol Reprod Biol | Australia | 1 | 0 | qua 4 vs 1+2+3 | 1.75 | 1 | vitamin E intake | hypertensive disorders |
| 4116 | Hoffmeister A | 2005 | Eur J Intern Med | Germany | 0 | 1 | qua 4 vs 1 | 1.30 | 0 | CRP | cardiovascular event |
| 4118 | Hedblad B | 2005 | Atherosclerosis | Sweden | 1 | 0 | qua 4 vs 1 | 2.20 | 1 | CO exposure | CVD events |
| 4120 | Kojima M | 2005 | Am J Epidemiol | Japan | 1 | 0 | qua 4 vs 1 | 0.24 | 1 | omega-3 polyunsaturated fatty acids | colorectal cancer |
| 4121 | Kragelund C | 2005 | N Engl J Med | Denmark | 1 | 0 | qua 4 vs 1 | 2.40 | 1 | NT-pro-BNP levels | death from any cause |
| 4123 | Kurl S | 2005 | Stroke | Finland | 1 | 0 | qua 4 vs 1 | 2.70 | 1 | exercise cardiac power | any stroke |
| 4124 | Kim MK | 2005 | Int J Cancer | Japan | 1 | 1 | qua 4 vs 1 | 2.06 | 1 | traditional pattern | colon cancer |

| | | | | | | | | | | |
|---|---|---|---|---|---|---|---|---|---|---|
| 4126 | Oei HH | 2005 | Circulation | Netherlands | 1 | 1 | qua 4 vs 1 | 1.97 | 1 | Lp-PLA2 activity | coronary heart disease |
| 4129 | Kado DM | 2005 | Am J Med | USA | 1 | 0 | qua 4 vs 1 | 1.60 | 1 | plasma folate | cognitive decline |
| 4132 | Gilbert PB | 2005 | J Infect Dis | USA | 1 | 1 | qua 4 vs 1 | 0.22 | 1 | antibody blocking of gp120 binding to s-D4 | infection |
| 4136 | Ray JG | 2005 | Obstet Gynecol | Canada | 1 | 0 | qua 4 vs 1 | 2.60 | 1 | maternal weight | neural tube defects |
| 4139 | Bruno G | 2005 | Diabetes Care | Italy | 1 | 1 | qua 4 vs 1 | 1.20 | 1 | fasting C-peptide | age |
| 4140 | Engstrom G | 2005 | Diabetes | Sweden | 1 | 0 | qua 4 vs 1 | 5.60 | 1 | plasma proteins | Diabetes development |
| 4141 | Montonen J | 2005 | Eur J Clin Nutr | Finland | 1 | 0 | qua 4 vs 1 | 0.69 | 1 | green vegetables | type II diabetes mellitus |
| 4142 | Schupf N | 2005 | J Am Geriatr Soc | USA | 1 | 0 | qua 4 vs 1 | 1.80 | 1 | total cholesterol. non-HDL-C. and LDL-C | all cause mortality |
| 4145 | Montonen J | 2005 | Am J Epidemiol | Finland | 1 | 0 | qua 4 vs 1 | 0.72 | 1 | prudent pattern | type 2 diabetes |
| 4146 | Taylor HA | 2005 | Am J Cardiol | USA | 1 | 0 | qua 4 vs 1 | 5.00 | 1 | serum inflammatory markers (fibrinogen and von Willebrand Factor levels) | occurrence of coronary heart diseaseevents |
| 4148 | Mahabir S | 2005 | Cancer Epidemiol Biomarkers Prev | USA | 1 | 1 | qua 4 vs 1 | 0.53 | 1 | alcohol intake | renal cell cancer |
| 4149 | Raaschou-Nielsen O | 2005 | Cancer Epidemiol Biomarkers Prev | Denmark | 1 | 1 | qua 4 vs 1 | 0.70 | 0 | 1.1-dichloro-2. 2-bis(p-chlorophenyl)ethylene concentration | breast cancer |
| 4150 | Rinaldi S | 2005 | Cancer Epidemiol Biomarkers Prev | France | 1 | 1 | qua 4 vs 1 | 1.93 | 1 | insulin-like growth factor-I (IGF-I) | breast cancer |
| 4151 | Nakanishi N | 2005 | Arch Intern Med | Japan | 1 | 0 | qua 4 vs 1 | 0.54 | 1 | daily life energy expenditure | hypertension |
| 4153 | Schulze MB | 2004 | Diabetologia | USA | 1 | 0 | qua 4 vs 1 | 1.97 | 1 | non-HDL cholesterol | CHD |
| 4154 | Mai V | 2005 | Int J Epidemiol | USA | 1 | 1 | qua 4 vs 1 | 0.62 | 1 | Recommended Foods Score (RFS) | incident lung cancer |
| 4156 | Thomson CC | 2004 | J Public Health Manag Pract | USA | 1 | 1 | qua 4 vs 1 | 0.79 | 1 | tax | experimentation |
| 4157 | Tan ZS | 2005 | Arch Neurol | USA | 1 | 0 | qua 4 vs 1+2+3 | 2.04 | 1 | femoral neck BMD | AD |
| 4160 | Shy BD | 2004 | Prehosp Emerg Care | USA | 1 | 0 | qua 4 vs 1+2+3 | 0.42 | 1 | time from patient collapse until intubation | survival |
| 4161 | van Gelder BM | 2004 | Neurology | Netherlands | 1 | 0 | qua 4 vs 1+2+3 | 2.60 | 1 | activity duration | 10-year cognitive decline |
| 4162 | Engstrom G | 2004 | J Vasc Surg | Sweden | 1 | 1 | qua 4 vs 1 | 2.90 | 1 | inflammation-sensitive plasma proteins (ISPs) | peripheral artery disease requiring treatment |
| 4164 | Schnabel R | 2005 | Eur Heart J | Germany | 1 | 0 | qua 4 vs 1 | 3.70 | 1 | Nt-proBNP | cardiovascular risk |
| 4166 | Pussinen PJ | 2005 | Eur J Cardiovasc Prev Rehabil | Finland | 1 | 1 | qua 4 vs 1 | 3.99 | 1 | antibody levels | |

| | | | | | | | | | | | |
|---|---|---|---|---|---|---|---|---|---|---|---|
| 4167 | De Michele M | 2004 | Eur J Cardiovasc Prev Rehabil | Italy | 1 | 0 | qua 4 vs 1+2+3 | 0.42 | 1 | HDL-C | carotid intima-media thickening |
| 4179 | Baumgartner KB | 2004 | Am J Epidemiol | USA | 1 | 0 | qua 4 vs 1 | 0.16 | 1 | body mass index | tumor size |
| 4181 | Krause N | 2004 | Am J Ind Med | USA | 1 | 0 | qua 4 vs 1 | 1.65 | 1 | operators reporting ergonomic problems | low back injury |
| 4184 | Chen C | 2005 | Cancer | USA | 1 | 1 | qua 4 vs 1 | 0.67 | 0 | IGF-I levels | risk of prostate carcinoma |
| 4185 | Esmaillzadeh A | 2005 | Eur J Clin Nutr | Iran | 1 | 0 | qua 4 vs 1 | 0.61 | 1 | whole-grain intake | hypertriglyceridemia |
| 4186 | Suzuki K | 2004 | Cancer Epidemiol Biomarkers Prev | Japan | 1 | 1 | qua 4 vs 1 | 3.40 | 1 | serum oxLDL | colorectal cancer |
| 4187 | Yuan JM | 2004 | Cancer Epidemiol Biomarkers Prev | USA | 1 | 1 | qua 4 vs 1 | 0.38 | 0 | alpha-carotene | gastric cancer |
| 4189 | Kitamura A | 2004 | Stroke | Japan | 1 | 0 | qua 4 vs 1 | 3.00 | 1 | maximum IMT of the common carotid artery | stroke |
| 4190 | Giltay EJ | 2004 | Arch Gen Psychiatry | Netherlands | 1 | 0 | qua 4 vs 1 | 0.55 | 1 | optimism | all-cause mortality |
| 4191 | Sweeney C | 2004 | Am J Epidemiol | USA | 1 | 1 | qua 4 vs 1 | 1.44 | 1 | BMI | breast cancer |
| 4192 | Michaud DS | 2004 | Am J Epidemiol | USA | 0 | 1 | qua 4 vs 1 | 1.13 | 0 | inorganic arsenic concentration | bladder cancer |
| 4193 | Rossi R | 2004 | J Am Coll Cardiol | Italy | 1 | 0 | qua 4 vs 1 | 5.77 | 1 | flow-mediated dilation | hypertension |
| 4194 | Hill DJ | 2004 | Pediatr Allergy Immunol | Australia | 1 | 0 | qua 4 vs 1+2+3 | 5.90 | 1 | atopic dermatitis | IgE-mediated food allergy |
| 4196 | Luchsinger JA | 2004 | Neurology | USA | 1 | 0 | qua 4 vs 1 | 1.70 | 1 | insulin | Alzheimer disease |
| 4198 | Dallaire F | 2004 | Environ Health Perspect | Canada | 1 | 1 | qua 4 vs 1 | 1.32 | 0 | exposure to PCBs | upper respiratory tract infections |
| 4200 | Vriesendorp TM | 2004 | Eur J Vasc Endovasc Surg | Netherlands | 1 | 0 | qua 4 vs 1 | 5.10 | 1 | post-operative glucose levels | post-operative infections |
| 4201 | Kobayashi M | 2004 | Nutr Cancer | Japan | 0 | 1 | qua 4 vs 1 | 1.07 | 0 | fish consumption | colon cancer |
| 4204 | Nguyen L | 2004 | J Acquir Immune Defic Syndr | USA | 1 | 0 | qua 4 vs 1+2+3 | 3.92 | 1 | HLA score | higher viral load |
| 4207 | Boekholdt SM | 2004 | Am J Med | Netherlands | 1 | 0 | qua 4 vs 1 | 1.60 | 1 | macrophage migration inhibitory factor | myocardial infarction or death due to coronary artery disease |
| 4028 | Tworoger SS | 2004 | Cancer Res | USA | 1 | 1 | qua 4 vs 1 | 1.34 | 1 | Prolactin | breast cancer |
| 4209 | Ribeiro JC | 2004 | Am J Hum Biol | Portugal | 1 | 0 | qua 4 vs 1 | 2.00 | 1 | blood pressure. cholesterol. and obesity | 2 or 3 risk factors of metabolic syndrome |
| 4210 | Rusiecki JA | 2004 | J Natl Cancer Inst | USA | 0 | 1 | qua 4 vs 1 | 0.88 | 0 | atrazine lifetime days | prostate cancer |
| 4212 | Elkins JS | 2004 | Neurology | USA | 1 | 0 | qua 4 vs 1 | 0.68 | 1 | Stroke risk | decline on the 3MS |
| 4213 | Bautista LE | 2005 | J Hum Hypertens | USA | 1 | 0 | qua 4 vs 1 | 2.30 | 1 | interleukin 6 levels | High blood pressure |
| 4215 | Weinstein AR | 2004 | JAMA | USA | 1 | 1 | qua 4 vs 1 | 0.82 | 1 | overall activity | diabetes |
| 4216 | Manjer J | 2004 | Int J Cancer | Sweden | 1 | 0 | qua 4 vs 1 | 2.02 | 1 | estrone | breast cancer |

| | | | | | | | | | | | |
|---|---|---|---|---|---|---|---|---|---|---|---|
| 4217 | English DR | 2004 | Cancer Epidemiol Biomarkers Prev | Australia | 1 | 1 | qua 4 vs 1 | 2.30 | 0 | consumption of fresh red meat | rectal cancer |
| 4219 | Reijman M | 2004 | Arthritis Rheum | Netherlands | 1 | 0 | qua 4 vs 1 | 4.20 | 1 | C-telopeptide fragments of type II collagen level | radiographic OA of the knee |
| 4220 | Dijk JM | 2004 | Stroke | Netherlands | 1 | 0 | qua 4 vs 1 | 2.10 | 1 | Arterial stiffness | ischemic stroke or TIA |
| 4221 | Duncan BB | 2004 | Diabetes | Brazil | 1 | 1 | qua 4 vs 1 | 0.18 | 1 | adiponectin levels | diabetes |
| 4223 | Saw SM | 2004 | Invest Ophthalmol Vis Sci | Singapore | 1 | 0 | qua 4 vs 1 | 2.40 | 1 | nonverbal IQ | myopia |
| 4229 | Zhang C | 2004 | Epidemiology | USA | 1 | 1 | qua 4 vs 1 | 3.10 | 1 | Plasma ascorbic acid concentrations | gestational diabetes |
| 4231 | Kahn RS | 2004 | Arch Pediatr Adolesc Med | USA | 1 | 1 | qua 4 vs 1+2+3 | 9.20 | 1 | parents mental health | high BPI-EXT score |
| 4232 | Stattin P | 2004 | J Clin Oncol | Sweden | 1 | 1 | qua 4 vs 1 | 1.67 | 1 | plasma peptide levels of IGF-I | prostate cancer |
| 4234 | Jiang R | 2004 | Diabetes Care | USA | 1 | 1 | qua 4 vs 1 | 2.34 | 1 | non-HDL cholesterol | cardiovascular disease |
| 4238 | Rutter MK | 2004 | Circulation | UK | 1 | 0 | qua 4 vs 1 | 2.20 | 1 | CRP levels | CVD events |
| 4241 | Gago-Dominguez M | 2004 | Carcinogenesis | USA | 1 | 0 | qua 4 vs 1+2+3 | 0.36 | 1 | marine n-3 fatty acids | post-menopausal breast cancer |
| 4244 | Arenillas JF | 2004 | Neurology | Spain | 1 | 0 | qua 4 vs 1 | 3.43 | 1 | Lp(a) lipoprotein levels | greater extent of intracranial large-artery occlusive disease. |
| 4245 | Sprenkle MD | 2004 | Chest | USA | 1 | 0 | qua 4 vs 1 | 5.47 | 1 | physical component summary | death |
| 4249 | Kant AK | 2004 | J Nutr | USA | 1 | 1 | qua 4 vs 1 | 0.72 | 1 | Recommended Foods and Behavior Score | all-cause mortality |
| 4250 | Maru S | 2004 | Am J Clin Nutr | Netherlands | 1 | 0 | qua 4 vs 1 | 1.40 | 1 | BMI | mortality from all causes |
| 4251 | Okamura T | 2004 | Am Heart J | Japan | 1 | 0 | qua 4 vs 1 | 2.55 | 1 | resting heart rate (HR) | cardiovascular deaths |
| 4252 | Hayashi T | 2004 | Ann Intern Med | USA | 1 | 0 | qua 4 vs 1 | 3.48 | 1 | intra-abdominal fat area | hypertension |
| 4253 | Skuladottir H | 2004 | Lung Cancer | Denmark | 1 | 0 | qua 4 vs 1 | 0.35 | 1 | intake of plant food | lung cancer |
| 4255 | Luchsinger JA | 2004 | Neurology | USA | 0 | 1 | qua 4 vs 1 | 1.30 | 0 | homocysteine | Alzheimer disease |
| 4256 | Navas-Acien A | 2004 | Circulation | USA | 1 | 1 | qua 4 vs 1 | 2.88 | 0 | Lead levels | peripheral arterial disease |
| 4257 | Page JH | 2004 | Cancer Epidemiol Biomarkers Prev | USA | 0 | 1 | qua 4 vs 1 | 0.92 | 0 | DHEA | breast cancer |
| 4260 | Boekholdt SM | 2004 | Arterioscler Thromb Vasc Biol | Netherlands | 1 | 0 | qua 4 vs 1 | 1.72 | 1 | IL-8 | future CAD |
| 4261 | Sheehan J. | 2004 | Eur J Cardiovasc Prev Rehabil | Ireland | 1 | 0 | qua 4 vs 1 | 2.14 | 1 | corrected QT dispersion | cardiac death |
| 4264 | Oh JC | 2004 | Cancer Epidemiol Biomarkers Prev | USA | 1 | 0 | qua 4 vs 1 | 9.67 | 1 | IGF-2 | endometrial cancer |

| | | | | | | | | | |
|---|---|---|---|---|---|---|---|---|---|
| 4267 | van Meurs JB | 2004 | N Engl J Med | Netherlands | 1 | 0 | qua 4 vs 1+2+3 | 1.90 | 1 homocysteine level | fracture |
| 4270 | Zhang C | 2004 | J Reprod Med | USA | 1 | 0 | qua 4 vs 1 | 12.8 | 1 daily vitamin C intake | gestational diabetes mellitus |
| 4274 | Lin Y | 2004 | Int J Cancer | Japan | 0 | 1 | qua 4 vs 1 | 2.31 | 0 IGF-I levels | death from pancreatic cancer |
| 4275 | Butler LM | 2004 | Am J Respir Crit Care Med | USA | 1 | 0 | qua 4 vs 1 | 0.61 | 1 nonstarch polysaccharides intake | chronic respiratory symptoms |
| 4276 | Gunnarsdottir I | 2004 | Eur J Clin Nutr | Iceland | 1 | 0 | qua 4 vs 1 | 0.70 | 1 birth weight | being above the 90th percentile of truncal fat |
| 4278 | Pelfrene E | 2004 | Soc Sci Med | Belgium | 1 | 1 | qua 4 vs 1 | 1.91 | 1 evel of job demands | use of benzodiazepines |
| 4279 | Wong TY | 2004 | Am J Epidemiol | Australia | 0 | 1 | qua 4 vs 1 | 0.93 | 0 retinal arteriole-to-venule ratio | all-cause mortality |
| 4280 | Kim MK | 2004 | Int J Cancer | Japan | 1 | 0 | qua 4 vs 1 | 0.56 | 1 healthy pattern | gastric cancer |
| 4283 | Blake GJ | 2004 | Arch Intern Med | USA | 1 | 1 | qua 4 vs 1+2+3 | 2.25 | 1 A(1c) levels | cardiovascular events |
| 4287 | Turner BJ | 2004 | Ann Intern Med | USA | 1 | 0 | qua 4 vs 1 | 0.79 | 1 physician visit adherence | attending the first colon study |
| 4288 | MacInnis RJ | 2004 | Cancer Epidemiol Biomarkers Prev | Australia | 1 | 0 | qua 4 vs 1 | 2.30 | 1 fat-free mass (FFM) | male colon cancer |
| 4294 | Schulze MB | 2004 | Diabetes Care | USA | 1 | 0 | qua 4 vs 1 | 2.62 | 1 CRP | cardiovascular events |
| 4297 | Anderson JP | 2004 | Cancer | USA | 1 | 1 | qua 4 vs 1 | 1.86 | 1 height | ovarian cancer |
| 4302 | Morange PE | 2004 | Circulation | France | 1 | 0 | qua 4 vs 1 | 3.04 | 1 plasma vWF levels | hard CHD |
| 4303 | Horne BD | 2004 | Ann Epidemiol | USA | 1 | 1 | qua 4 vs 1 | 1.32 | 1 geographic Socio economic status measure-residential economic status | death/MI |
| 4305 | Samelson EJ | 2004 | Am J Epidemiol | USA | 1 | 1 | qua 4 vs 1 | 0.73 | 1 metacarpal cortical area | coronary heart disease |
| 4307 | Larsson SC | 2004 | J Natl Cancer Inst | Sweden | 1 | 1 | qua 4 vs 1 | 0.67 | 0 dietary folate intake | ovarian cancer |
| 4308 | Panichi V | 2004 | Nephrol Dial Transplant | Italy | 1 | 1 | qua 4 vs 1 | 5.20 | 1 IL-6 | death from all causes |
| 4311 | Hubbard JS | 2004 | Urology | USA | 0 | 1 | qua 4 vs 1 | 2.06 | 0 waist/hip ratio | prostate cancer |
| 4313 | Rosenthal AD | 2004 | Int J Obes Relat Metab Disord | USA | 1 | 0 | qua 4 vs 1 | 2.57 | 1 body mass index | diabetes |
| 4315 | Erlinger TP | 2004 | JAMA | USA | 1 | 1 | qua 4 vs 1 | 2.55 | 1 CRP | colon cancer |
| 4317 | Keinan-Boker L | 2004 | Am J Clin Nutr | Netherlands | 0 | 1 | qua 4 vs 1 | 1.00 | 0 intakes of isoflavones | breast cancer |
| 4318 | Massy ZA | 2004 | Am J Physiol Renal Physiol | France | 1 | 0 | qua 4 vs 1+2+3 | 3.30 | 1 plasma S-nitrosothiol concentrations | cardiac events |
| 4319 | Golden SH | 2004 | Diabetes Care | USA | 1 | 0 | qua 4 vs 1 | 1.63 | 1 depressive symptoms | Diabetes development |
| 4320 | Montonen J | 2004 | Diabetes Care | Finland | 1 | 0 | qua 4 vs 1 | 0.69 | 1 Vitamin E intake | type 2 diabetes |

| | | | | | | | | | | | |
|---|---|---|---|---|---|---|---|---|---|---|---|
| 4321 | Takebayashi T | 2004 | Occup Environ Med | Japan | 1 | 0 | qua 4 vs 1 | 3.90 | 1 | six year mean TTCA | ischaemic findings |
| 4329 | Molyneux A | 2004 | Am J Epidemiol | UK | 1 | 0 | qua 4 vs 1 | 1.78 | 1 | smoking prevalence | incident smoking |
| 4331 | Church TS | 2004 | Diabetes Care | USA | 1 | 1 | qua 4 vs 1 | 4.50 | 1 | fitness | all-cause mortality |
| 4332 | Pankow JS | 2004 | Diabetes Care | USA | 1 | 0 | qua 4 vs 1 | 1.68 | 1 | free fatty acids | type 2 diabetes |
| 4334 | Wolf M | 2004 | Diabetes Care | USA | 1 | 1 | qua 4 vs 1 | 4.90 | 1 | leukocyte count | gestational diabetes mellitus |
| 4337 | Wong TY | 2004 | Diabetes | Australia | 1 | 0 | qua 4 vs 1+2+3 | 1.53 | 1 | retinal venular diameter | gross proteinuria |
| 4340 | Engstrom G | 2004 | Arterioscler Thromb Vasc Biol | Sweden | 1 | 1 | qua 4 vs 1 | 3.20 | 1 | ISPs (fibrinogen. orosomucoid. alpha1-antitrypsin. haptoglobin. and ceruloplasmin | abdominal aortic aneurysms |
| 4341 | Sesso HD | 2004 | Am J Clin Nutr | USA | 1 | 1 | qua 4 vs 1 | 0.62 | 0 | plasma lycopene | Cardiovascular disease |
| 4342 | Rosengren A | 2004 | Eur Heart J | Sweden | 1 | 0 | qua 4 vs 1 | 0.45 | 1 | social integration | coronary morbidity |
| 4351 | Lukanova A | 2004 | Int J Cancer | France | 1 | 1 | qua 4 vs 1 | 4.13 | 1 | estradiol | endometrial cancer |
| 4353 | Wannamethee SG | 2004 | Diabetologia | UK | 1 | 0 | qua 4 vs 1 | 0.66 | 1 | birthweight SD scores | HOMA insulin resistance score |
| 4354 | Kilkkinen A | 2004 | Int J Cancer | Finland | 0 | 1 | qua 4 vs 1 | 1.30 | 0 | enterolactone | breast cancer |
| 4377 | Guerra S | 2004 | Am J Respir Crit Care Med | USA | 1 | 0 | qua 4 vs 1+2+3 | 4.50 | 1 | IFNgamma production at 3 months | developing recurrent wheezing |
| 5001 | Gelber RP | 2005 | Am J Kidney Dis | USA | 1 | 0 | qui 5 vs 1 | 1.45 | 1 | BMI | chronic kidney disease |
| 5006 | Jee SH | 2004 | Am J Epidemiol | Korea | 1 | 0 | qui 5 vs 1 | 2.1 | 1 | white blood cell count | atherosclerotic cardiovascular diseases |
| 5009 | Song Y | 2004 | Am J Cardiol | USA | 0 | 1 | qui 5 vs 1 | 0.87 | 0 | magnesium intake | Cardiovascular disease |
| 5010 | Nothlings U | 2005 | J Natl Cancer Inst | USA | 1 | 0 | qui 5 vs 1 | 1.68 | 1 | daily intake of processed meat | pancreatic cancer |
| 5011 | Wright ME | 2005 | Cancer Causes Control | USA | 0 | 1 | qui 5 vs 1 | 1.15 | 0 | renal net acid excretion | bladder cancer |
| 5012 | Selvin E | 2005 | Arch Intern Med | USA | 1 | 1 | qui 5 vs 1 | 2.37 | 1 | Diabetic adults: HbA(1c) level | CHD |
| 5013 | Kurth T | 2005 | Arch Intern Med | USA | 0 | 1 | qui 5 vs 1 | 1.12 | 0 | analgesic use | hypertension |
| 5014 | Zhang X | 2005 | Arch Intern Med | USA | 1 | 0 | qui 5 vs 1 | 0.63 | 1 | soy protein intake | fracture |
| 5015 | Schulze MB | 2005 | Am J Clin Nutr | Germany | 1 | 0 | qui 5 vs 1 | 3.09 | 1 | Diet pattern. high in sugar-sweetened soft drinks. refined grains. diet soft drinks. and processed meat but low in wine. coffee. cruciferous vegetables. and yellow vegetables | diabetes |
| 5016 | Forman JP | 2005 | Hypertension | USA | 0 | 1 | qui 5 vs 1 | 0.98 | 0 | vitamin D intake | incident hypertension |

| | | | | | | | | | | | |
|---|---|---|---|---|---|---|---|---|---|---|---|
| 5020 | Mommers M | 2005 | Cancer | Netherlands | 1 | 1 | qui 5 vs 1 | 0.98 | 0 | total vegetables intake | ovarian carcinoma |
| 5021 | Zhang SM | 2005 | Cancer Epidemiol Biomarkers Prev | USA | 1 | 1 | qui 5 vs 1 | 0.81 | 1 | total folate intake | ER- tumors |
| 5022 | Bertone-Johnson ER | 2005 | Cancer Epidemiol Biomarkers Prev | USA | 0 | 1 | qui 5 vs 1 | 0.73 | 0 | plasma levels of 25-hydroxyvitamin D [25(OH)D] | breast cancer |
| 5024 | Batty GD | 2005 | Br J Psychiatry | Denmark | 1 | 0 | qui 5 vs 1 | 1.70 | 1 | early-life IQ | subsequent psychiatric disorder in adulthood |
| 5025 | Ridker PM | 2005 | JAMA | USA | 1 | 0 | qui 5 vs 1 | 1.62 | 1 | LDL-C | future cardiovascular events |
| 5029 | Tsai CJ | 2005 | Gastroenterology | USA | 1 | 0 | qui 5 vs 1 | 1.35 | 1 | dietary carbohydrate | cholecystectomy |
| 5031 | Myint PK | 2005 | Ann Epidemiol | UK | 1 | 0 | qui 5 vs 1 | 2.15 | 1 | SF-36 scores | all cause mortality |
| 5032 | Mucci LA | 2005 | Int J Cancer | USA | 0 | 1 | qui 5 vs 1 | 0.90 | 0 | acrylamide intake | colorectal cancer |
| 5033 | Shai I | 2005 | Diabetologia | USA | 1 | 1 | qui 5 vs 1 | 1.95 | 1 | lipoprotein (Lp)(a) | CHD |
| 5034 | Mozaffarian D | 2005 | J Am Coll Cardiol | USA | 1 | 1 | qui 5 vs 1 | 0.73 | 1 | long-chain n-3 fatty acid intake | CHF |
| 5037 | Bertone-Johnson ER | 2005 | Arch Intern Med | USA | 1 | 0 | qui 5 vs 1 | 0.59 | 1 | total vitamin D intake | Premenstrual syndrome |
| 5038 | Lin J | 2005 | Cancer Causes Control | USA | 1 | 1 | qui 5 vs 1 | 0.79 | 0 | fruit intake | colorectal cancer |
| 5039 | Sin DD | 2005 | Chest | Canada | 1 | 0 | qui 5 vs 1 | 3.36 | 1 | FEV1 | ischemic heart disease |
| 5043 | Larsson SC | 2005 | Gastroenterology | Sweden | 1 | 0 | qui 5 vs 1 | 0.66 | 1 | vitamin B6 intake | colorectal cancer |
| 5046 | Sesso HD | 2005 | Cancer Epidemiol Biomarkers Prev | USA | 0 | 1 | qui 5 vs 1 | 1.00 | 0 | dietary lycopene | breast cancer |
| 5048 | Heidemann C | 2005 | Diabetologia | Germany | 1 | 0 | qui 5 vs 1 | 0.27 | 1 | dietary pattern score | type 2 diabetes |
| 5049 | Tsai CJ | 2005 | Gut | USA | 1 | 0 | qui 5 vs 1 | 1.59 | 1 | carbohydrate intake | symptomatic gall stone disease |
| 5050 | Tsai CJ | 2005 | Arch Intern Med | USA | 1 | 1 | qui 5 vs 1 | 1.23 | 1 | dietary intake of trans-fatty acids | gallstone disease |
| 5051 | Lu M | 2005 | Am J Epidemiol | USA | 1 | 1 | qui 5 vs 1 | 1.10 | 0 | total fat intake | cataract extraction |
| 5053 | Vrbova L | 2005 | J Negat Results Biomed | Canada | 0 | 1 | qui 5 vs 1 | 1.03 | 0 | income | death |
| 5055 | Slinin Y | 2005 | J Am Soc Nephrol | USA | 1 | 0 | qui 5 vs 1 | 1.08 | 1 | calcium levels | cardiovascular events |
| 5056 | Michaud DS | 2005 | J Natl Cancer Inst | USA | 0 | 1 | qui 5 vs 1 | 1.32 | 0 | prudent dietary pattern | pancreatic cancer |
| 5060 | Lin J | 2005 | Am J Epidemiol | USA | 0 | 1 | qui 5 vs 1 | 1.20 | 0 | Intakes of total calcium | colorectal cancer |
| 5063 | Larsson SC | 2005 | Cancer Epidemiol Biomarkers Prev | Sweden | 1 | 1 | qui 5 vs 1 | 0.61 | 1 | dietary folate intake | colon cancer |
| 5064 | Schernhammer ES | 2005 | Cancer Epidemiol Biomarkers Prev | USA | 1 | 1 | qui 5 vs 1 | 1.00 | 0 | IGF-I | breast cancer |
| 5065 | Fung TT | 2005 | Int J Cancer | USA | 1 | 0 | qui 5 vs 1 | 1.44 | 1 | Western pattern score | breast cancer |
| 5066 | Frost L | 2005 | Am J Clin Nutr | Denmark | 0 | 1 | qui 5 vs 1 | 0.91 | 0 | caffeine consumption | atrial fibrillation or flutter |

| | | | | | | | | | | | |
|---|---|---|---|---|---|---|---|---|---|---|---|
| 5067 | Wang Y | 2005 | Am J Clin Nutr | USA | 1 | 0 | qui 5 vs 1 | 12.0 | 1 | waist circumference | type 2 diabetes |
| 5068 | Alonso A | 2004 | Lipids | Spain | 1 | 1 | qui 5 vs 1 | 0.46 | 1 | olive oil consumption | hypertension |
| 5071 | Madsen TE | 2005 | Am J Nephrol | USA | 1 | 0 | qui 5 vs 1+2+3+4 | 1.90 | 1 | model including NAF cytology | all-cause mortality |
| 5074 | Taylor EN | 2005 | Am J Kidney Dis | USA | 1 | 0 | qui 5 vs 1 | 1.28 | 1 | docosahexaenoic acid eicosapentaenoic acid | kidney stones |
| 5076 | Hartman TJ | 2005 | J Nutr | USA | 1 | 1 | qui 5 vs 1 | 0.91 | 0 | dietary calcium intake | adenoma recurrence |
| 5077 | Michaud DS | 2005 | Cancer Epidemiol Biomarkers Prev | USA | 1 | 1 | qui 5 vs 1 | 1.32 | 0 | Dietary glycemic load | colorectal cancer |
| 5078 | Flood A | 2005 | Cancer Epidemiol Biomarkers Prev | USA | 1 | 1 | qui 5 vs 1 | 0.74 | 1 | dietary calcium | colorectal cancer |
| 5080 | Rouillier P | 2005 | Alcohol Clin Exp Res | France | 1 | 0 | qui 5 vs 1 | 1.45 | 1 | Alcohol intake | Abnormal ApoB |
| 5081 | van Gils CH | 2005 | JAMA | Netherlands | 0 | 1 | qui 5 vs 1 | 0.98 | 0 | total vegetables | breast cancer |
| 5082 | Frost L | 2005 | Am J Clin Nutr | Denmark | 1 | 0 | qui 5 vs 1 | 1.44 | 1 | n-3 fatty acids consumed from fish | atrial fibrillation or flutter |
| 5083 | Larsson SC | 2005 | JAMA | Sweden | 1 | 1 | qui 5 vs 1 | 0.59 | 1 | magnesium intake | colorectal cancer |
| 5085 | Silvera SA | 2005 | Int J Cancer | USA | 1 | 1 | qui 5 vs 1 | 0.78 | 0 | postmenopausal women: glycemic index (GI) | breast cancer |
| 5086 | Adebamowo CA | 2005 | Int J Cancer | USA | 1 | 1 | qui 5 vs 1 | 0.94 | 0 | sum of flavonols intake | breast cancer |
| 5087 | Knekt P | 2004 | Am J Clin Nutr | Finland | 1 | 1 | qui 5 vs 1 | 0.84 | 0 | dietary intake for vitamin E | Coronary heart disease |
| 5088 | Jensen MK | 2004 | Am J Clin Nutr | USA | 1 | 0 | qui 5 vs 1 | 0.82 | 1 | whole-grain intake | Coronary heart disease |
| 5089 | Taylor EN | 2004 | J Am Soc Nephrol | USA | 1 | 0 | qui 5 vs 1 | 0.69 | 1 | dietary calcium | kidney stones |
| 5090 | Wu K | 2004 | Cancer Causes Control | USA | 1 | 1 | qui 5 vs 1 | 0.84 | 0 | prudent pattern scores | colon cancer |
| 5092 | Koenig W | 2005 | Clin Chem | Germany | 1 | 0 | qui 5 vs 1 | 2.27 | 1 | cystatin C distribution | secondary Cerebrovascular disease events |
| 5093 | Fung TT | 2004 | Arch Intern Med | USA | 1 | 0 | qui 5 vs 1 | 1.49 | 1 | Western pattern | type 2 diabetes in women |
| 5094 | Khaw KT | 2004 | Am J Clin Nutr | UK | 1 | 0 | qui 5 vs 1 | 2.48 | 1 | urinary sodium | systolic blood pressure >/= 160 mm Hg |
| 5095 | Hung HC | 2004 | J Natl Cancer Inst | USA | 1 | 1 | qui 5 vs 1 | 0.95 | 0 | total fruit and vegetable intake | major chronic disease |
| 5096 | Lin J | 2004 | Am J Epidemiol | USA | 1 | 0 | qui 5 vs 1 | 1.86 | 1 | intake of fried foods | colorectal cancer |
| 5097 | Folsom AR | 2004 | Am J Epidemiol | USA | 1 | 0 | qui 5 vs 1 | 0.82 | 1 | fish intake | total mortality |
| 5098 | Wolk R | 2004 | J Am Coll Cardiol | USA | 1 | 0 | qui 5 vs 1 | 6.46 | 1 | leptin levels | future cardiovascular events |
| 5099 | Ford ES | 2004 | Eur J Clin Nutr | USA | 1 | 1 | qui 5 vs 1 | 0.68 | 1 | grain consumption | elevated C-reactive protein |

| | | | | | | | | | | | | |
|---|---|---|---|---|---|---|---|---|---|---|---|---|
| 5100 | Empana JP | 2004 | Circulation | France | 1 | 0 | qui 5 vs 1 | 2.60 | 1 | sagittal abdominal diameter | sudden death |
| 5101 | Frost L | 2004 | Arch Intern Med | Denmark | 1 | 1 | qui 5 vs 1 | 1.46 | 1 | alcohol consumption | atrial fibrillation or flutter |
| 5103 | Luo ZC | 2004 | Epidemiology | Canada | 1 | 0 | qui 5 vs 1 | 1.44 | 1 | neighborhood-income | small-for-gestational-age |
| 5106 | Tsai CJ | 2005 | Ann Intern Med | USA | 1 | 0 | qui 5 vs 1 | 0.82 | 1 | cis unsaturated fats | gallstone disease |
| 5108 | Erkkila AT | 2005 | Eur J Clin Nutr | USA | 1 | 1 | qui 5 vs 1 | 0.79 | 1 | phylloquinone intake | Coronary heart disease |
| 5113 | Hutchings A | 2004 | Med Care | UK | 1 | 0 | qui 5 vs 1 | 0.70 | 1 | socioeconomic status | hospital mortality |
| 5117 | Singh SM | 2004 | CMAJ | Canada | 1 | 0 | qui 5 vs 1 | 1.38 | 1 | Income | Colorectal investigation |
| 5118 | Boekholdt SM | 2004 | Circulation | Netherlands | 1 | 0 | qui 5 vs 1 | 1.43 | 1 | plasma levels of cholesteryl ester transfer protein | coronary artery disease |
| 5119 | Alonso A | 2004 | Br J Nutr | Spain | 1 | 1 | qui 5 vs 1 | 0.58 | 1 | vegetable consumption | undiagnosed hypertension |
| 5120 | Smith W | 2004 | Hypertension | Australia | 1 | 0 | qui 5 vs 1 | 2.60 | 1 | retinal arteriolar narrowing | severe hypertension |
| 5122 | Chen H | 2004 | Am J Epidemiol | USA | 0 | 1 | qui 5 vs 1 | 1.00 | 0 | Folate intake | Parkinson's disease |
| 5124 | Schaumberg DA | 2004 | Am J Clin Nutr | USA | 0 | 1 | qui 5 vs 1 | 0.95 | 0 | dietary glycemic load | cataract extraction |
| 5125 | Schulze MB | 2004 | Am J Clin Nutr | USA | 1 | 1 | qui 5 vs 1 | 1.59 | 1 | glycemic index | diabetes |
| 5127 | Leung H | 2004 | J Hypertens | Australia | 1 | 0 | qui 5 vs 1 | 2.40 | 1 | current DBP | Generalized arteriolar narrowing |
| 5129 | Oh K | 2004 | Cancer Epidemiol Biomarkers Prev | USA | 0 | 1 | qui 5 vs 1 | 1.11 | 0 | glycemic index | colorectal adenoma |
| 5130 | Cho E | 2004 | J Natl Cancer Inst | USA | 1 | 1 | qui 5 vs 1 | 0.86 | 1 | milk intake | colorectal cancer |
| 5131 | Tsai CJ | 2004 | Am J Gastroenterol | USA | 1 | 1 | qui 5 vs 1 | 0.87 | 1 | total dietary fiber intake | choleystectomy |
| 5132 | Fung TT | 2004 | Stroke | USA | 1 | 1 | qui 5 vs 1 | 1.58 | 1 | Western pattern | total strokes |
| 5133 | Wright ME | 2004 | Am J Epidemiol | USA | 1 | 1 | qui 5 vs 1 | 0.84 | 1 | Antioxidant diet index | Lung cancer |
| 5134 | Tsai CJ | 2004 | Am J Epidemiol | USA | 1 | 1 | qui 5 vs 1 | 1.00 | 0 | dietary total protein intake | cholecystectomy |
| 5135 | Zeleniuch-Jacquotte A | 2004 | Br J Cancer | USA | 0 | 1 | qui 5 vs 1 | 1.70 | 0 | enterolactone | breast cancer |
| 5138 | Leitzmann MF | 2004 | Am J Clin Nutr | USA | 1 | 1 | qui 5 vs 1 | 2.02 | 1 | ALA from nonanimal sources | prostate cancer |
| 5141 | Hirvonen T | 2004 | Eur J Epidemiol | Finland | 1 | 1 | qui 5 vs 1 | 0.86 | 1 | Dietary intake of flavonols and flavones | intermittent claudication |
| 5143 | Hannan LM | 2004 | Cancer Epidemiol Biomarkers Prev | USA | 1 | 1 | qui 5 vs 1 | 0.70 | 0 | total physical activity | ovarian cancer |
| 5144 | Stack AG | 2004 | Kidney Int | USA | 1 | 0 | qui 5 vs 1 | 1.44 | 1 | BMI | death |
| 5145 | Greisenegger S | 2004 | Stroke | Austria | 1 | 0 | qui 5 vs 1 | 2.60 | 1 | mean platelet volume | severe stroke |
| 5149 | Kang JH | 2004 | Am J Clin Nutr | USA | 1 | 1 | qui 5 vs 1 | 0.90 | 0 | total fat | primary open-angle glaucoma |

| | | | | | | | | | | | |
|---|---|---|---|---|---|---|---|---|---|---|---|
| 5151 | Richardson DB | 2004 | Arch Pediatr Adolesc Med | USA | 1 | 1 | qui 5 vs 1 | 2.15 | 1 | adult hemoglobin level | sudden infant death syndrome |
| 5153 | Holmes MD | 2004 | Am J Epidemiol | USA | 0 | 1 | qui 5 vs 1 | 0.97 | 0 | carbohydrate intake | breast cancer |
| 5154 | Yang G | 2004 | Eur J Clin Nutr | USA | 1 | 1 | qui 5 vs 1 | 0.51 | 1 | intake of tofu and other soy products | glycosuria |
| 5155 | Klein R | 2004 | Am J Ophthalmol | USA | 1 | 0 | qui 5 vs 1 | 1.93 | 1 | generalized retinal arteriolar narrowing | retinal pigment epithedepigmentation |
| 5157 | Chen H | 2004 | Am J Epidemiol | USA | 1 | 1 | qui 5 vs 1 | 1.90 | 1 | waist circumference | Parkinson's disease |
| 5158 | Hu FB | 2004 | Diabetes | USA | 1 | 0 | qui 5 vs 1 | 1.64 | 1 | TNF-alphaR2 | type 2 diabetes |
| 5160 | Wong TY | 2004 | Ann Intern Med | Australia | 1 | 0 | qui 5 vs 1 | 1.62 | 1 | arteriole-to-venule ratios | hypertension |
| 5162 | Al-Delaimy WK | 2004 | J Am Coll Nutr | USA | 1 | 1 | qui 5 vs 1 | 0.73 | 1 | total magnesium intake | Coronary heart disease |
| 5163 | Jiang R | 2004 | JAMA | USA | 1 | 1 | qui 5 vs 1 | 2.68 | 1 | Ferritin | Type 2 diabetes |
| 5165 | Zylberstein DE | 2004 | Circulation | Sweden | 1 | 0 | qui 5 vs 1+2+3+4 | 1.86 | 1 | Total Homocysteine | acut myocardial infarction |
| 5166 | Pell JP | 2004 | Am J Epidemiol | UK | 1 | 0 | qui 5 vs 1+2+3+4 | 1.29 | 1 | Birth weight | Maternal cerebrovascular events |
| 5167 | Higginbotham S | 2004 | J Natl Cancer Inst | USA | 1 | 1 | qui 5 vs 1 | 2.85 | 1 | dietary glycemic load | colorectal cancer |
| 5169 | McKeown NM | 2004 | Diabetes Care | USA | 1 | 0 | qui 5 vs 1 | 0.62 | 1 | cereal fiber | metabolic syndrome |
| 5171 | Higginbotham S | 2004 | Cancer Epidemiol Biomarkers Prev | USA | 1 | 1 | qui 5 vs 1 | 1.01 | 0 | glycemic load | breast cancer risk |
| 5172 | Mannisto S | 2004 | Cancer Epidemiol Biomarkers Prev | USA | 1 | 1 | qui 5 vs 1 | 0.98 | 0 | beta-Carotene intake | lung cancer |
| 5174 | Munger KL | 2004 | Neurology | USA | 1 | 1 | qui 5 vs 1 | 0.67 | 0 | total vitamin D intake | multiple sclerosis |
| 5177 | Zeleniuch-Jacquotte A | 2004 | Br J Cancer | USA | 1 | 1 | qui 5 vs 1 | 2.49 | 1 | oestradiol | breast cancer |
| 5178 | Mattisson I | 2004 | Br J Cancer | Sweden | 1 | 0 | qui 5 vs 1 | 0.58 | 1 | fibre intakes | breast cancer |
| 5180 | Song Y | 2004 | Diabetes Care | USA | 1 | 0 | qui 5 vs 1 | 0.89 | 1 | Magnesium intake | Type 2 diabetes |
| 5186 | Jiang R | 2004 | Am J Clin Nutr | USA | 1 | 1 | qui 5 vs 1 | 1.28 | 1 | heme iron from red meat | type 2 diabetes |
| 5188 | He K | 2004 | Stroke | USA | 1 | 0 | qui 5 vs 1 | 0.71 | 1 | intake of folate | ischemic stroke |
| 5191 | Stattin P | 2004 | Int J Cancer | Sweden | 0 | 1 | qui 5 vs 1 | 0.80 | 0 | total testosterone | prostate cancer |
| 5193 | Miller AB | 2004 | Int J Cancer | Germany | 1 | 0 | qui 5 vs 1 | 0.60 | 1 | fruit consumption | lung cancer |
| 5194 | Lukanova A | 2004 | Int J Cancer | France | 1 | 1 | qui 5 vs 1 | 4.76 | 1 | C-peptide | Endometrial cancer |
| 3003 | Sornay-Rendu | 2005 | J Bone Miner Res | France | 1 | 0 | ter 3 vs 1+2 | 1.45 | 1 | bone loss | fractures |
| 3004 | Pell JP | 2005 | Eur Heart J | UK | 1 | 0 | ter 3 vs 1 | 0.60 | 1 | year of arrest | death after cardiopulmonary arrest |
| 3006 | De Sutter J | 2005 | Eur Heart J | Belgium | 1 | 0 | ter 3 vs 1 | 3.24 | 1 | NT-pro-BNP | coronary events |

| | | | | | | | | | | | |
|---|---|---|---|---|---|---|---|---|---|---|---|
| 3007 | Golden SH | 2005 | Psychoneuroendocrinology | USA | 1 | 1 | ter 3 vs 1 | 1.34 | 1 | trait anger temperament scores | T2DM |
| 3008 | Eisner MD | 2005 | Thorax | USA | 1 | 0 | ter 3 vs 1 | 3.61 | 1 | Nicotine exposure | hospital admission due to asthma |
| 3013 | Harville EW | 2005 | J Womens Health (Larchmt) | USA | 0 | 1 | ter 3 vs 1 | 1.40 | 0 | overall stress | Bacterial vaginosis |
| 3014 | Khan MM. | 2005 | Int J Environ Health Res | Japan | 1 | 0 | ter 3 vs 1 | 2.12 | 1 | GOT | longer duration of having arsenicosis |
| 3015 | Touvier M | 2005 | J Natl Cancer Inst | France | 1 | 1 | ter 3 vs 1 | 0.80 | 0 | dietary intake of beta carotene | all-smoking related cancers |
| 3017 | Yende S | 2005 | Am J Respir Crit Care Med | USA | 1 | 0 | ter 3 vs 1 | 1.60 | 1 | TNF | Hospitalization due to pneumonia |
| 3018 | Koren-Morag N | 2005 | Am J Med | Israel | 1 | 0 | ter 3 vs 1 | 1.55 | 1 | WBC | ischemic cerebrovascular disease |
| 3019 | Durairaj L | 2005 | Chest | USA | 1 | 1 | ter 3 vs 1 | 0.68 | 1 | hospital volume | mortality in GI diagnoses |
| 3020 | Kawamoto R | 2005 | Intern Med | Japan | 1 | 0 | ter 3 vs 1 | 1.66 | 1 | levels of serum uric acid | intima-media thickness |
| 3023 | Tarnow L | 2005 | Scand J Clin Lab Invest Suppl | Denmark | 1 | 1 | ter 3 vs 1 | 2.64 | 1 | PIGF | cardiovascular morbidity |
| 3024 | Schuiling WJ. | 2005 | Neurosurgery | Netherlands | 1 | 0 | ter 3 vs 1 | 30.9 | 1 | acute physiology score II in subaracnoid hemmorhage patients | poor outcome after subarachnoid hemorrhage |
| 3025 | Pena de la Vega L | 2005 | Mayo Clin Proc | USA | 1 | 0 | ter 3 vs 1+2 | 2.47 | 1 | PF4-heparin antibody | all cause mortality |
| 3032 | Van Den Brink CL | 2005 | Disabil Rehabil | Netherlands | 1 | 1 | ter 3 vs 1 | 0.46 | 1 | total physical activity | disability |
| 3033 | Storti KL | 2005 | Prev Med | USA | 1 | 0 | ter 3 vs 1 | 1.59 | 1 | physical activity | development of gallstone disease |
| 3034 | Wark PA | 2005 | Cancer Epidemiol Biomarkers Prev | Netherlands | 1 | 1 | ter 3 vs 1 | 0.46 | 1 | Fruit consumption | hMLH1 protein-deficient colon cancer |
| 3036 | van der A DL | 2005 | Stroke | Netherlands | 1 | 1 | ter 3 vs 1 | 1.45 | 0 | ferritin levels | stroke |
| 3038 | Rashidkhani B | 2005 | J Nutr | Sweden | 1 | 1 | ter 3 vs 1 | 0.81 | 0 | healthy pattern diet | renal cell carcinoma |
| 3039 | Boyapati SM | 2005 | Breast Cancer Res Treat | USA | 0 | 1 | ter 3 vs 1 | 0.99 | 0 | soy intake | disease-free breast cancer survival |
| 3041 | Fujiwara Y | 2005 | J Gerontol A Biol Sci Med Sci | Japan | 1 | 0 | ter 3 vs 1+2 | 4.70 | 1 | Pulse pressure | Poor cognitive function |
| 3042 | Giles LC | 2005 | J Epidemiol Community Health | Australia | 1 | 0 | ter 3 vs 1 | 0.78 | 1 | friends network | survival time |
| 3045 | Chiu CJ | 2005 | Am J Clin Nutr | USA | 1 | 0 | ter 3 vs 1 | 2.46 | 1 | carbohydrate intake | cortical opacities |
| 3047 | Tripepi G | 2005 | J Am Soc Nephrol | Italy | 1 | 0 | ter 3 vs 1 | 2.5 | 1 | serum IL-6 | all cause mortality |
| 3048 | van Hecke MV | 2005 | Diabetologia | Netherlands | 1 | 0 | ter 3 vs 1 | 2.2 | 1 | inflammatory dysfunction | retinopathy |

| | | | | | | | | | | | |
|---|---|---|---|---|---|---|---|---|---|---|---|
| 3049 | Mangtani P | 2005 | Prev Med | UK | 1 | 0 | ter 3 vs 1 | 0.87 | 1 | deprivation | influenza vaccine uptake |
| 3054 | Djousse L | 2005 | J Am Coll Cardiol | USA | 1 | 1 | ter 3 vs 1 | 0.59 | 1 | linolenic acid | rate-adjustd QT interval+O97 |
| 3055 | Tseng M | 2005 | Am J Clin Nutr | USA | 1 | 1 | ter 3 vs 1 | 2.20 | 1 | dairy food intake | prostate cancer |
| 3056 | Haumer M | 2005 | J Vasc Surg | Austria | 1 | 0 | ter 3 vs 1 | 1.83 | 1 | neutrophil counts | all major cardiovascular events |
| 3060 | Singh JA | 2005 | Semin Arthritis Rheum | USA | 1 | 0 | ter 3 vs 1 | 1.49 | 1 | physical component summary scale | any hospitalization |
| 3065 | Salvarani C | 2005 | Arthritis Rheum | Italy | 1 | 1 | ter 3 vs 1 | 0.11 | 1 | levels of ESR | development of permanent visual loss |
| 3066 | Mallamaci F | 2005 | Am J Kidney Dis | Italy | 1 | 0 | ter 3 vs 1 | 1.72 | 1 | homocysteine levels | VA thrombosis |
| 3067 | Yamagata H | 2005 | Diabetes Care | Japan | 1 | 0 | ter 3 vs 1 | 3.10 | 1 | fasting plama glucose levels | gastric cancer |
| 3070 | Wakai K | 2005 | Nutr Cancer | Japan | 1 | 1 | ter 3 vs 1 | 0.34 | 1 | serum carotenoids. retinol. and tocopherols | colorectal cancer |
| 3071 | Cherubini A | 2005 | Neurobiol Aging | Italy | 1 | 0 | ter 3 vs 1 | 2.60 | 1 | plasma vitamin E levels | dementia |
| 3072 | Weinstein SJ | 2005 | J Natl Cancer Inst | USA | 0 | 1 | ter 3 vs 1 | 0.49 | 0 | alpha-tocopherol | prostate cancer |
| 3073 | Rossi R | 2005 | Diabetes Care | Italy | 1 | 0 | ter 3 vs 1 | 5.87 | 1 | flow-mediated dialation of the branchial artery | incident T2DM |
| 3074 | Olijhoek JK | 2005 | Diabetes Care | Netherlands | 0 | 1 | ter 3 vs 1 | 0.70 | 0 | homeostasis model assessment of insulin resistance | coronary collaterals |
| 3075 | De Bacquer D | 2005 | Am J Epidemiol | Belgium | 1 | 0 | ter 3 vs 1 | 2.40 | 1 | social support scale | coronary heart disease incidence |
| 3077 | Houston DK | 2005 | Am J Clin Nutr | USA | 1 | 0 | ter 3 vs 1 | 0.60 | 1 | dairy intakes | impaired activities of daily living |
| 3078 | Kurzius-Spencer M | 2005 | Pediatr Allergy Immunol | USA | 1 | 0 | ter 3 vs 1 | 2.28 | 1 | serum IgE production | infant eczema |
| 3082 | Platek M | 2005 | Cancer Epidemiol Biomarkers Prev | USA | 0 | 1 | ter 3 vs 1 | 1.60 | 0 | serum fructosamine | breast cancer |
| 3086 | Chao A | 2005 | JAMA | USA | 1 | 0 | ter 3 vs 1 | 1.50 | 1 | consumption of processed meat | distal colon cancer |
| 3087 | St-Pierre AC | 2005 | Arterioscler Thromb Vasc Biol | Canada | 0 | 1 | ter 3 vs 1 | 0.76 | 0 | LDL-C | ischemic heart disease |
| 3088 | Ulvik A | 2004 | Cancer Epidemiol Biomarkers Prev | Norway | 1 | 0 | ter 3 vs 1 | 1.32 | 1 | serum total homocysteine | colorectal cancer |
| 3089 | Tolmunen T | 2004 | Am J Clin Nutr | Finland | 1 | 0 | ter 3 vs 1 | 2.30 | 1 | serum concentrations of total homocysteine | depression |
| 3090 | Wang AY | 2004 | J Am Soc Nephrol | Hong Kong | 1 | 0 | ter 3 vs 1 | 4.19 | 1 | resting energy expenditure | all-cause mortality |
| 3093 | Schillinger | 2004 | Thromb Haemost | Austria | 1 | 0 | ter 3 vs 1+2 | 0.39 | 1 | Heparin cofactor II activity | Instent restenosis |
| 3094 | Zeleniuch-Jacquotte A | 2005 | Int J Cancer | USA | 0 | 1 | ter 3 vs 1 | 1.10 | 0 | levels of estradiol | breast carcinoma in situ |

| | | | | | | | | | | | |
|---|---|---|---|---|---|---|---|---|---|---|---|
| 3096 | Louis GM | 2005 | Hum Reprod | USA | 1 | 0 | ter 3 vs 1 | 3.77 | 1 | anti-estrogenic polychlorinated biphenyl | endometriosis |
| 3102 | Qiu C | 2004 | Epidemiology | Sweden | 1 | 1 | ter 3 vs 1 | 2.40 | 0 | Extremely-low-frequency magnetic fied lifetime average exposure. | Alzheimer's disease |
| 3106 | Ramos MI | 2004 | Am J Clin Nutr | USA | 1 | 0 | ter 3 vs 1 | 2.04 | 1 | folate status | Center for Epidemiologic Studies Depression Scale (CES-D) score >/=16 |
| 3110 | Qiu C | 2004 | Paediatr Perinat Epidemiol | USA | 1 | 0 | ter 3 vs 1 | 3.50 | 1 | CRP | gestational diabetes |
| 3111 | Jowett SL | 2004 | Gut | UK | 1 | 0 | ter 3 vs 1 | 3.20 | 1 | Consumption of meat | relapse of ulcerative colitis |
| 3112 | Micheli A | 2004 | Int J Cancer | Italy | 1 | 1 | ter 3 vs 1 | 2.85 | 1 | free testosterone levels | breast cancer |
| 3113 | Rajala U | 2005 | J Hum Hypertens | Finland | 1 | 0 | ter 3 vs 1+2 | 3.10 | 1 | systolic BP | systolic BP |
| 3115 | Ninomiya T | 2004 | Am J Kidney Dis | Japan | 1 | 1 | ter 3 vs 1 | 2.09 | 0 | total Homocysteine levels | chronic kidney disease |
| 3116 | Nagel G | 2005 | Eur J Clin Nutr | Germany | 1 | 0 | ter 3 vs 1 | 1.73 | 1 | margarine intake | asthma |
| 3120 | Voutilainen S | 2004 | Am J Clin Nutr | Finland | 1 | 1 | ter 3 vs 1 | 0.35 | 1 | serum folate concentrations | acute coronary events |
| 3121 | Cohen S | 2004 | Psychosom Med | USA | 1 | 0 | ter 3 vs 1 | 3.70 | 1 | childhood years during which their parents owned their home | susceptibility to colds |
| 3122 | Borugian MJ | 2004 | Cancer Epidemiol Biomarkers Prev | Canada | 1 | 1 | ter 3 vs 1 | 1.90 | 0 | levels of insulin | survival after breast cancer diagnosis |
| 3123 | Goderie-Plomp HW | 2004 | J Clin Endocrinol Metab | Netherlands | 1 | 0 | ter 3 vs 1 | 2.10 | 1 | serum estradiol | vertebral fractures |
| 3125 | Jansen MC | 2004 | Nutr Cancer | Netherlands | 1 | 0 | ter 3 vs 1+2 | 0.64 | 1 | Variety in vegetable intake | Total cancers |
| 3128 | Olivot JM | 2004 | Stroke | France | 1 | 0 | ter 3 vs 1 | 0.56 | 1 | Soluble TM concentration | brain infarctions |
| 3131 | Nagata C | 2004 | Stroke | Japan | 1 | 1 | ter 3 vs 1 | 2.33 | 1 | sodium intake | death from total stroke |
| 3134 | Kiechl S | 2004 | Circulation | Austria | 1 | 0 | ter 3 vs 1 | 2.20 | 1 | level of osteoprotegerin | cardiovascular disease |
| 3135 | Cicuttini FM | 2004 | Ann Rheum Dis | Australia | 1 | 0 | ter 3 vs 1 | 7.10 | 1 | tibial cartilage loss | knee replacement |
| 3140 | Sieri S | 2004 | Cancer Epidemiol Biomarkers Prev | Italy | 1 | 0 | ter 3 vs 1 | 0.66 | 1 | salad vegetables pattern | breast cancer |
| 3142 | Zhang X | 2004 | Int J Obes Relat Metab Disord | USA | 1 | 1 | ter 3 vs 1 | 9.00 | 1 | Body mass index | CHD |
| 3143 | Hadjadj S | 2004 | Diabetes Metab | France | 1 | 1 | ter 3 vs 1+2 | 2.01 | 1 | triglycerIde levels | progression of diabetic nephropathy |
| 3144 | Stolzenberg-Solomon RZ | 2004 | Cancer Epidemiol Biomarkers Prev | USA | 0 | 1 | ter 3 vs 1 | 0.67 | 0 | IGF-1 | pancreatic cancer |
| 3145 | Shankar A | 2004 | Am J Hypertens | USA | 1 | 0 | ter 3 vs 1 | 1.70 | 1 | WBC count | Hypertension |
| 3147 | Mishra GD | 2004 | Soc Sci Med | UK | 1 | 0 | ter 3 vs 1 | 0.79 | 1 | socioeconomic status | death |

| | | | | | | | | | | | |
|---|---|---|---|---|---|---|---|---|---|---|---|
| 3148 | Johansson L | 2004 | Stroke | Sweden | 1 | 0 | ter 3 vs 1 | 0.27 | 1 | level of VWF | intracerebral hemorrhage |
| 3149 | Ala-Mursula L | 2004 | Occup Environ Med | Finland | 1 | 0 | ter 3 vs 1 | 1.90 | 1 | worktime control | poor self rated health |
| 3150 | Haim M | 2004 | Arch Intern Med | Israel | 1 | 0 | ter 3 vs 1 | 1.47 | 1 | WBC count | total mortality |
| 3153 | Sanchez PL | 2004 | Heart | Spain | 1 | 0 | ter 3 vs 1 | 4.51 | 1 | C reactive protein | cardiovascular death |
| 3154 | Ballantyne CM | 2004 | Circulation | USA | 1 | 0 | ter 3 vs 1 | 1.78 | 1 | Lp-PLA2 | incident CHD |
| 3157 | Tseng M | 2004 | Cancer Epidemiol Biomarkers Prev | USA | 0 | 1 | ter 3 vs 1 | 0.60 | 0 | Southern pattern diet | prostate cancer |
| 3160 | Merlino LA | 2004 | Arthritis Rheum | USA | 1 | 1 | ter 3 vs 1 | 0.67 | 1 | vitamin D intake | rheumatoid arthritis |
| 3162 | Meyerhardt JA | 2004 | J Clin Oncol | USA | 1 | 1 | ter 3 vs 1 | 1.94 | 1 | hospital surgical volume | cancer recurrence |
| 2002 | Bak H | 2005 | Int J Cancer | Denmark | 1 | 1 | medians | 1.61 | 1 | DNA adduct levels | lung cancer |
| 2011 | Polychronopoulos E | 2005 | Lipids Health Dis | Greece | 1 | 0 | medians | 0.77 | 1 | Mediterranean diet | hypercholesterolemia |
| 2021 | Olson MB | 2005 | Psychosom Med | USA | 1 | 0 | medians | 1.35 | 1 | total Cook Medley Hostility Score | adverse event |
| 2038 | Verma A | 2005 | Heart | USA | 1 | 0 | medians | 2.19 | 1 | single pre-implant BNP level | ICD therapies |
| 2048 | Shalak LF | 2005 | J Perinatol | USA | 1 | 0 | medians | 2.80 | 1 | rectal temperature | admission to NICU |
| 2051 | Ceschi M | 2005 | Carcinogenesis | Switzerland | 1 | 0 | medians | 0.67 | 1 | intake level of n-6 fatty acids | breast cancer |
| 2065 | George DJ | 2005 | Clin Cancer Res | USA | 1 | 0 | medians | 1.38 | 1 | Plasma IL-6 level | hormone-refractory prostate cancer progression and patient survival |
| 2088 | Gaede P | 2005 | Diabetologia | Denmark | 1 | 0 | medians | 4.40 | 1 | plasma N-terminal probrain natriuretic peptide | cardiovascular disease |
| 2089 | Tarnow L | 2005 | Diabetologia | Denmark | 1 | 0 | medians | 3.86 | 1 | plasma N-terminal probrain natriuretic peptide | any cause death |
| 2112 | Tolmunen T | 2004 | Psychother Psychosom | Finland | 1 | 0 | medians | 3.04 | 1 | intake of folate | depression diagnosis |
| 2172 | Mayne ST | 2004 | J Am Coll Nutr | USA | 1 | 0 | medians | 0.53 | 1 | plasma lycopene | total mortality |

## References:


**1** Adebamowo CA CE. Sampson L. Katan MB. Spiegelman D. Willett WC. Holmes MD. Dietary flavonols and flavonol-rich foods intake and the risk of breast cancer. *Int J Cancer.* 2005;114:628-633.

**2** Al Snih S MK. Ottenbacher KJ. Raji MA. Hand grip strength and incident ADL disability in elderly Mexican Americans over a seven-year period. *Aging Clin Exp Res.* 2004;16:481-486.

**3** Ala-Mursula L VJ. Pentti J. Kivimaki M. Effect of employee worktime control on health: a prospective cohort study. *Occup Environ Med.* 2004;61:254-261.

**4** Al-Delaimy WK RE. Willett WC. Stampfer MJ. Hu FB. Magnesium intake and risk of coronary heart disease among men. *J Am Coll Nutr.* 2004;23:63-70.

**5** Alonso A dlFC. Martin-Arnau AM. de Irala J. Martinez JA. Martinez-Gonzalez MA. Fruit and vegetable consumption is inversely associated with blood pressure in a Mediterranean population with a high vegetable-fat intake: the Seguimiento Universidad de Navarra (SUN) Study. *Br J Nutr.* 2004;92:311-319.

**6** Alonso A M-GM. Olive oil consumption and reduced incidence of hypertension: the SUN study. *Lipids.* 2005;39:1233-1238.

**7** Anderson JP RJ. Folsom AR. Anthropometric variables. physical activity. and incidence of ovarian cancer: The Iowa Women's Health Study. *Cancer.* 2004;100:1515-1521.

**8** Arenillas JF MC. Chacon P. Rovira A. Montaner J. Coscojuela P. Sanchez E. Quintana M. Alvarez-Sabin J. High lipoprotein (a). diabetes. and the extent of symptomatic intracranial atherosclerosis. *Neurology.* 2004;63:27-32.

**9** Bak H AH. Thomsen BL. Tjonneland A. Overvad K. Vogel U. Raaschou-Nielsen O. Loft S. Bulky DNA adducts as risk indicator of lung cancer in a Danish case-cohort study. *Int J Cancer.* 2006;118:1618-1622.

**10** Ballantyne CM HR. Bang H. Coresh J. Folsom AR. Heiss G. Sharrett AR. Lipoprotein-associated phospholipase A2. high-sensitivity C-reactive protein. and risk for incident coronary heart disease in middle-aged men and women in the Atherosclerosis Risk in Communities (ARIC) study. *Circulation.* 2004;109:837-842.

**11** Batty GD ME. Osler M. Childhood IQ in relation to later psychiatric disorder: evidence from a Danish birth cohort study. *Br J Psychiatry.* 2005;187:180-181.

**12** Baumgartner KB HW. Baumgartner RN. Crumley DD. Gilliland FD. McTiernan A. Bernstein L. Ballard-Barbash R. Association of body composition and weight history with breast cancer prognostic markers: divergent pattern for Hispanic and non-Hispanic White women. *Am J Epidemiol.* 2004;160:1087-1097.

**13** Bautista LE VL. Arenas IA. Gamarra G. Independent association between inflammatory markers (C-reactive protein. interleukin-6. and TNF-alpha) and essential hypertension. *J Hum Hypertens.* 2005;19:149-154.

**14** Beddhu S RN. Pappas LM. Normalization of protein intake by body weight and the associations of protein intake with nutritional status and survival. *J Ren Nutr.* 2005;15:387-397.

**15** Benner JS PM. Smith TW. Bullano MF. Willey VJ. Williams SA. Association between short-term effectiveness of statins and long-term adherence to lipid-lowering therapy. *Am J Health Syst Pharm.* 2005;62:1468-1475.

**16** Bergelt C CJ. Prescott E. Gronbaek M. Koch U. Johansen C. Vital exhaustion and risk for cancer: a prospective cohort study on the association between depressive feelings. fatigue. and risk of cancer. *Cancer Causes Control.* 2005;104:1288-1295.

**17** Bergink AP UA. Van Leeuwen JP. Hofman A. Verhaar JA. Pols HA. Bone mineral density and vertebral fracture history are associated with incident and progressive radiographic knee osteoarthritis in elderly men and women: The Rotterdam Study. *Bone.* 2005;37:446-456.

**18** Berndt SI CH. Landis PK. Hallfrisch J. Rohrmann S. Metter EJ. Platz EA. Prediagnostic plasma vitamin C levels and the subsequent risk of prostate cancer. *Nutrition.* 2005;21:686-690.

**19** Bertone-Johnson ER CW. Holick MF. Hollis BW. Colditz GA. Willett WC. Hankinson SE. Plasma 25-hydroxyvitamin D and 1.25-dihydroxyvitamin D and risk of breast cancer. *Cancer Epidemiol Biomarkers Prev.* 2005;14:1991-1997.

**20** Bertone-Johnson ER HS. Bendich A. Johnson SR. Willett WC. Manson JE. Calcium and vitamin D intake and risk of incident premenstrual syndrome. *Arch Intern Med.* 2005;165(11):1246-1252.

**21** Blake GJ PA. Manson JE. Williams GR. Buring J. Ridker PM. Glynn RJ. Hemoglobin A1c level and future cardiovascular events among women. *Arch Intern Med.* 2004;164:757-761.

**22** Boekholdt SM KJ. Wareham NJ. Peters RJ. Jukema JW. Luben R. Bingham SA. Day NE. Kastelein JJ. Khaw KT. Plasma levels of cholesteryl ester transfer protein and the risk of future coronary artery disease in apparently healthy men and women: the prospective EPIC (European Prospective Investigation into Cancer and nutrition)-Norfolk population study. *Circulation.* 2004;110:1418-1423.

**23** Boekholdt SM PR. Day NE. Luben R. Bingham SA. Wareham NJ. Hack CE. Reitsma PH. Khaw KT. Macrophage migration inhibitory factor and the risk of myocardial infarction or death due to coronary artery disease in adults without prior myocardial infarction or stroke: the EPIC-Norfolk Prospective Population study. *Am J Med.* 2004;117:390-397.

**24** Boekholdt SM PR. Hack CE. Day NE. Luben R. Bingham SA. Wareham NJ. Reitsma PH. Khaw KT. IL-8 plasma concentrations and the risk of future coronary artery disease in apparently healthy men and women: the EPIC-Norfolk prospective population study. *Arterioscler Thromb Vasc Biol.* 2004;24:1503-1508.

**25** Borugian MJ SS. Kim-Sing C. Van Patten C. Potter JD. Dunn B. Gallagher RP. Hislop TG. Insulin. macronutrient intake. and physical activity: are potential indicators of insulin resistance associated with mortality from breast cancer? *Cancer Epidemiol Biomarkers Prev.* 2004;13:1163-1172.

**26** Bower M GB. Mandalia S. Newsom-Davis T. Thirlwell C. Dhillon T. Young AM. Powles T. Gaya A. Nelson M. Stebbing J. A prognostic index for systemic AIDS-related non-Hodgkin lymphoma treated in the era of highly active antiretroviral therapy. *Ann Intern Med.* 2005;143:265-273.

**27** Boyapati SM SX. Ruan ZX. Dai Q. Cai Q. Gao YT. Zheng W. Soyfood intake and breast cancer survival: a followup of the Shanghai Breast Cancer Study. *Breast Cancer Res Treat.* . 2005;92:11-17.

**28** Bruno G CF. Merletti F. Cavallo-Perin P. Gandolfo E. Rivetti M. Runzo C. Pinach S. Pagano G; Piedmont Study Group for Diabetes Epidemiology. Residual beta-cell function and male/female ratio are higher in incident young adults than in children: the registry of type 1 diabetes of the province of Turin. Italy. 1984-2000. *Diabetes Care.* 2005;28:312-317.

**29** Butler LM. Koh WP. Lee HP. Tseng M. Yu MC. London SJ. Prospective Study of Dietary Patterns and Persistent Cough with Phlegm among Chinese Singaporeans. *Am J Respir Crit Care Med.* Feb 1 2006;173:264-270.

**30** Butler LM KW. Lee HP. Yu MC. London SJ. Dietary fiber and reduced cough with phlegm: a cohort study in Singapore. *Am J Respir Crit Care Med.* 2004;170:279-287.

**31** Ceschi M SC. Van Den Berg D. Koh WP. Yu MC. Probst-Hensch N. The effect of cyclin D1 (CCND1) G870A-polymorphism on breast cancer risk is modified by oxidative stress among Chinese women in Singapore. *Carcinogenesis.* . 2005;26:1457-1464.

**32** Chao A TM. Connell CJ. McCullough ML. Jacobs EJ. Flanders WD. Rodriguez C. Sinha R. Calle EE. Meat consumption and risk of colorectal cancer. *JAMA.* 2005;293:172-182.

**33** Chen C LS. Voigt L. Fitzpatrick A. Plymate SR. Weiss NS. Prostate carcinoma incidence in relation to prediagnostic circulating levels of insulin-like growth factor I. insulin-like growth factor binding protein 3. and insulin. *Cancer.* 2005;103:76-84.

**34** Chen H ZS. Schwarzschild MA. Hernan MA. Logroscino G. Willett WC. Ascherio A. Folate intake and risk of Parkinson's disease. *Am J Epidemiol.* 2004;160:368-375.

**35** Chen H ZS. Schwarzschild MA. Hernan MA. Willett WC. Ascherio A. Obesity and the risk of Parkinson's disease. *Am J Epidemiol.* 2004;159:547-555.

**36** Cherubini A MA. Andres-Lacueva C. Di Iorio A. Lamponi M. Mecocci P. Bartali B. Corsi A. Senin U. Ferrucci L. Vitamin E levels. cognitive impairment and dementia in older persons: the InCHIANTI study. *Neurobiol Aging.* 2005;26:987-994.

**37** Chiu CJ MM. Rogers G. Jacques PF. Chylack LT Jr. Tung W. Hankinson SE. Willett WC. Taylor A. Carbohydrate intake and glycemic index in relation to the odds of early cortical and nuclear lens opacities. *Am J Clin Nutr.* 2005;81:1411-1416.

**38** Cho E S-WS. Spiegelman D. Beeson WL. van den Brandt PA. Colditz GA. Folsom AR. Fraser GE. Freudenheim JL. Giovannucci E. Goldbohm RA. Graham S. Miller AB. Pietinen P. Potter JD. Rohan TE. Terry P. Toniolo P. Virtanen MJ. Willett WC. Wolk A. Wu K. Yaun SS. Zeleniuch-Jacquotte A. Hunter DJ. Dairy foods. calcium. and colorectal cancer: a pooled analysis of 10 cohort studies. *J Natl Cancer Inst.* 2004;96:1015-1022.

**39** Church TS CY. Earnest CP. Barlow CE. Gibbons LW. Priest EL. Blair SN. Exercise capacity and body composition as predictors of mortality among men with diabetes. *Diabetes Care.* 2004;27:83-88.

**40** Cicuttini FM JG. Forbes A. Wluka AE. Rate of cartilage loss at two years predicts subsequent total knee arthroplasty: a prospective study. *Ann Rheum Dis.* . 2004;63:1124-1127.

**41** Cocco P FD. Billai B. D'Atri M. Melis M. Blair A. Cancer mortality among men occupationally exposed to dichlorodiphenyltrichloroethane. *Cancer Res.* 2005;65:9588-9594.

**42** Cohen S DW. Turner RB. Alper CM. Skoner DP. Childhood socioeconomic status and host resistance to infectious illness in adulthood. *Psychosom Med.* . 2004;66:553-558.

**43** Dallaire F DE. Muckle G. Vezina C. Jacobson SW. Jacobson JL. Ayotte P. Acute infections and environmental exposure to organochlorines in Inuit infants from Nunavik. *Environ Health Perspect.* 2004;112:1359-1365.

**44** De Bacquer D PE. Clays E. Mak R. Moreau M. de Smet P. Kornitzer M. De Backer G. Perceived job stress and incidence of coronary events: 3-year follow-up of the Belgian Job Stress Project cohort. *Am J Epidemiol.* 2005;161:434-441.

**45** De Cosmo S MA. Ludovico O. Mastroianno S. Di Giorgio A. Pirro L. Trischitta V. Increased urinary albumin excretion. insulin resistance. and related cardiovascular risk factors in patients with type 2 diabetes: evidence of a sex-specific association. *Diabetes Care.* 2005;28:910-915.

**46** De Michele M IA. Panico S. Celentano E. Sacchetti L. Mazzaccara C. Salvato A. Bond MG. Salvatore F. Rubba P. Effect of high-density lipoprotein cholesterol levels on carotid artery geometry in a Mediterranean female population. *Eur J Cardiovasc Prev Rehabil.* 2004;11:403-407.

**47** De Sutter J. De Bacquer D. Cuypers S. et al. Plasma N-terminal pro-brain natriuretic peptide concentration predicts coronary events in men at work: a report from the BELSTRESS study. *Eur Heart J.* Dec 2005;26(24):2644-2649.

**48** Dijk JM vdGY. Grobbee DE. Bots ML; SMART Study Group. Carotid stiffness indicates risk of ischemic stroke and TIA in patients with internal carotid artery stenosis: the SMART study. *Stroke.* 2004;35:2258-2262.

**49** Djousse L RP. Hopkins PN. Whitsel EA. Arnett DK. Eckfeldt JH. Province MA. Ellison RC; Investigators of the NHLBI Family Heart Study. Dietary linolenic acid and adjusted QT and JT intervals in the National Heart. Lung. and Blood Institute Family Heart study. *J Am Coll Cardiol.* 2005;45:1716-1722.

**50** Duncan BB SM. Pankow JS. Bang H. Couper D. Ballantyne CM. Hoogeveen RC. Heiss G. Adiponectin and the development of type 2 diabetes: the atherosclerosis risk in communities study. *Diabetes.* 2004;53:2473-2478.

**51** Durairaj L TJ. Chrischilles EA. Vaughan Sarrazin MS. Yankey J. Rosenthal GE. Hospital volume-outcome relationships among medical admissions to ICUs. *Chest.* 2005;128:1682-1689.

**52** Eisner MD KJ. Hammond SK. Koren G. Lactao G. Iribarren C. Directly measured second hand smoke exposure and asthma health outcomes. *Thorax.* 2005;60:814-821.

**53** Elkind MS SR. Boden-Albala B. Rundek T. Paik MC. Sacco RL. Relative elevation in baseline leukocyte count predicts first cerebral infarction. *Neurology.* 2005;64:2121-2125.

**54** Elkins JS OME. Longstreth WT Jr. Carlson MC. Manolio TA. Johnston SC. Stroke risk factors and loss of high cognitive function. *Neurology.* 2004;63:793-799.

**55** Empana JP DP. Charles MA. Jouven X. Sagittal abdominal diameter and risk of sudden death in asymptomatic middle-aged men: the Paris Prospective Study I. *Circulation.* . 2004;110:2781-2785.

**56** English DR MR. Hodge AM. Hopper JL. Haydon AM. Giles GG. Red meat. chicken. and fish consumption and risk of colorectal cancer. *Cancer Epidemiol Biomarkers Prev.* 2004;13:1509-1514.

**57** Engstrom G BG. Lindblad B. Janzon L. Lindgarde F. Incidence of fatal or repaired abdominal aortic aneurysm in relation to inflammation-sensitive plasma proteins. *Arterioscler Thromb Vasc Biol.* 2004;24:337-341.

**58** Engstrom G HB. Eriksson KF. Janzon L. Lindgarde F. Complement C3 is a risk factor for the development of diabetes: a population-based cohort study. *Diabetes.* 2005;54:570-575.

**59** Engstrom G S-FD. Lindblad B. Janzon L. Lindgarde F. Risk of treatment of peripheral arterial disease is related to inflammation-sensitive plasma proteins: a prospective cohort study. *J Vasc Surg.* 2004;40:1101-1105.

**60** Erkkila AT BS. Hu FB. Jacques PF. Manson JE. Rexrode KM. Stampfer MJ. Lichtenstein AH. Phylloquinone intake as a marker for coronary heart disease risk but not stroke in women. *Eur J Clin Nutr.* 2005;59(2):196-204.

**61** Erlinger TP PE. Rifai N. Helzlsouer KJ. C-reactive protein and the risk of incident colorectal cancer. *JAMA.* 2004;291:589-590.

**62** Esmaillzadeh A MP. Azizi F. Whole-grain consumption and the metabolic syndrome: a favorable association in Tehranian adults. *Eur J Clin Nutr.* 2005;59:353-362.

**63** Flood A PU. Chatterjee N. Lacey JV Jr. Schairer C. Schatzkin A. Calcium from diet and supplements is associated with reduced risk of colorectal cancer in a prospective cohort of women. *Cancer Epidemiol Biomarkers Prev.* 2005;14:126-132.

**64** Folsom AR DZ. Fish intake. marine omega-3 fatty acids. and mortality in a cohort of postmenopausal women. *Am J Epidemiol.* 2004;160:1005-1010.

**65** Ford ES MA. Liu S. Healthy Eating Index and C-reactive protein concentration: findings from the National Health and Nutrition Examination Survey III. 1988-1994. *Eur J Clin Nutr.* 2005;59:278-283.

**66** Forman JP B-FH. Willett WC. Stampfer MJ. Curhan GC. Vitamin D intake and risk of incident hypertension: results from three large prospective cohort studies. *Hypertension.* 2005;46:676-682.

**67** Fried LF. Katz R. Sarnak MJ. et al. Kidney function as a predictor of noncardiovascular mortality. *J Am Soc Nephrol.* 2005;16:3728-3735.

**68** Frost L VP. Alcohol and risk of atrial fibrillation or flutter: a cohort study. *Arch Intern Med.* 2004;164:1993-1998.

**69** Frost L VP. Caffeine and risk of atrial fibrillation or flutter: the Danish Diet. Cancer. and Health Study. *Am J Clin Nutr.* 2005;81:578-582.

**70** Frost L VP. n-3 Fatty acids consumed from fish and risk of atrial fibrillation or flutter: the Danish Diet. Cancer. and Health Study. *Am J Clin Nutr.* 2005;81:50-54.

**71** Fujiwara Y CP. Takahashi R. Amano H. Yoshida H. Kumagai S. Fujita K. Wang DG. Shinkai S. Arterial pulse wave velocity as a marker of poor cognitive function in an elderly community-dwelling population. *J Gerontol A Biol Sci Med Sci.* . 2005;60:607-612.

**72** Fung TT HF. Holmes MD. Rosner BA. Hunter DJ. Colditz GA. Willett WC. Dietary patterns and the risk of postmenopausal breast cancer. *Int J Cancer.* 2005;116:116-121.
**73** Fung TT SM. Manson JE. Willett WC. Hu FB. Dietary patterns. meat intake. and the risk of type 2 diabetes in women. *Arch Intern Med.* 2004;164:2235-2240.
**74** Fung TT SM. Manson JE. Rexrode KM. Willett WC. Hu FB. Prospective study of major dietary patterns and stroke risk in women. *Stroke. 2*004;35:2014-2019.
**75** Gaede P HP. Hess G. Parving HH. Pedersen O. Plasma N-terminal pro-brain natriuretic peptide as a major risk marker for cardiovascular disease in patients with type 2 diabetes and microalbuminuria. *Diabetologia.* 2005;48(1):156-163.
**76** Gago-Dominguez M CJ. Sun CL. Van Den Berg D. Koh WP. Lee HP. Yu MC. Marine n-3 fatty acid intake. glutathione S-transferase polymorphisms and breast cancer risk in post-menopausal Chinese women in Singapore. *Carcinogenesis.* 2004;25:2143-2147.
**77** Garcia-Palmieri MR CC. Mc Gee D. Sempos C. Smit E. Sorlie PD. Wide pulse pressure is an independent predictor of cardiovascular mortality in Puerto Rican men. *Nutr Metab Cardiovasc Dis.* 2005;15(1):71-78.
**78** Manson JE. Buring JE. Levey AS. Gaziano JM. Association between body mass index and CKD in apparently healthy men. *Am J Kidney Di.* 2005;46:871-80
**79** George DJ HS. Shepard TF. Sanford B. Vogelzang NJ. Small EJ. Kantoff PW. The prognostic significance of plasma interleukin-6 levels in patients with metastatic hormone-refractory prostate cancer: results from cancer and leukemia group B 9480. *Clin Cancer Res.* . 2005;11:1815-1820.
**80** Gilbert PB PM. Follmann D. Hudgens MG. Francis DP. Gurwith M. Heyward WL. Jobes DV. Popovic V. Self SG. Sinangil F. Burke D. Berman PW. Correlation between immunologic responses to a recombinant glycoprotein 120 vaccine and incidence of HIV-1 infection in a phase 3 HIV-1 preventive vaccine trial. *J Infect Dis.* 2005;191:666-677.
**81** Giles LC GG. Luszcz MA. Andrews GR. Effect of social networks on 10 year survival in very old Australians: the Australian longitudinal study of aging. *J Epidemiol Community Health.* . 2005;59:574-579.
**82** Giltay EJ GJ. Zitman FG. Hoekstra T. Schouten EG. Dispositional optimism and all-cause and cardiovascular mortality in a prospective cohort of elderly dutch men and women. *Arch Gen Psychiatry.* 2004;61:1126-1135.
**83** Goderie-Plomp HW vdKM. de Ronde W. Hofman A. de Jong FH. Pols HA. Endogenous sex hormones. sex hormone-binding globulin. and the risk of incident vertebral fractures in elderly men and women: the Rotterdam Study. *J Clin Endocrinol Metab.* 2004;89:3261-3269.
**84** Golden SH. Williams JE. Ford DE. et al. Anger temperament is modestly associated with the risk of type 2 diabetes mellitus: The atheroslcerosis risk in communities study. *Psychoneuroendocrinology.* 2005.
**85** Golden SH WJ. Ford DE. Yeh HC. Paton Sanford C. Nieto FJ. Brancati FL; Atherosclerosis Risk in Communities study. Depressive symptoms and the risk of type 2 diabetes: the Atherosclerosis Risk in Communities study. *Diabetes Care.* 2004;27:429-435.

**86** Greisenegger S EG. Hsieh K. Tentschert S. Mannhalter C. Lalouschek W. Is elevated mean platelet volume associated with a worse outcome in patients with acute ischemic cerebrovascular events? *Stroke.* 2004;35:1688-1691.

**87** Guerra S LI. Halonen M. Martinez FD. Wright AL. Reduced interferon gamma production and soluble CD14 levels in early life predict recurrent wheezing by 1 year of age. *Am J Respir Crit Care Med.* 2004;169:70-76.

**88** Gunnarsdottir I BB. Benediktsson R. Gudnason V. Thorsdottir I. Association between size at birth. truncal fat and obesity in adult life and its contribution to blood pressure and coronary heart disease; study in a high birth weight population. *Eur J Clin Nutr.* 2004;58:812-818.

**89** Hadjadj S D-BB. Bekherraz A. BrIdoux F. Gallois Y. Mauco G. Ebran J. Marre M. Serum triglycerides are a predictive factor for the development and the progression of renal and retinal complications in patients with type 1 diabetes. *Diabetes Metab.* . 2004;30:43-51.

**90** Haim M BV. Goldbourt U. Battler A. Behar S. Predictive value of elevated white blood cell count in patients with preexisting coronary heart disease: the Bezafibrate Infarction Prevention Study. *Arch Intern Med.* 2004;164:433-439.

**91** Hannan LM LM. Lacey JV Jr. Colbert LH. Albanes D. Schatzkin A. Schairer C. Physical activity and risk of ovarian cancer: a prospective cohort study in the United States. *Cancer Epidemiol Biomarkers Prev.* 2004;13:765-770.

**92** Hartman TJ AP. Snyder K. Slattery ML. Caan B. Paskett E. Iber F. Kikendall JW. Marshall J. Shike M. Weissfeld J. Brewer B. Schatzkin A. Lanza E; Polyp Prevention Study Group. The association of calcium and vitamin D with risk of colorectal adenomas. *J Nutr.* 2005;135:252-259.

**93** Harville EW HM. Zhang J. Perceived life stress and bacterial vaginosis. *J Womens Health (Larchmt).* . 2005;14:627-633.

**94** Haumer M AJ. Exner M. Mlekusch W. Sabeti S. Schlager O. Schwarzinger I. Wagner O. Minar E. Schillinger M. Association of neutrophils and future cardiovascular events in patients with peripheral artery disease. *J Vasc Surg.* 2005;41:610-617.

**95** Hayashi T BE. Leonetti DL. McNeely MJ. Newell-Morris L. Kahn SE. Fujimoto WY. Visceral adiposity is an independent predictor of incident hypertension in Japanese Americans. *Ann Intern Med.* 2004;140:992-1000.

**96** He K MA. Rimm EB. Rosner BA. Stampfer MJ. Willett WC. Ascherio A. Folate. vitamin B6. and B12 intakes in relation to risk of stroke among men. *Stroke.* 2004;35:169-174.

**97** Hedblad B OM. Engstrom G. Wollmer P. Janzon L. Heterogeneity of cardiovascular risk among smokers is related to degree of carbon monoxide exposure. *Atherosclerosis.* 2005;179:177-183.

**98** Heidemann C HK. Spranger J. Klipstein-Grobusch K. Mohlig M. Pfeiffer AF. Boeing H; European Prospective Investigation into Cancer and Nutrition (EPIC)--Potsdam Study Cohort. A dietary pattern protective against type 2 diabetes in the European Prospective Investigation into Cancer and Nutrition (EPIC)--Potsdam Study cohort. *Diabetologia.* 2005;48:1126-1134.

**99** Higginbotham S ZZ. Lee IM. Cook NR. Giovannucci E. Buring JE. Liu S; Women's Health Study. Dietary glycemic load and risk of colorectal cancer in the Women's Health Study. *J Natl Cancer Inst.* 2004;96:229-233.

**100** Higginbotham S ZZ. Lee IM. Cook NR. Buring JE. Liu S. Dietary glycemic load and breast cancer risk in the Women's Health Study. *Cancer Epidemiol Biomarkers Prev.* 2004;13:65-70.

**101** Hill DJ HC. Food allergy and atopic dermatitis in infancy: an epidemiologic study. *Pediatr Allergy Immunol.* 2004;15:421-427.
**102** Hirvonen T TM. Pietinen P. Korhonen P. Albanes D. Virtamo J. Flavonol and flavone intake and the risk of intermittent claudication in male smokers. *Eur J Epidemiol.* 2004;19:305-311.
**103** Hoffmeister A RD. Kunze M. Brenner H. Koenig W. Prognostic value of inflammatory markers alone and in combination with blood lipids in patients with stable coronary artery disease. *Eur J Intern Med.* 2005;16:47-52.
**104** Holmes MD LS. Hankinson SE. Colditz GA. Hunter DJ. Willett WC. Dietary carbohydrates. fiber. and breast cancer risk. *Am J Epidemiol.* 2004;159:732-9.
**105** Horne BD MJ. Lappe DL. Renlund DG. Bair TL. Bunch TJ. Anderson JL; Intermountain Heart Collaborative Study Group. Less affluent area of residence and lesser-insured status predict an increased risk of death or myocardial infarction after angiographic diagnosis of coronary disease. *Ann Epidemiol.* 2004;14:143-150.
**106** Houston DK SJ. Cai J. Haines PS. Dairy. fruit. and vegetable intakes and functional limitations and disability in a biracial cohort: the Atherosclerosis Risk in Communities Study. *Am J Clin Nutr.* . 2005;81:1454.
**107** Hu FB MJ. Li TY. Rifai N. Manson JE. Inflammatory markers and risk of developing type 2 diabetes in women. *Diabetes.* 2004;53:693-700.
**108** Hubbard JS RS. Landis PK. Metter EJ. Muller DC. Andres R. Carter HB. Platz EA. Association of prostate cancer risk with insulin. glucose. and anthropometry in the Baltimore longitudinal study of aging. *Urology.* 2004;63:253-258.
**109** Hung HC JK. Jiang R. Hu FB. Hunter D. Smith-Warner SA. Colditz GA. Rosner B. Spiegelman D. Willett WC. Fruit and vegetable intake and risk of major chronic disease. *J Natl Cancer Inst.* 2004;96:1577-1584.
**110** Hutchings A RR. Brady A. Wildman M. Rowan K. Socioeconomic status and outcome from intensive care in England and Wales. *Med Care.* 2004;42:943-951.
**111** Ito Y WK. Suzuki K. Ozasa K. Watanabe Y. Seki N. Ando M. Nishino Y. Kondo T. Ohno Y. Tamakoshi A; JACC Study Group. Lung cancer mortality and serum levels of carotenoids. retinol. tocopherols. and folic acid in men and women: a case-control study nested in the JACC Study. *J Epidemiol.* 2005;15:S140-149.
**112** Jansen MC B-d-MH. Feskens EJ. Streppel MT. Kok FJ. Kromhout D. Quantity and variety of fruit and vegetable consumption and cancer risk. *Nutr Cancer.* . 2004;48:142-148.
**113** Jee SH. Park JY. Kim HS. Lee TY. Samet JM. White blood cell count and risk for all-cause. cardiovascular. and cancer mortality in a cohort of Koreans. *Am J Epidemiol.* Dec 1 2005;162:1062-1069.
**114** Jensen MK K-BP. Hu FB. Franz M. Sampson L. Gronbaek M. Rimm EB. Intakes of whole grains. bran. and germ and the risk of coronary heart disease in men. *Am J Clin Nutr.* 2004;80:1492-1499.
**115** Jiang R MJ. Meigs JB. Ma J. Rifai N. Hu FB. Body iron stores in relation to risk of type 2 diabetes in apparently healthy women. *JAMA.* 2004;291:711-717.
**116** Jiang R MJ. Ascherio A. Stampfer MJ. Willett WC. Hu FB. Dietary iron intake and blood donations in relation to risk of type 2 diabetes in men: a prospective cohort study. *Am J Clin Nutr.* 2004;79:70-75.

**117** Jiang R SM. Li T. Rifai N. Stampfer MJ. Rimm EB. Hu FB. Non-HDL cholesterol and apolipoprotein B predict cardiovascular disease events among men with type 2 diabetes. *Diabetes Care.* 2004;27:1991-1997.

**118** Johansson L JJ. Stegmayr B. Nilsson TK. Hallmans G. Boman K. Hemostatic factors as risk markers for intracerebral hemorrhage: a prospective incident case-referent study. *Stroke.* 2004;35:826-830.

**119** Jowett SL SC. Pearce MS. Phillips E. Gregory W. Barton JR. Welfare MR. Influence of dietary factors on the clinical course of ulcerative colitis: a prospective cohort study. *GUT.* 2004;53:1479-1484.

**120** Kaaks R BF. Key T. Rinaldi S. Dossus L. Biessy C. Secreto G. Amiano P. Bingham S. Boeing H. Bueno de Mesquita HB. Chang-Claude J. Clavel-Chapelon F. Fournier A. van Gils CH. Gonzalez CA. Gurrea AB. Critselis E. Khaw KT. Krogh V. Lahmann PH. Nagel G. Olsen A. Onland-Moret NC. Overvad K. Palli D. Panico S. Peeters P. Quiros JR. Roddam A. Thiebaut A. Tjonneland A. Chirlaque MD. Trichopoulou A. Trichopoulos D. Tumino R. Vineis P. Norat T. Ferrari P. Slimani N. Riboli E. Serum sex steroids in premenopausal women and breast cancer risk within the European Prospective Investigation into Cancer and Nutrition (EPIC). *J Natl Cancer Inst.* 2005;97:755-765.

**121** Kado DM KA. Huang MH. Troen A. Rowe JW. Selhub J. Seeman TE. Homocysteine versus the vitamins folate. B6. and B12 as predictors of cognitive function and decline in older high-functioning adults: MacArthur Studies of Successful Aging. *Am J Med.* 2005;118:161-167.

**122** Kahn RS BD. Whitaker RC. Combined effect of mothers' and fathers' mental health symptoms on children's behavioral and emotional well-being. *Arch Pediatr Adolesc Med* 2004;1588(8):721-729.

**123** Kang JH PL. Willett WC. Rosner BA. Egan KM. Faberowski N. Hankinson SE. Dietary fat consumption and primary open-angle glaucoma. *Am J Clin Nutr.* 2004;79:755-764.

**124** Kant AK GB. Schatzkin A. Dietary patterns predict mortality in a national cohort: the National Health Interview Surveys. 1987 and 1992. *J Nutr.* 2004;134:1793-1799.

**125** Kawamoto R TH. Oka Y. Kodama A. Ohtsuka N. Kamitani A. Association between uric acid and carotid atherosclerosis in elderly persons. *Intern Med.* 2005;44:787-793.

**126** Keinan-Boker L vDSY. Grobbee DE. Peeters PH. Am J Clin Nutr. . *Dietary phytoestrogens and breast cancer risk.* 2004;79:282-288.

**127** Khan MM HM. Kobayashi K. Sakauchi F. Yamashita T. Ahmed MF. Hossain MD. Quamruzzaman Q. Mori M. Levels of blood and urine chemicals associated with longer duration of having arsenicosis in Bangladesh. *Int J Environ Health Res.* 2005;15:289-301.

**128** Khaw KT BS. Welch A. Luben R. O'Brien E. Wareham N. Day N. Blood pressure and urinary sodium in men and women: the Norfolk Cohort of the European Prospective Investigation into Cancer (EPIC-Norfolk). *Am J Clin Nutr.* 2004;80:1397-1403.

**129** Kiechl S SG. Wenning G. Redlich K. Oberhollenzer M. Mayr A. Santer P. Smolen J. Poewe W. Willeit J. Osteoprotegerin is a risk factor for progressive atherosclerosis and cardiovascular disease. *Circulation.* 2004;109:2175-2180.

**130** Kilkkinen A VJ. Vartiainen E. Sankila R. Virtanen MJ. Adlercreutz H. Pietinen P. Serum enterolactone concentration is not associated with breast cancer risk in a nested case-control study. *Int J Cancer.* 2004;108:277-280.
**131** Kim MK SS. Sasazuki S. Tsugane S; Japan Public Health Center-based Prospective Study Group. Prospective study of three major dietary patterns and risk of gastric cancer in Japan. *Int J Cancer.* 2004;110:435-442.
**132** Kim MK SS. Otani T. Tsugane S; Japan Public Health Center-based Prospective Study Group. Dietary patterns and subsequent colorectal cancer risk by subsite: a prospective cohort study. *Int J Cancer.* 2005;115(5):790-798
**133** Kitamura A IH. Imano H. Ohira T. Okada T. Sato S. Kiyama M. Tanigawa T. Yamagishi K. Shimamoto T. Carotid intima-media thickness and plaque characteristics as a risk factor for stroke in Japanese elderly men. *Stroke.* 2004;35:2788-2794.
**134** Klein R KB. Tomany SC. Wong TY. The relation of retinal microvascular characteristics to age-related eye disease: the Beaver Dam eye study. *Am J Ophthalmol.* 2004;137:435-444.
**135** Knekt P RJ. Pereira MA. O'Reilly EJ. Augustsson K. Fraser GE. Goldbourt U. Heitmann BL. Hallmans G. Liu S. Pietinen P. Spiegelman D. Stevens J. Virtamo J. Willett WC. Rimm EB. Ascherio A. Antioxidant vitamins and coronary heart disease risk: a pooled analysis of 9 cohorts. *Am J Clin Nutr.* 2004;80:1508-1520.
**136** Kobayashi M TY. Otani T. Hanaoka T. Sobue T. Tsugane S; JPHC Study Group. Fish. long-chain n-3 polyunsaturated fatty acids. and risk of colorectal cancer in middle-aged Japanese: the JPHC study. *Nutr Cancer.* 2004;49:32-40.
**137** Koenig W TD. Brenner H. Rothenbacher D. Plasma concentrations of cystatin C in patients with coronary heart disease and risk for secondary cardiovascular events: more than simply a marker of glomerular filtration rate. *Clin Chem.* 2005;51:321-327.
**138** Kojima M WK. Tokudome S. Suzuki K. Tamakoshi K. Watanabe Y. Kawado M. Hashimoto S. Hayakawa N. Ozasa K. Toyoshima H. Suzuki S. Ito Y. Tamakoshi A; JACC Study Group. Serum levels of polyunsaturated fatty acids and risk of colorectal cancer: a prospective study. *Am J Epidemiol.* 2005;161(5):462-471.
**139** Koren-Morag N TD. Goldbourt U. White blood cell count and the incidence of ischemic stroke in coronary heart disease patients. *Am J Med.* 2005;118:1004-1009.
**140** Koushik A HD. Spiegelman D. Anderson KE. Arslan AA. Beeson WL. van den Brandt PA. Buring JE. Cerhan JR. Colditz GA. Fraser GE. Freudenheim JL. Genkinger JM. Goldbohm RA. Hankinson SE. Koenig KL. Larsson SC. Leitzmann M. McCullough ML. Miller AB. Patel A. Rohan TE. Schatzkin A. Smit E. Willett WC. Wolk A. Zhang SM. Smith-Warner SA. Fruits and vegetables and ovarian cancer risk in a pooled analysis of 12 cohort studies. *Cancer Epidemiol Biomarkers Prev.* 2005;14:2160-2167.
**141** Kragelund C GB. Kober L. Hildebrandt P. Steffensen R. N-terminal pro-B-type natriuretic peptide and long-term mortality in stable coronary heart disease. *N Engl J Med.* 2005;352:666-675.
**142** Krause N RR. Ragland DR. Syme SL. Physical workload. ergonomic problems. and incidence of low back injury: a 7.5-year prospective study of San Francisco transit operators. *Am J Ind Med.* 2004;46:570-585.

**143** Kurl S LJ. Niskanen L. Rauramaa R. Tuomainen TP. Sivenius J. Salonen JT. Cardiac power during exercise and the risk of stroke in men. *Stroke.* 2005;36:820-824.

**144** Kurth T HC. Sturmer T. Sesso HD. Glynn RJ. Buring JE. Gaziano JM. Analgesic use and risk of subsequent hypertension in apparently healthy men. *Arch Intern Med.* 2005;165:1903-1909.

**145** Kurzius-Spencer M HM. Carla Lohman I. Martinez FD. Wright AL. Prenatal factors associated with the development of eczema in the first year of life. *Pediatr Allergy Immunol.* 2005;16:19-26.

**146** Larsson SC BL. Wolk A. Magnesium intake in relation to risk of colorectal cancer in women. *JAMA.* 2005;293:86-89.

**147** Larsson SC BL. Wolk A. High-fat dairy food and conjugated linoleic acid ntakes in relation to colorectal cancer incidence in the Swedish Mammography Cohort. *Am J Clin Nutr.* 2005;82:894-900.

**148** Larsson SC GE. Wolk A. Dietary folate intake and incidence of ovarian cancer: the Swedish Mammography Cohort. *J Natl Cancer Inst.* 2004;96:396-402.

**149** Larsson SC GE. Wolk A. Vitamin B6 intake. alcohol consumption. and colorectal cancer: a longitudinal population-based cohort of women. *Gastroenterology.* 2005;128:1830-1837.

**150** Larsson SC GE. Wolk A. A prospective study of dietary folate intake and risk of colorectal cancer: modification by caffeine intake and cigarette smoking. *Cancer Epidemiol Biomarkers Prev.* 2005;14:740-743.

**151** Leitzmann MF SM. Michaud DS. Augustsson K. Colditz GC. Willett WC. Giovannucci EL. Dietary intake of n-3 and n-6 fatty acids and the risk of prostate cancer. *Am J Clin Nutr.* 2004;80:204-216.

**152** Leosdottir M NP. Nilsson JA. Mansson H. Berglund G. Dietary fat intake and early mortality patterns-data from The Malmo Diet and Cancer Study. *J Intern Med.* 2005;258:153-165.

**153** Leung H WJ. Rochtchina E. Wong TY. Klein R. Mitchell P. Impact of current and past blood pressure on retinal arteriolar diameter in an older population. *J Hypertens.* 2004;22:1543-1549.

**154** Li G SJ. Kukull WA. Peskind ER. Tsuang DW. Breitner JC. McCormick W. Bowen JD. Teri L. Schellenberg GD. Larson EB. Serum cholesterol and risk of Alzheimer disease: a community-based cohort study. *Neurology.* 2005;65:1045-1050.

**155** Lim S LH. Kimm KC. Park C. Shin C. Cho NH. C-reactive protein level as an independent risk factor of metabolic syndrome in the Korean population CRP as risk factor of metabolic syndrome. *Diabetes Res Clin Pract.* 2005;70:126-133.

**156** Lin J ZS. Cook NR. Lee IM. Buring JE. Dietary fat and fatty acids and risk of colorectal cancer in women. *Am J Epidemiol.* 2004;160(10):1011-1022.

**157** Lin J ZS. Cook NR. Rexrode KM. Liu S. Manson JE. Lee IM. Buring JE. Dietary intakes of fruit. vegetables. and fiber. and risk of colorectal cancer in a prospective cohort of women (United States). *Cancer Causes Control.* 2005;16:225-233.

**158** Lin J ZS. Cook NR. Manson JE. Lee IM. Buring JE. Intakes of calcium and vitamin D and risk of colorectal cancer in women. *Am J Epidemiol.* 2005;161:755-764.

**159** Lin Y TA. Kikuchi S. Yagyu K. Obata Y. Ishibashi T. Kawamura T. Inaba Y. Kurosawa M. Motohashi Y. Ohno Y. Serum insulin-like growth factor-I. insulin-like growth factor binding protein-3. and the risk of pancreatic cancer death. *Int J Cancer.* 2004;110:584-588.

**160** Loucks EB BL. Gruenewald TL. Seeman TE. Social integration is associated with fibrinogen concentration in elderly men. *Psychosom Med.* 2005;67:353-358.

**161** Louis GM WJ. Whitcomb BW. Sperrazza R. Schisterman EF. Lobdell DT. Crickard K. Greizerstein H. Kostyniak PJ. Environmental PCB exposure and risk of endometriosis. *Hum Reprod.* 2004;20:279-285.

**162** Lu M CE. Taylor A. Hankinson SE. Willett WC. Jacques PF. Prospective study of dietary fat and risk of cataract extraction among US women. *Am J Epidemiol.* 2005;161:948-959.

**163** Lu M TA. Chylack LT Jr. Rogers G. Hankinson SE. Willett WC. Jacques PF. Dietary fat intake and early age-related lens opacities. *Am J Clin Nutr.* 2005;81(4):773-779.

**164** Luchsinger JA TM. Shea S. Mayeux R. Hyperinsulinemia and risk of Alzheimer disease. *Neurology.* 2004;63:1187-1192.

**165** Luchsinger JA TM. Shea S. Miller J. Green R. Mayeux R. Plasma homocysteine levels and risk of Alzheimer disease. *Neurology.* 2004;62:1972-1976.

**166** Luchtenborg M WM. de Goeij AF. Wark PA. Brink M. Roemen GM. Lentjes MH. de Bruine AP. Goldbohm RA. van 't Veer P. van den Brandt PA. Meat and Fish Consumption. APCGene Mutations and hMLH1 Expression in Colon and Rectal Cancer: a Prospective Cohort Study (The Netherlands). *Cancer Causes Control. 2005;19:*1041-54.

**167** Lukanova A. Bjor O. Kaaks R. et al. Body mass index and cancer: results from the Northern Sweden Health and Disease Cohort. *Int J Cancer.* Jan 15 2006;118:458-466.

**168** Lukanova A. Zeleniuch-Jacquotte A. Lundin E. et al. Prediagnostic levels of C-peptide. IGF-I. IGFBP -1. -2 and -3 and risk of endometrial cancer. *Int J Cancer.* Jan 10 2004;108:262-268.

**169** Lukanova A LE. Micheli A. Arslan A. Ferrari P. Rinaldi S. Krogh V. Lenner P. Shore RE. Biessy C. Muti P. Riboli E. Koenig KL. Levitz M. Stattin P. Berrino F. Hallmans G. Kaaks R. Toniolo P. Zeleniuch-Jacquotte A. Circulating levels of sex steroid hormones and risk of endometrial cancer in postmenopausal women. *Int J Cancer.* 2004;108:425-432.

**170** Lundblad D DB. Rautio A. Roder ME. Eliasson M. Low level of tissue plasminogen activator activity in non-diabetic patients with a first myocardial infarction. *J Intern Med.* 2005;258:13-20.

**171** Luo ZC KW. Wilkins R. Liston RM. Mohamed J. Kramer MS; British Columbia Vital Statistics Agency. Disparities in birth outcomes by neighborhood income: temporal trends in rural and urban areas. british columbia. *Epidemiology.* 2004;15:679-686.

**172** MacInnis RJ ED. Hopper JL. Haydon AM. Gertig DM. Giles GG. Body size and composition and colon cancer risk in men. *Cancer Epidemiol Biomarkers Prev.* 2004;13:553-559.

**173** Madsen TE MJ. Carlquist JF. Horne BD. Bair TL. Jackson JD. Lappe JM. Pearson RR. Anderson JL. Serum uric acid independently predicts mortality in

patients with significant. angiographically defined coronary disease. *Am J Nephrol.* 2005;25:45-49.
**174** Mahabir S LM. Virtanen MJ. Virtamo J. Pietinen P. Albanes D. Taylor PR. Prospective study of alcohol drinking and renal cell cancer risk in a cohort of finnish male smokers. *Cancer Epidemiol Biomarkers Prev.* 2005;14:170-175.
**175** Mai V KA. Flood A. Lacey JV Jr. Schairer C. Schatzkin A. Diet quality and subsequent cancer incidence and mortality in a prospective cohort of women. *Int J Epidemiol.* 2005;34:54-60.
**176** Mallamaci F BG. Seminara G. Rapisarda F. Fatuzzo P. Candela V. Scudo P. Spoto B. Testa A. Tripepi G. Tech S. Zoccali C. Hyperhomocysteinemia and arteriovenous fistula thrombosis in hemodialysis patients. *Am J Kidney Dis.* 2005;45:702-707.
**177** Mangtani P BE. Kovats S. Ng ES. Roberts JA. Fletcher A. Inequalities in influenza vaccine uptake among people aged over 74 years in Britain. *Prev Med.* . 2005;41:545-553.
**178** Manjer J JR. Lenner P. Smoking is associated with postmenopausal breast cancer in women with high levels of estrogens. *Int J Cancer.* 2004;112:324-328.
**179** Mannisto S DL. Balder HF. Virtanen MJ. Krogh V. Khani BR. Berrino F. van den Brandt PA. Hartman AM. Pietinen P. Tan F. Wolk A. Goldbohm RA. Dietary patterns and breast cancer risk: results from three cohort studies in the DIETSCAN project. *Cancer Causes Control.* 2005;16:725-733.
**180** Mannisto S S-WS. Spiegelman D. Albanes D. Anderson K. van den Brandt PA. Cerhan JR. Colditz G. Feskanich D. Freudenheim JL. Giovannucci E. Goldbohm RA. Graham S. Miller AB. Rohan TE. Virtamo J. Willett WC. Hunter DJ. Dietary carotenoids and risk of lung cancer in a pooled analysis of seven cohort studies. *Cancer Epidemiol Biomarkers Prev.* 2004;13(1):40-48.
**181** Margolis KL MJ. Greenland P. Rodabough RJ. Bray PF. Safford M. Grimm RH Jr. Howard BV. Assaf AR. Prentice R; Women's Health Initiative Research Group. Leukocyte count as a predictor of cardiovascular events and mortality in postmenopausal women: the Women's Health Initiative Observational Study. *Arch Intern Med.* 2005;165:500-508.
**182** Maru S vdSY. Gimbrere CH. Grobbee DE. Peeters PH. Body mass index and short-term weight change in relation to mortality in Dutch women after age 50 y. *Am J Clin Nutr.* 2004;80:231-236.
**183** Massy ZA FC. Borderie D. Tuppin P. Nguyen-Khoa T. Benoit MO. Jacquot C. Buisson C. Drueke TB. Ekindjian OG. Lacour B. Iliou MC. Increased pasma S-nitrosothiol concentrations predict cardiovascular outcomes among patients with end-stage renal disease: a prospective study. *J Am Soc Nephrol.* 2004;15:470-476.
**184** Mattisson I WE. Johansson U. Gullberg B. Olsson H. Berglund G. Intakes of plant foods. fibre and fat and risk of breast cancer--a prospective study in the Malmo Diet and Cancer cohort. *Br J Cancer.* 2004;90:122-127.
**185** Mayne ST. Cartmel B. Lin H. Zheng T. Goodwin WJ. Jr. Low plasma lycopene concentration is associated with increased mortality in a cohort of patients with prior oral. pharynx or larynx cancers. *J Am Coll Nutr.* Feb 2004;23:34-42.
**186** McKeown NM MJ. Liu S. Saltzman E. Wilson PW. Jacques PF. Carbohydrate nutrition. insulin resistance. and the prevalence of the metabolic syndrome in the Framingham Offspring Cohort. *Diabetes Care.* 2004;27:538-546.

**187** Mehling WE KN. Are difficulties perceiving and expressing emotions associated with low-back pain? The relationship between lack of emotional awareness (alexithymia) and 12-month prevalence of low-back pain in 1180 urban public transit operators. *J Psychosom Res.* 2005;58:73-81.

**188** Meier C NT. Center JR. Seibel MJ. Eisman JA. Bone resorption and osteoporotic fractures in elderly men: the dubbo osteoporosis epidemiology study. *J Bone Miner Res.* 2005;20:579-587.

**189** Merkin SS CJ. Roux AV. Taylor HA. Powe NR. Area socioeconomic status and progressive CKD: the Atherosclerosis Risk in Communities (ARIC) Study. *Am J Kidney Dis.* 2005;46:203-213.

**190** Merlino LA CJ. Mikuls TR. Cerhan JR. Criswell LA. Saag KG; Iowa Women's Health Study. Vitamin D intake is inversely associated with rheumatoid arthritis: results from the Iowa Women's Health Study. *Arthritis Rheum.* 2004;50:72-77.

**191** Messer LC. Dole N. Kaufman JS. Savitz DA. Pregnancy Intendedness. Maternal Psychosocial Factors and Preterm Birth. *Matern Child Health J.* 2005:1-10.

**192** Meyerhardt JA TJ. Niedzwiecki D. Hollis DR. Schrag D. Ayanian JZ. O'Connell MJ. Weeks JC. Mayer RJ. Willett CG. MacDonald JS. Benson AB 3rd. Fuchs CS. Impact of hospital procedure volume on surgical operation and long-term outcomes in high-risk curatively resected rectal cancer: findings from the Intergroup 0114 Study. *J Clin Oncol.* . 2004;22:166-174.

**193** Michaud DS FC. Liu S. Willett WC. Colditz GA. Giovannucci E. Dietary glycemic load. carbohydrate. sugar. and colorectal cancer risk in men and women. *Cancer Epidemiol Biomarkers Prev.* 2005;14:138-147.

**194** Michaud DS SH. Wu K. Hu F. Giovannucci E. Willett WC. Colditz GA. Fuchs CS. Dietary patterns and pancreatic cancer risk in men and women. *J Natl Cancer Inst.* 2005;97:518-524.

**195** Michaud DS WM. Cantor KP. Taylor PR. Virtamo J. Albanes D. Arsenic concentrations in prediagnostic toenails and the risk of bladder cancer in a cohort study of male smokers. *Am J Epidemiol.* 2004;160:853-859.

**196** Micheli A MP. Secreto G. Krogh V. Meneghini E. Venturelli E. Sieri S. Pala V. Berrino F. Endogenous sex hormones and subsequent breast cancer in premenopausal women. *Int J Cancer.* 2004;112:312-318.

**197** Mielke MM. Zandi PP. Sjogren M. et al. High total cholesterol levels in late life associated with a reduced risk of dementia. *Neurology.* May 24 2005;64:1689-1695.

**198** Miller AB AH. Bueno-de-Mesquita B. Boshuizen HC. Agudo A. Berrino F. Gram IT. Janson L. Linseisen J. Overvad K. Rasmuson T. Vineis P. Lukanova A. Allen N. Amiano P. Barricarte A. Berglund G. Boeing H. Clavel-Chapelon F. Day NE. Hallmans G. Lund E. Martinez C. Navarro C. Palli D. Panico S. Peeters PH. Quiros JR. Tjonneland A. Tumino R. Trichopoulou A. Trichopoulos D. Slimani N. Riboli E. Fruits and vegetables and lung cancer: Findings from the European Prospective Investigation into Cancer and Nutrition. *Int J Cancer.* 2004;108:269-272.

**199** Mishra GD BK. Dobson AJ. Byles JE. Do socioeconomic gradients in women's health widen over time and with age? *Soc Sci Med.* 2004;58:1585-1595.

**200** Molyneux A LS. Antoniak M. Browne W. McNeill A. Godfrey C. Madeley R. Britton J. Prospective study of the effect of exposure to other smokers in high

school tutor groups on the risk of incident smoking in adolescence. *Am J Epidemiol.* 2004;159:127-132.
**201** Mommers M SL. Goldbohm RA. van den Brandt PA. Consumption of vegetables and fruits and risk of ovarian carcinoma. *Cancer.* 2005;104:1512-1519.
**202** Montonen J. Knekt P. Harkanen T. et al. Dietary patterns and the incidence of type 2 diabetes. *Am J Epidemiol.* Feb 1 2005;161:219-227.
**203** Montonen J. Knekt P. Jarvinen R. Reunanen A. Dietary antioxidant intake and 7risk of type 2 diabetes. *Diabetes Care.* Feb 2004;27:362-366.
**204** Montonen J JR. Heliovaara M. Reunanen A. Aromaa A. Knekt P. Food consumption and the incidence of type II diabetes mellitus. *Eur J Clin Nutr.* 2005;59:441-448.
**205** Morange PE SC. Alessi MC. Luc G. Arveiler D. Ferrieres J. Amouyel P. Evans A. Ducimetiere P. Juhan-Vague I; PRIME Study Group. Endothelial cell markers and the risk of coronary heart disease: the Prospective Epidemiological Study of Myocardial Infarction (PRIME) study. *Circulation.* 2004;109:1343-1348Circulation.
**206** Mozaffarian D BC. Lemaitre RN. Burke GL. Siscovick DS. Fish intake and risk of incident heart failure. *J Am Coll Cardiol.* 2005;45:2015-2021.
**207** Mucci LA AH. Wolk A. Prospective study of dietary acrylamide and risk of colorectal cancer among women. *Int J Cancer.* 2005;118:169-173.
**208** Munger KL ZS. O'Reilly E. Hernan MA. Olek MJ. Willett WC. Ascherio A. Vitamin D intake and incidence of multiple sclerosis. *Neurology.* 2004;62:60-65.
**209** Myint PK. Luben RN. Surtees PG. et al. Relation Between Self-Reported Physical Functional Health and Chronic Disease Mortality in Men And Women in the European Prospective Investigation Into Cancer (EPIC-Norfolk): A Prospective Population Study. *Ann Epidemiol.* 2005.
**210** Nagata C TN. Shimizu N. Shimizu H. Sodium intake and risk of death from stroke in Japanese men and women. *Stroke.* 2004;35:1543-1547.
**211** Nagel G LJ. Dietary intake of fatty acids. antioxidants and selected food groups and asthma in adults. *Eur J Clin Nutr.* 2005;59:8-15.
**212** Nair GV CL. Vittinghoff E. Herrington DM; Heart and Estrogen/Progestin Replacement Study Investigators. Pulse pressure and cardiovascular events in postmenopausal women with coronary heart disease. *Chest.* 2005;127:1498-1506.
**213** Nakanishi N SK. Arch Intern Med. . *Daily life activity and the risk of developing hypertension in middle-aged Japanese men.* 2005;165(2):214-220.
**214** Navas-Acien A SE. Sharrett AR. Calderon-Aranda E. Silbergeld E. Guallar E. Lead. cadmium. smoking. and increased risk of peripheral arterial disease. *Circulation.* 2004;109):3196-3201.
**214** Ness AR MM. Frankel S. Smith GD. Frobisher C. Leary SD. Emmett PM. Gunnell D. Diet in childhood and adult cardiovascular and all cause mortality: the Boyd Orr cohort. *Heart.* 2005;91:894-898.
**216** Nguyen L CT. Vanichseni S. McNicholl JM. Mock PA. Nelson R. Hodge TW. van Griensven F. Choopanya K. Mastro TD. Tappero JW. Hu DJ. Frequent human leukocyte antigen class I alleles are associated with higher viral load among HIV type 1 seroconverters in Thailand. *J Acquir Immune Defic Syndr.* . 2004;37:1318-1323.

**217** Ninomiya T KY. Kubo M. Tanizaki Y. Tanaka K. Okubo K. Nakamura H. Hata J. Oishi Y. Kato I. Hirakata H. Iida M. Hyperhomocysteinemia and the development of chronic kidney disease in a general population: the Hisayama study. *Am J Kidney Dis.* . 2004;44:437-445.

**217** Nothlings U WL. Murphy SP. Hankin JH. Henderson BE. Kolonel LN. Meat and fat intake as risk factors for pancreatic cancer: the multiethnic cohort study. *J Natl Cancer Inst.* 2005;97:1458-1465.

**219** Oei HH vdMI. Hofman A. Koudstaal PJ. Stijnen T. Breteler MM. Witteman JC. Lipoprotein-associated phospholipase A2 activity is associated with risk of coronary heart disease and ischemic stroke: the Rotterdam Study. *Circulation.* 2005;111:570-575.

**220** Oh JC WW. Tortolero-Luna G. Broaddus R. Gershenson DM. Burke TW. Schmandt R. Lu KH. Increased plasma levels of insulin-like growth factor 2 and insulin-like growth factor binding protein 3 are associated with endometrial cancer risk. *Cancer Epidemiol Biomarkers Prev.* 2004;13:748-752.

**221** Oh K WW. Fuchs CS. Giovannucci EL. Glycemic index. glycemic load. and carbohydrate intake in relation to risk of distal colorectal adenoma in women. *Cancer Epidemiol Biomarkers Prev.* 2004;13:1192-1198.

**222** Okamura T HT. Kadowaki T. Kita Y. Okayama A. Elliott P. Ueshima H; NIPPONDATA80 Research Group. Resting heart rate and cause-specific death in a 16.5-year cohort study of the Japanese general population. *Am Heart J.* 2004;147:1024-1032.

**223** Olijhoek JK KJ. de Jaegere PP. Verhaar MC. Grobbee DE. van der Graaf Y. Visseren FL; SMART Study Group. Presence of the metabolic syndrome does not impair coronary collateral vessel formation in patients with documented coronary artery disease. *Diabetes Care.* 2005;28:683-689.

**224** Olivot JM LJ. Aiach M. Amarenco P; GENIC Investigators. Soluble thrombomodulin and brain infarction: case-control and prospective study. *Stroke.* 2004;35:1946-1951.

**225** Olson MB KD. Kelsey SF. Pepine CJ. Sopko G. Handberg E. Rogers WJ. Gierach GL. McClure CK. Merz CN; WISE Study Group. Hostility scores are associated with increased risk of cardiovascular events in women undergoing coronary angiography: a report from the NHLBI-Sponsored WISE Study. *Psychosom Med.* 2005;67:546-562.

**226** Page JH CG. Rifai N. Barbieri RL. Willett WC. Hankinson SE. Plasma adrenal androgens and risk of breast cancer in premenopausal women. *Cancer Epidemiol Biomarkers Prev.* 2004;13:1032-1036.

**227** Panichi V MU. Taccola D. Migliori M. Rizza GM. Consani C. Bertini A. Sposini S. Perez-Garcia R. Rindi P. Palla R. Tetta C. Interleukin-6 is a stronger predictor of total and cardiovascular mortality than C-reactive protein in haemodialysis patients. *Nephrol Dial Transplant.* 2004;19:1154-1160.

**228** Pankow JS DB. Schmidt MI. Ballantyne CM. Couper DJ. Hoogeveen RC. Golden SH; Atherosclerosis Risk in Communities Study. Fasting plasma free fatty acids and risk of type 2 diabetes: the atherosclerosis risk in communities study. *Diabetes Care.* 2004;27:77-82.

**229** Park HA LJ. Kuller LH. Relationship between premenopausal dietary intake and postmenopausal subclinical atherosclerosis. *Atherosclerosis.* 2005.

**230** Pelfrene E VP. Moreau M. Mak RP. Kornitzer M. De Backer G. Use of benzodiazepine drugs and perceived job stress in a cohort of working men and

women in Belgium. Results from the BELSTRESS-study. *Soc Sci Med.* 2004;59:433-442.

**231** Pell JP. Corstorphine M. McConnachie A. et al. Post-discharge survival following pre-hospital cardiopulmonary arrest due to cardiac aetiology: temporal trends and impact of changes in clinical management. *Eur Heart J.* Feb 2006;27:406-412.

**232** Pell JP SG. Walsh D. Pregnancy complications and subsequent maternal cerebrovascular events: a retrospective cohort study of 119.668 births. *Am J Epidemiol.* 2004;159:336-342.

**233** Pena de la Vega L MR. Benda MM. Grill DE. Johnson MG. McCarthy JT. McBane RD 2nd. Association of heparin-dependent antibodies and adverse outcomes in hemodialysis patients: a population-based study. *Mayo Clin Proc.* 2005;80:995-1000.

**234** Phipatanakul W CJ. Sredl DL. Weiss ST. Gold DR. Mouse exposure and wheeze in the first year of life. *Ann Allergy Asthma Immunol.* 2005;94:593-599.

**235** Platek M KV. Micheli A. Browne R. Meneghini E. Sieri S. Schunemann HJ. Pala V. Barba M. Wilding GE. Berrino F. Muti P. Serum fructosamine and subsequent breast cancer risk: a nested case-control study in the ORDET prospective cohort study. *Cancer Epidemiol Biomarkers Prev.* 2005;14:271-274.

**236** Polychronopoulos E PD. Polystipioti A.. Diet. lifestyle factors and hypercholesterolemia in elderly men and women from Cyprus. *Lipids Health Dis.* 2005;4:17.

**237** Pussinen PJ AG. Tuomilehto J. Asikainen S. Jousilahti P. High serum antibody levels to Porphyromonas gingivalis predict myocardial infarction. *Eur J Cardiovasc Prev Rehabil.* 2004;11:408-411.

**238** Qiu C FL. Karp A. Winblad B. Bellander T. Occupational exposure to electromagnetic fields and risk of Alzheimer's disease. *Epidemiology.* 2004;15:687-694.

**239** Qiu C ST. Luthy DA. Williams MA. A prospective study of maternal serum C-reactive protein (CRP) concentrations and risk of gestational diabetes mellitus. *Paediatr Perinat Epidemiol.* . 2004;18:377-384.

**240** Raaschou-Nielsen O PM. Leblanc A. Dumas P. Philippe Weber J. Olsen A. Tjonneland A. Overvad K. Olsen JH. Adipose organochlorine concentrations and risk of breast cancer among postmenopausal Danish women. *Cancer Epidemiol Biomarkers Prev.* 2005;14:67-74.

**241** Rajala U LM. Paivansalo M. Suramo I. Keinanen-Kiukaanniemi S. Blood pressure and atherosclerotic plaques in carotid. aortic and femoral arteries in elderly Finns with diabetes mellitus or impaired glucose tolerance. *J Hum Hypertens.* . 2005;19:85-91.

**242** Rajpathak S RE. Morris JS. Hu F. Toenail selenium and cardiovascular disease in men with diabetes. *J Am Coll Nutr.* 2005;24(4):250-256.

**243** Ramos MI AL. Haan MN. Green R. Miller JW. Plasma folate concentrations are associated with depressive symptoms in elderly Latina women despite folic acid fortification. *Am J Clin Nutr.* . 2004;80:1024-1028.

**244** Rashidkhani B AA. Lindblad P. Wolk A. Major dietary patterns and risk of renal cell carcinoma in a prospective cohort of Swedish women. *J Nutr.* . 2005;135:1757-1762.

**245** Ray JG WP. Vermeulen MJ. Meier C. Cole DE. Greater maternal weight and the ongoing risk of neural tube defects after folic acid flour fortification. *Obstet Gynecol.* 2005;105:261-265.
**246** Reijman M HJ. Bierma-Zeinstra SM. Koes BW. Christgau S. Christiansen C. Uitterlinden AG. Pols HA. A new marker for osteoarthritis: cross-sectional and longitudinal approach. *Arthritis Rheum.* 2004;50:2471-2478.
**247** Ribeiro JC GS. Oliveira J. Andersen LB. Duarte JA. Mota J. Body fatness and clustering of cardiovascular disease risk factors in Portuguese children and adolescents. *Am J Hum Biol.* 2004;16:556-562.
**248** Richardson DB WS. Lorey F. Hertz-Picciotto I. Adult hemoglobin levels at birth and risk of sudden infant death syndrome. *Arch Pediatr Adolesc Med.* 2004;158:366-371.
**249** Ridker PM RN. Cook NR. Bradwin G. Buring JE. Non-HDL cholesterol. apolipoproteins A-I and B100. standard lipid measures. lipid ratios. and CRP as risk factors for cardiovascular disease in women. *JAMA.* 2005;294:326-333.
**250** Rinaldi S. Kaaks R. Zeleniuch-Jacquotte A. et al. Insulin-like growth factor-I. IGF binding protein-3. and breast cancer in young women: a comparison of risk estimates using different peptide assays. *Cancer Epidemiol Biomarkers Prev.* Jan 2005;14:48-52.
**251** Rock CL FS. Natarajan L. Thomson CA. Bardwell WA. Newman VA. Hollenbach KA. Jones L. Caan BJ. Pierce JP. Plasma carotenoids and recurrence-free survival in women with a history of breast cancer. *J Clin Oncol.* 2005;23:6631-6638.
**252** Rosengren A WL. Orth-Gomer K. Coronary disease in relation to social support and social class in Swedish men. A 15 year follow-up in the study of men born in 1933. *Eur Heart J.* 2004;25:56-63.
**253** Rosenthal AD JF. Shu XO. Yang G. Elasy TA. Chow WH. Ji BT. Xu HX. Li Q. Gao YT. Zheng W. Body fat distribution and risk of diabetes among Chinese women. *Int J Obes Relat Metab Disord.* 2004;28:594-599.
**254** Rossi R CE. Nuzzo A. Cioni E. Origliani G. Modena MG. Flow-mediated vasodilation and the risk of developing hypertension in healthy postmenopausal women. *J Am Coll Cardiol.* 2004;44:1636-1640.
**255** Rossi R CE. Nuzzo A. Origliani G. Modena MG. Endothelial-dependent vasodilation and incidence of type 2 diabetes in a population of healthy postmenopausal women. *Diabetes Care.* 2005;28:702-707.
**256** Rouillier P B-RM. Bertrais S. Arnault N. Daudin JJ. Bacro JN. Hercberg S. Alcohol and atherosclerotic vascular disease risk factors in French men: relationships are linear. J-shaped. and U-shaped. *Alcohol Clin Exp Res.* 2005;29:84-88.
**257** Rumbold AR MF. Crowther CA. Dietary intake of vitamin C and vitamin E and the development of hypertensive disorders of pregnancy. *Eur J Obstet Gynecol Reprod Biol.* 2005;119:67-71.
**258** Rusiecki JA DRA. Lee WJ. Dosemeci M. Lubin JH. Hoppin JA. Blair A. Alavanja MC. Cancer incidence among pesticide applicators exposed to atrazine in the Agricultural Health Study. *J Natl Cancer Inst.* 2004;96:1375-1382.
**259** Rutter MK MJ. Sullivan LM. D'Agostino RB Sr. Wilson PW. C-reactive protein. the 3037metabolic syndrome. and prediction of cardiovascular events in the Framingham Offspring Study. *Circulation.* 2004;110:380-385.

**260** Salvarani C CL. Macchioni P. Consonni D. Cantini F. Bajocchi G. Pipitone N. Catanoso MG. Boiardi L. Risk factors for visual loss in an Italian population-based cohort of patients with giant cell arteritis. *Arthritis Rheum.* 2005;53:293-297.
**261** Samelson EJ KD. Broe KE. Zhang Y. Cupples LA. Hannan MT. Wilson PW. Levy D. Williams SA. Vaccarino V. Metacarpal cortical area and risk of coronary heart disease: the Framingham Study. *Am J Epidemiol.* 2004;159:589-595.
**262** Sanchez PL MJ. Pabon P. Martin F. Piedra I. Palacios IF. Martin-Luengo C. Prognostic relations between inflammatory markers and mortality in diabetic patients with non-ST elevation acute coronary syndrome. *Heart.* 2004;90:264-269.
**263** Sato Y TY. Nakaya N. Ogawa K. Kurashima K. Kuriyama S. Hozawa A. Nishino Y. Shibuya D. Tsuji I. Fruit and vegetable consumption and risk of colorectal cancer in Japan: The Miyagi Cohort Study. *Public Health Nutr.* 2005;8:309-314.
**264** Saw SM TS. Fung D. Chia KS. Koh D. Tan DT. Stone RA. IQ and the association with myopia in children. *Invest Ophthalmol Vis Sci.* 2004;45:2493-2498.
**265** Schaumberg DA LS. Seddon JM. Willett WC. Hankinson SE. Dietary glycemic load and risk of age-related cataract. *Am J Clin Nutr.* 2004;80:489-495.
**266** Schernhammer ES HJ. Pollak MN. Hankinson SE. Circulating levels of insulin-like growth factors. their binding proteins. and breast cancer risk. *Cancer Epidemiol Biomarkers Prev.* 2005;14:699-704.
**267** Schernhammer ES HS. Urinary melatonin levels and breast cancer risk. *J Natl Cancer Inst.* 2005;97:1084-1087.
**268** Schillinger M EM. Sabeti S. Mlekusch W. Amighi J. Handler S. Quehenberger P. Kalifeh N. Wagner O. Minar E. High plasma heparin cofactor II activity protects from restenosis after femoropopliteal stenting. *Thromb Haemost.* 2004;95:1108-1113.
**269** Schnabel R RH. Lackner KJ. Lubos E. Bickel C. Meyer J. Munzel T. Cambien F. Tiret L. Blankenberg S; AtheroGene Investigators. Analysis of N-terminal-pro-brain natriuretic peptide and C-reactive protein for risk stratification in stable and unstable coronary artery disease: results from the AtheroGene study. *Eur Heart J.* 2005;26:241-249.
**270** Schoen RE WJ. Kuller LH. Thaete FL. Evans RW. Hayes RB. Rosen CJ. Insulin-like growth factor-I and insulin are associated with the presence and advancement of adenomatous polyps. *Gastroenterology.* 2005;129:464-475.
**271** Schuiling WJ dWA. Dennesen PJ. Algra A. Rinkel GJ. The simplified acute physiology score to predict outcome in patients with subarachnoid hemorrhage. *Neurosurgery.* 2005;57:230-236.
**272** Schulze MB HK. Manson JE. Willett WC. Meigs JB. Weikert C. Heidemann C. Colditz GA. Hu FB. Dietary pattern. inflammation. and incidence of type 2 diabetes in women. *Am J Clin Nutr.* 2005;82:675-684.
**273** Schulze MB LS. Rimm EB. Manson JE. Willett WC. Hu FB. Glycemic index. glycemic load. and dietary fiber intake and incidence of type 2 diabetes in younger and middle-aged women. *Am J Clin Nutr.* 2004;80:348-356.

**274** Schulze MB RE. Li T. Rifai N. Stampfer MJ. Hu FB. C-reactive protein and incident cardiovascular events among men with diabetes. *Diabetes Care.* 2004;27:889-894.
**275** Schulze MB SC. Rifai N. Cohen RM. Sparrow J. Hu FB. Manson JE. Hyperproinsulinaemia and risk of Type 2 diabetes mellitus in women. *Diabet Med.* 2005;22:1178-1184.
**276** Schulze MB SI. Manson JE. Li T. Rifai N. Jiang R. Hu FB. Joint role of non-HDL cholesterol and glycated haemoglobin in predicting future coronary heart disease events among women with type 2 diabetes. *Diabetologia.* 2004;47:2129-2136.
**277** Schupf N CR. Luchsinger J. Tang MX. Lee JH. Mayeux R. Relationship between plasma lipids and all-cause mortality in nondemented elderly. *J Am Geriatr Soc.* 2005;53:219-226.
**278** Seddon JM GS. Rosner B. Rifai N. Progression of age-related macular degeneration: prospective assessment of C-reactive protein. interleukin 6. and other cardiovascular biomarkers. *Arch Ophthalmol.* 2005;123:774-782.
**279** Selvin E CJ. Golden SH. Brancati FL. Folsom AR. Steffes MW. Glycemic control and coronary heart disease risk in persons with and without diabetes: the atherosclerosis risk in communities study. *Arch Intern Med.* 2005;165:1910-1916.
**280** Selvin E CJ. Golden SH. Boland LL. Brancati FL. Steffes MW; Atherosclerosis risk in communities study. Glycemic control. atherosclerosis. and risk factors for cardiovascular disease in individuals with diabetes: the atherosclerosis risk in communities study. *Diabetes Care.* 2005;28:1965-1973.
**281** Sesso HD BJ. Norkus EP. Gaziano JM. Plasma lycopene. other carotenoids. and retinol and the risk of cardiovascular disease in women. *Am J Clin Nutr.* 2004;79:47-53.
**282** Sesso HD BJ. Zhang SM. Norkus EP. Gaziano JM. Dietary and plasma lycopene and the risk of breast cancer. *Cancer Epidemiol Biomarkers Prev.* 2005;14:1074-1081.
**283** Shai I SM. Manson JE. Stampfer MJ. Rifai N. Hu FB. A prospective study of lipoprotein(a) and risk of coronary heart disease among women with type 2 diabetes. *Diabetologia.* 2005;48:1469-1476.
**284** Shalak LF PJ. Jackson GL. Laptook AR. Depression at birth in term infants exposed to maternal chorioamnionitis: does neonatal fever play a role? *J Perinatol.* . 2005;25:447-452.
**285** Shankar A KB. Klein R. Relationship between white blood cell count and incident hypertension. *Am J Hypertens.* 2004;17:233-239.
**286** Sheehan J PI. Reilly M. Salim A. Collins M. Twomey EM. Daly A. Loingsigh SN. Elwood P. Ben-Shlomo Y. Davey-Smith G. QT dispersion. QT maximum and risk of cardiac death in the Caerphilly Heart Study. *Eur J Cardiovasc Prev Rehabil.* 2004;11:63-68.
**287** Shy BD RT. Becker LJ. Eisenberg MS. Time to intubation and survival in prehospital cardiac arrest. *Prehosp Emerg Care.* 2004;8:394-398.
**288** Sieri S. Krogh V. Pala V. et al. Dietary patterns and risk of breast cancer in the ORDET cohort. *Cancer Epidemiol Biomarkers Prev.* Apr 2004;13(4):567-572.
**289** Silvera SA JM. Howe GR. Miller AB. Rohan TE. Dietary carbohydrates and breast cancer risk: a prospective study of the roles of overall glycemic index and glycemic load. *Int J Cancer.* 2005;114:653-658.

**290** Silvera SA RT. Jain M. Terry PD. Howe GR. Miller AB. Glycemic index. glycemic load. and pancreatic cancer risk (Canada). *Cancer Causes Control.* 2005;16:431-436.
**291** Sin DD WL. Man SF. The relationship between reduced lung function and ardiovascular mortality: a population-based study and a systematic review of the literature. *Chest.* 2005;127:1952-1959.
**292** Singh JA ND. Fink HA. Nichol KL. Health-related quality of life predicts future health care utilization and mortality in veterans with self-reported physician-diagnosed arthritis: the veterans arthritis quality of life study. *Semin Arthritis Rheum.* . 2005;34:755-765.
**293** Singh SM PL. Li C. He J. Vinden C. Rabeneck L. Association of socioeconomic status and receipt of colorectal cancer investigations: a population-based retrospective cohort study. *CMAJ.* 2004;171(5):461-465.
**294** Skuladottir H TA. Overvad K. Stripp C. Christensen J. Raaschou-Nielsen O. Olsen JH. Does insufficient adjustment for smoking explain the preventive effects of fruit and vegetables on lung cancer? *Lung Cancer.* 2004;45:1-10.
**295** Slinin Y FR. Collins AJ. Calcium. phosphorus. parathyroid hormone. and cardiovascular disease in hemodialysis patients: the USRDS waves 1. 3. and 4 study. *J Am Soc Nephrol.* 2005;16:1788-1793.
**296** Smith W WJ. Wong TY. Rochtchina E. Klein R. Leeder SR. Mitchell P. Retinal arteriolar narrowing is associated with 5-year incident severe hypertension: the Blue Mountains Eye Study. *Hypertension.* 2004;44:442-447.
**297** Song Y MJ. Buring JE. Liu S. Dietary magnesium intake in relation to plasma insulin levels and risk of type 2 diabetes in women. *Diabetes Care.* 2004;27:59-65.
**298** Song Y MJ. Cook NR. Albert CM. Buring JE. Liu S. Dietary Magnesium Intake and Risk of Cardiovascular Disease Among Women. *Am J Cardiol.* 2005;96:1135-1141.
**299** Sornay-Rendu E MF. Duboeuf F. Delmas PD. Rate of Forearm Bone Loss Is Associated With an Increased Risk of Fracture Independently of Bone Mass in Postmenopausal Women: The OFELY Study. *J Bone Miner Res.* . 2005;20:1929-1935.
**300** Sornay-Rendu E MF. Garnero P. Duboeuf F. Delmas PD. Identification of Osteopenic Women at High Risk of Fracture: The OFELY Study. *J Bone Miner Res.* 2005;20:1813-1819.
**301** Sprenkle MD ND. Nelson DB. Nichol KL. The Veterans Short Form 36 questionnaire is predictive of mortality and health-care utilization in a population of veterans with a self-reported diagnosis of asthma or COPD. *Chest.* 2004;126:81-89.
**302** Stack AG MB. Molony DA. Survival differences between peritoneal dialysis and hemodialysis among "large" ESRD patients in the United States. *Kidney Int.* 2004;65(6):2398-2408.
**303** Stattin P BA. Biessy C. Kaaks R. Hallmans G. Adlercreutz H. Prospective study of plasma enterolactone and prostate cancer risk (Sweden). *Cancer Causes Control.* 2004;15:1095-1102.
**304** Stattin P LS. Tenkanen L. Alfthan H. Jellum E. Hallmans G. Thoresen S. Hakulinen T. Luostarinen T. Lehtinen M. Dillner J. Stenman UH. Hakama M. High levels of circulating testosterone are not associated with increased prostate cancer risk: a pooled prospective study. *Int J Cancer.* 2004;108:418-424.

**305** Stattin P RS. Biessy C. Stenman UH. Hallmans G. Kaaks R. High levels of circulating insulin-like growth factor-I increase prostate cancer risk: a prospective study in a population-based nonscreened cohort. *J Clin Oncol.* 2004;22:3104-3112.
**306** Stolzenberg-Solomon RZ LP. Pollak M. Taylor PR. Virtamo J. Albanes D. Insulin-like growth factor (IGF)-1. IGF-binding protein-3. and pancreatic cancer in male smokers. *Cancer Epidemiol Biomarkers Prev.* 2004;13:438-444.
**307** Storti KL BJ. Fitzgerald SJ. Zmuda JM. Cauley JA. Kriska AM. Physical activity and decreased risk of clinical gallstone disease among post-menopausal women. *Prev Med.* . 2005;41(3-4):772-777.
**308** St-Pierre AC CB. Dagenais GR. Mauriege P. Bernard PM. Despres JP. Lamarche B. Low-density lipoprotein subfractions and the long-term risk of ischemic heart disease in men: 13-year follow-up data from the Quebec Cardiovascular Study. *Arterioscler Thromb Vasc Biol.* 2005;25:553-559.
**309** St-Pierre AC CB. Dagenais GR. Mauriege P. Bernard PM. Despres JP. Lamarche B. Low-density lipoprotein subfractions and the long-term risk of ischemic heart disease in men: 13-year follow-up data from the Quebec Cardiovascular Study. *Arterioscler Thromb Vasc Biol.* 2005;25:353-359.
**310** Suzuki K IY. Wakai K. Kawado M. Hashimoto S. Toyoshima H. Kojima M. Tokudome S. Hayakawa N. Watanabe Y. Tamakoshi K. Suzuki S. Ozasa K. Tamakoshi A; Japan Collaborative Cohort Study Group. Serum oxidized low-density lipoprotein levels and risk of colorectal cancer: a case-control study nested in the Japan Collaborative Cohort Study. *Cancer Epidemiol Biomarkers Prev.* 2004;13:1781-1787.
**311** Svensson AM MD. Abrahamsson P. Dellborg M. Association between hyper- and hypoglycaemia and 2 year all-cause mortality risk in diabetic patients with acute coronary events. *Eur Heart J.* 2005;26:1255-1261.
**312** Sweeney C BC. Anderson KE. Lazovich D. Folsom AR. Risk factors for breast cancer in elderly women. *Am J Epidemiol.* 2004;160:868-875.
**313** Takebayashi T NY. Uemura T. Nakashima H. Nomiyama T. Sakurai H. Omae K. A six year follow up study of the subclinical effects of carbon disulphide exposure on the cardiovascular system. *Occup Environ Med.* 2004;61:127-134.
**314** Tan ZS SS. Beiser A. Zhang Y. Felson D. Hannan MT. Au R. Wolf PA. Kiel DP. Bone mineral density and the risk of Alzheimer disease. *Arch Neurol.* 2005;62:107-111.
**315** Tanne D MJ. Goldbourt U. Body fat distribution and long-term risk of stroke mortality. *Stroke.* 2005;35:1021-1025.
**316** Tarnow L AA. Parving HH. Elevated placental growth factor (PlGF) predicts cardiovascular morbidity and mortality in type 1 diabetic patients with diabetic nephropathy. *Scand J Clin Lab Invest Suppl.* 2005;240:73-79.
**317** Tarnow L HP. Hansen BV. Borch-Johnsen K. Parving HH. Plasma N-terminal pro-brain natriuretic peptide as an independent predictor of mortality in diabetic nephropathy. *Diabetologia.* 2005;48:149-155.
**318** Taylor EN SM. Curhan GC. Dietary factors and the risk of incident kidney stones in men: new insights after 14 years of follow-up. *J Am Soc Nephrol.* 2004;15:3225-3232.
**319** Taylor EN SM. Curhan GC. Fatty acid intake and incident nephrolithiasis. *Am J Kidney Dis.* 2005;45:267-274.

**320** Taylor HA Jr CB. Garrison RJ. Andrew ME. Han H. Fox ER. Arnett DK. Samdarshi T. Jones DW. Relation of aortic valve sclerosis to risk of coronary heart disease in African-Americans. *Am J Cardiol.* 2005;95:401-404.

**321** Thomson CC FL. Winickoff JP. Colditz GA. Camargo CA Jr. King C 3rd. Frazier AL. State tobacco excise taxes and adolescent smoking behaviors in the United States. *J Public Health Manag Pract.* 2004;10:490-496.

**322** Thorand B KH. Baumert J. Koenig W. Chambless L. Meisinger C. Illig T. Martin S. Herder C. Elevated levels of interleukin-18 predict the development of type 2 diabetes: results from the MONICA/KORA Augsburg Study. 1984-2002. *Diabetes.* 2005;54:2392-2398.

**323** Tolmunen T HJ. Ruusunen A. Voutilainen S. Tanskanen A. Valkonen VP. Viinamaki H. Kaplan GA. Salonen JT. Dietary folate and the risk of depression in Finnish middle-aged men. A prospective follow-up study. *Psychother Psychosom.* 2004;73:334-339.

**324** Tolmunen T HJ. Voutilainen S. Ruusunen A. Alfthan G. Nyyssonen K. Viinamaki H. Kaplan GA. Salonen JT. Association between depressive symptoms and serum concentrations of homocysteine in men: a population study. *Am J Clin Nutr.* 2004;80:1574-1578.

**325** Touvier M KE. Clavel-Chapelon F. Boutron-Ruault MC. Dual Association of beta-carotene with risk of tobacco-related cancers in a cohort of French women. *J Natl Cancer Inst.* 2005;97:1338-1344.

**326** Tripepi G MF. Zoccali C. Inflammation markers. adhesion molecules. and all-cause and cardiovascular mortality in patients with ESRD: searching for the best risk marker by multivariate modeling. *J Am Soc Nephrol.* 2005;16:S93-98.

**327** Tsai CJ. Leitzmann MF. Willett WC. Giovannucci EL. Long-term intake of dietary fiber and decreased risk of cholecystectomy in women. *Am J Gastroenterol.* Jul 2004;99:1364-1370.

**328** Tsai CJ LM. Willett WC. Giovannucci EL. The effect of long-term intake of cis unsaturated fats on the risk for gallstone disease in men: a prospective cohort study. *Ann Intern Med.* 2004;141:514-522.

**329** Tsai CJ LM. Willett WC. Giovannucci EL. Dietary protein and the risk of cholecystectomy in a cohort of US women: the Nurses' Health Study. *Am J Epidemiol.* 2004;160:11-18.

**330** Tsai CJ LM. Willett WC. Giovannucci EL. Glycemic load. glycemic index. and carbohydrate intake in relation to risk of cholecystectomy in women. *Gastroenterology.* 2005;129:105-112.

**331** Tsai CJ LM. Willett WC. Giovannucci EL. Dietary carbohydrates and glycaemic load and the incidence of symptomatic gall stone disease in men. *Gut.* 2005;54:823-828.

**332** Tsai CJ LM. Willett WC. Giovannucci EL. Long-term intake of trans-fatty acids and risk of gallstone disease in men. *Arch Intern Med.* 2005;165:1011-1015.

**333** Tseng M BR. DeVellis RF. Ziegler RG. Dietary patterns and prostate cancer risk in the National Health and Nutrition Examination Survey Epidemiological Follow-up Study cohort. . *Cancer Epidemiol Biomarkers Prev.* . 2004;13:71-77.

**334** Tseng M BR. Graubard BI. Ziegler RG. Dairy. calcium. and vitamin D intakes and prostate cancer risk in the National Health and Nutrition Examination Epidemiologic Follow-up Study cohort. *Am J Clin Nutr.* 2005;81:1147-1154.

**335** Tsimikas S BE. Miller ER. McConnell JP. Lennon RJ. Kornman KS. Witztum JL. Berger PB. Oxidized phospholipids. Lp(a) lipoprotein. and coronary artery disease. *N Engl J Med.* 2005;353:46-57.
**336** Tsubono Y OT. Kobayashi M. Yamamoto S. Sobue T. Tsugane S; JPHC Study Group. No association between fruit or vegetable consumption and the risk of colorectal cancer in Japan. *Br J Cancer.* 2005;92:1782-1784.
**337** Tsushima M NA. Lee J. Stemmermann GN. Prospective study of the association of serum triglyceride and glucose with colorectal cancer. *Dig Dis Sci.* 2005;50:499-505.
**338** Turner BJ WM. Yang C. TenHave T. Predicting adherence to colonoscopy or flexible sigmoidoscopy on the basis of physician appointment-keeping behavior. *Ann Intern Med.* 2004;140:528-532.
**339** Tworoger SS EA. Rosner B. Sluss P. Hankinson SE. Plasma prolactin concentrations and risk of postmenopausal breast cancer. *Cancer Res.* 2004;64:6814-6819.
**340** Tworoger SS MS. Barbieri RL. Willett WC. Colditz GA. Hankinson SE. Plasma sex hormone concentrations and subsequent risk of breast cancer among women using postmenopausal hormones. *J Natl Cancer Inst.* 2005;97:595-602.
**341** Ulvik A VS. Hansen S. Gislefoss R. Jellum E. Ueland PM. Colorectal cancer and the methylenetetrahydrofolate reductase 677C -> T and methionine synthase 2756A -> G polymorphisms: a study of 2.168 case-control pairs from the JANUS cohort. *Cancer Epidemiol Biomarkers Prev.* 2004;13:2175-2180.
**342** Van Den Brink CL PH. Van Den Bos GA. Giampaoli S. Nissinen A. Kromhout D. Duration and intensity of physical activity and disability among European elderly men. *Disabil Rehabil.* 2005;27:341-347.
**343** van der A DL GD. Roest M. Marx JJ. Voorbij HA. van der Schouw YT. Serum ferritin is a risk factor for stroke in postmenopausal women. *Stroke.* 2005;36:1637-1641.
**344** van Gelder BM TM. Kalmijn S. Giampaoli S. Nissinen A. Kromhout D. Physical activity in relation to cognitive decline in elderly men: the FINE Study. *Neurology.* 2004;63: 2316-21.
**345** van Gils CH PP. Bueno-de-Mesquita HB. Boshuizen HC. Lahmann PH. Clavel-Chapelon F. Thiebaut A. Kesse E. Sieri S. Palli D. Tumino R. Panico S. Vineis P. Gonzalez CA. Ardanaz E. Sanchez MJ. Amiano P. Navarro C. Quiros JR. Key TJ. Allen N. Khaw KT. Bingham SA. Psaltopoulou T. Koliva M. Trichopoulou A. Nagel G. Linseisen J. Boeing H. Berglund G. Wirfalt E. Hallmans G. Lenner P. Overvad K. Tjonneland A. Olsen A. Lund E. Engeset D. Alsaker E. Norat T. Kaaks R. Slimani N. Riboli E. Consumption of vegetables and fruits and risk of breast cancer. *JAMA.* 2005;293:183-193.
**346** Van Guelpen B HJ. Johansson I. Stegmayr B. Hallmans G. Nilsson TK. Weinehall L. Witthoft C. Palmqvist R. Winkvist A. Folate. vitamin B12. and risk of ischemic and hemorrhagic stroke: a prospective. nested case-referent study of plasma concentrations and dietary intake. *Stroke.* 2005;36:1426-1431.
**347** van Hecke MV DJ. Nijpels G. Moll AC. Heine RJ. Bouter LM. Polak BC. Stehouwer CD. Inflammation and endothelial dysfunction are associated with retinopathy: the Hoorn Study. *Diabetologia.* 2005;48:1300-1306.
**348** van Meurs JB D-RR. Pluijm SM. van der Klift M. de Jonge R. Lindemans J. de Groot LC. Hofman A. Witteman JC. van Leeuwen JP. Breteler MM. Lips

P. Pols HA. Uitterlinden AG. Homocysteine levels and the risk of osteoporotic fracture. *N Engl J Med.* 2004;350:2033-2041.

**349** Verma A. Kilicaslan F. Martin DO. et al. Preimplantation B-type natriuretic peptide concentration is an independent predictor of future appropriate implantable defibrillator therapies. *Heart.* Feb 2006;92:190-195.

**350** Visser M GB. Kritchevsky SB. Newman AB. Nevitt M. Rubin SM. Simonsick EM. Harris TB. Muscle mass. muscle strength. and muscle fat infiltration as predictors of incident mobility limitations in well-functioning older persons. *J Gerontol A Biol Sci Med Sci.* 2005;60:324-333.

**351** Voutilainen S VJ. Rissanen TH. Alfthan G. Laukkanen J. Nyyssonen K. Mursu J. Valkonen VP. Tuomainen TP. Kaplan GA. Salonen JT. Serum folate and homocysteine and the incidence of acute coronary events: the Kuopio Ischaemic Heart Disease Risk Factor Study. *Am J Clin Nutr.* 2004;80:317-323.

**352** Vrbova L MM. Moineddin R. Jaakimainen L. Upshur RE. Does socioeconomic status affect mortality subsequent to hospital admission for community acquired pneumonia among older persons? *J Negat Results Biomed.* 2005;4:4.

**353** Vriesendorp TM MQ. Devries JH. Legemate DA. Hoekstra JB. Early post-operative glucose levels are an independent risk factor for infection after peripheral vascular surgery. A retrospective study. *Eur J Vasc Endovasc Surg.* 2004;28:520-525.

**354** Wakai K SK. Ito Y. Kojima M. Tamakoshi K. Watanabe Y. Toyoshima H. Hayakawa N. Hashimoto S. Tokudome S. Suzuki S. Kawado M. Ozasa K. Tamakoshi A; Japan Collaborative Cohort Study Group. Serum carotenoids. retinol. and tocopherols. and colorectal cancer risk in a Japanese cohort: effect modification by sex for carotenoids. *Nutr Cancer.* 2005;51:13-24.

**355** Wakai K TK. Date C. Fukui M. Suzuki S. Lin Y. Niwa Y. Nishio K. Yatsuya H. Kondo T. Tokudome S. Yamamoto A. Toyoshima H. Tamakoshi A; JACC Study Group. Dietary intakes of fat and fatty acids and risk of breast cancer: a prospective study in Japan. *Cancer Sci.* 2005;96:590-599.

**356** Wang AY SM. Tang N. Sanderson JE. Lui SF. Li PK. Woo J. Resting energy expenditure and subsequent mortality risk in peritoneal dialysis patients. *J Am Soc Nephrol.* 2004;15:3134-3143.

**357** Wang J. Work stress as a risk factor for major depressive episode(s). *Psychol Med.* 2005;35:865-871.

**358** Wang TJ EJ. Meigs JB. Rifai N. Fox CS. D'Agostino RB. Levy D. Vasan RS. Low-grade albuminuria and the risks of hypertension and blood pressure progression. *Circulation.* 2005;111:1370-1376.

**359** Wang Y RE. Stampfer MJ. Willett WC. Hu FB. Comparison of abdominal adiposity and overall obesity in predicting risk of type 2 diabetes among men. *Am J Clin Nutr.* 2005;81:555-563.

**360** Wannamethee SG LD. Whincup PH. Walker M. Ebrahim S. Davey-Smith G. Birthweight of offspring and paternal insulin resistance and paternal diabetes in late adulthood: cross sectional survey. *Diabetologia.* 2004;47:12-18.

**361** Wark PA WM. van 't Veer P. van Wijhe G. Luchtenborg M. van Muijen GN. de Goeij AF. Goldbohm RA. van den Brandt PA. Fruits. vegetables. and hMLH1 protein-deficient and -proficient colon cancer: The Netherlands cohort study. *Cancer Epidemiol Biomarkers Prev.* 2005;14:1619-1625.

**362** Wattanakit K FA. Selvin E. Weatherley BD. Pankow JS. Brancati FL. Hirsch AT. Risk factors for peripheral arterial disease incidence in persons with

diabetes: the Atherosclerosis Risk in Communities (ARIC) Study. *Atherosclerosis.* 2005;180:389-397.

**363** Weinstein AR SH. Lee IM. Cook NR. Manson JE. Buring JE. Gaziano JM. Relationship of physical activity vs body mass index with type 2 diabetes in women. *JAMA.* 2004;292:1188-1194.

**364** Weinstein SJ. Wright ME. Pietinen P. et al. Serum alpha-tocopherol and gamma-tocopherol in relation to prostate cancer risk in a prospective study. *J Natl Cancer Inst.* Mar 2 2005;97:396-399.

**365** Whittemore AS CP. Feldman D. Cohn BA. Prostate specific antigen levels in young adulthood predict prostate cancer risk: results from a cohort of Black and White Americans. *J Urol.* 2005;174:872-876.

**366** Wolf M SJ. Shah A. Vossen Smirnakis K. Jimenez-Kimble R. Ecker JL. Thadhani R. Inflammation and glucose intolerance: a prospective study of gestational diabetes mellitus. *Diabetes Care.* 2004;27:21-27.

**367** Wolk R BP. Lennon RJ. Brilakis ES. Johnson BD. Somers VK. Plasma leptin and prognosis in patients with established coronary atherosclerosis. *J Am Coll Cardiol.* 2004;44:1819-1824.

**368** Wong TY KM. Klein R. Klein BE. Hubbard LD. A prospective cohort study of retinal arteriolar narrowing and mortality. *Am J Epidemiol.* 2004;159:819-825.

**369** Wong TY KR. Sharrett AR. Duncan BB. Couper DJ. Klein BE. Hubbard LD. Nieto FJ; Atherosclerosis Risk in Communities Study. Retinal arteriolar diameter and risk for hypertension. *Ann Intern Med.* 2004;140:248-255.

**370** Wong TY SA. Klein R. Klein BE. Retinal vessel diameters and the incidence of gross proteinuria and renal insufficiency in people with type 1 diabetes. *Diabetes.* 2004;53:179-184.

**371** Wong TY SA. Klein R. Klein BE. Hubbard LD. Retinal arteriolar narrowing. hypertension. and subsequent risk of diabetes mellitus. *Arch Intern Med.* 2005;165:1060-1065.

**372** Wrensch M WA. Wiencke J. Miike R. Sison J. Wiemels J. Barger G. DeLorenze G. Aldape K. Kelsey K. History of chickenpox and shingles and prevalence of antibodies to varicella-zoster virus and three other herpesviruses among adults with glioma and controls. *Am J Epidemiol.* 2005;161:929-938.

**373** Wright ME MD. Pietinen P. Taylor PR. Virtamo J. Albanes D. Estimated Urine pH and Bladder Cancer Risk in a Cohort of Male Smokers (Finland)*. *Cancer Causes Control.* 2005;16:1117-1123.

**374** Wright ME MS. Stolzenberg-Solomon RZ. Li Z. Pietinen P. Taylor PR. Virtamo J. Albanes D. Development of a comprehensive dietary antioxidant index and application to lung cancer risk in a cohort of male smokers. *Am J Epidemiol.* 2004;160:68-76.

**375** Wu K HF. Fuchs C. Rimm EB. Willett WC. Giovannucci E. Dietary patterns and risk of colon cancer and adenoma in a cohort of men (United States). *Cancer Causes Control.* 2004;15:853-862.

**376** Yamagata H KY. Nakamura S. Kubo M. Tanizaki Y. Matsumoto T. Tanaka K. Kato I. Shirota T. Iida M. mpact of fasting plasma glucose levels on gastric cancer incidence in a general Japanese population: the Hisayama study. *Diabetes Care.* 2005;28:789-794.

**377** Yang G SX. Jin F. Elasy T. Li HL. Li Q. Huang F. Zhang XL. Gao YT. Zheng W.Yang G. Shu XO. Jin F. Elasy T. Li HL. Li Q. Huang F. Zhang XL. Gao YT. Zheng W. Soyfood consumption and risk of glycosuria: a cross-sectional

study within the Shanghai Women's Health Study. *Eur J Clin Nutr.* 2004;58:615-620.

**378** Yeh HC PN. Wang NY. Pankow JS. Duncan BB. Brancati FL. Vital capacity as a predictor of incident type 2 diabetes: the Atherosclerosis Risk in Communities study. *Diabetes Care.* 2005;28:1472-1479.

**379** Yende S. Tuomanen EI. Wunderink R. et al. Preinfection systemic inflammatory markers and risk of hospitalization due to pneumonia. *Am J Respir Crit Care Med.* Dec 1 2005;172:1440-1446.

**380** Yuan JM RR. Gao YT. Qu YH. Chu XD. Yu MC. Prediagnostic levels of serum micronutrients in relation to risk of gastric cancer in Shanghai. China. *Cancer Epidemiol Biomarkers Prev.* 2004;13:1772-1780.

**381** Zeleniuch-Jacquotte A AH. Shore RE. Koenig KL. Kato I. Arslan AA. Toniolo P. Circulating enterolactone and risk of breast cancer: a prospective study in New York. *Br J Cancer.* 2004;91:99-105.

**382** Zeleniuch-Jacquotte A GY. Shore RE. Koenig KL. Arslan AA. Kato I. Rinaldi S. Kaaks R. Toniolo P. Postmenopausal levels of sex hormones and risk of breast carcinoma in situ: results of a prospective study. *Int J Cancer.* . 2004;114:323-327.

**383** Zeleniuch-Jacquotte A SR. Koenig KL. Akhmedkhanov A. Afanasyeva Y. Kato I. Kim MY. Rinaldi S. Kaaks R. Toniolo P. Postmenopausal levels of oestrogen. androgen. and SHBG and breast cancer: long-term results of a prospective study. *Br J Cancer.* 2004;90:153-159.

**384** Zhang C WM. Sorensen TK. King IB. Kestin MM. Thompson ML. Leisenring WM. Dashow EE. Luthy DA. Maternal plasma ascorbic Acid (vitamin C) and risk of gestational diabetes mellitus. *Epidemiology.* 2004;15:597-604.

**385** Zhang C WM. Frederick IO. King IB. Sorensen TK. Kestin MM. Dashow EE. Luthy DA. Vitamin C and the risk of gestational diabetes mellitus: a case-control study. *J Reprod Med.* 2004;49:257-266.

**386** Zhang SM HS. Hunter DJ. Giovannucci EL. Colditz GA. Willett WC. Folate intake and risk of breast cancer characterized by hormone receptor status. *Cancer Epidemiol Biomarkers Prev.* 2005;14:2004-2008.

**387** Zhang X SX. Gao YT. Yang G. Matthews CE. Li Q. Li H. Jin F. Zheng W. Anthropometric predictors of coronary heart disease in Chinese women. *Int J Obes Relat Metab Disord.* 2004;28:734-740.

**388** Zhang X SX. Li H. Yang G. Li Q. Gao YT. Zheng W. Prospective cohort study of soy food consumption and risk of bone fracture among postmenopausal women. *Arch Intern Med.* 2005;165:1890-1895.

**389** Zylberstein DE BC. Bjorkelund C. Landaas S. Sundh V. Thelle D. Lissner L. Serum homocysteine in relation to mortality and morbidity from coronary heart disease: a 24-year follow-up of the population study of women in Gothenburg. *Circulation.* 2004;109:601-606.
